# New *ψ*-Santonin Derivatives from *Crossostephium chinense* and Their Anti-Proliferative Activities against *Leishmania major* and Human Cancer Cells A549

**DOI:** 10.3390/molecules28248108

**Published:** 2023-12-15

**Authors:** Zhichao Wang, Yoshi Yamano, Susumu Kawakami, Gadah Abdulaziz Al-Hamoud, Sachiko Sugimoto, Hideaki Otsuka, Katsuyoshi Matsunami

**Affiliations:** 1 Graduate School of Biomedical & Health Sciences, Hiroshima University, 1-2-3 Kasumi, Minami-Ku, Hiroshima 734-8553, Japan; zhichao96425@gmail.com (Z.W.); yamano@hiroshima-u.ac.jp (Y.Y.); ssugimot@hiroshima-u.ac.jp (S.S.); 2 Graduate School of Pharmacy, Yasuda Women’s University, 6-13-1 Yasuhigashi, Asaminami-Ku, Hiroshima 731-0153, Japan; kawakami@yasuda-u.ac.jp (S.K.); otsuka-h@yasuda-u.ac.jp (H.O.); 3 Department of Pharmacognosy, College of Pharmacy, King Saud University, Riyadh 11451, Saudi Arabia; galhamoud@ksu.edu.sa

**Keywords:** *Crossostephium chinense*, sesquiterpene, *ψ*-santonin, *Leishmania major*, A549

## Abstract

Previously, we reported two cytotoxic *ψ*-santonin–amino acid conjugates isolated from the EtOAc layer of *Crossostephium chinense*. However, a further phytochemical investigation seems to be required because of the few reports of similar derivatives. In this study, we targeted the 1-BuOH layer, which resulted in the isolation of seven new *ψ*-santonin derivatives (**1**–**7**) together with ten known compounds (**8**–**17**). The structures of **1**–**7** were elucidated based on spectroscopic methods, including 1D and 2D NMR experiments (^1^H, ^13^C, DEPT, COSY, HSQC, and HMBC), IR spectrum, and high-resolution electrospray ionization–mass spectrometry (HR-ESI–MS). The stereochemistry of new compounds was confirmed by NOESY and ECD calculations. All isolated compounds were evaluated by in vitro experiments for their anti-proliferative activities against *Leishmania major*, human lung cancer cell line A549, and Vero cells. As a result, most of the *ψ*-santonin derivatives, especially **1**–**5**, showed significant cytotoxicity against *L. major* with a lower IC_50_ than the positive control we used (miltefosine).

## 1. Introduction

Leishmaniasis is a tropical disease caused by at least 20 species of protozoan parasites from the Family trypanosomatidae and is transmitted by the bite of infected female sandflies. According to the reports from WHO, more than 350 million people are at risk, about 12 million of whom are infected, with an average annual incidence of one million new cases, mainly from low-income developing countries [1]. To accelerate global drug development, WHO has designated leishmaniasis as one of the neglected tropical diseases (NTDs) and set a new roadmap for 2021–2030 to drive progress towards a world free of NTDs by 2030. Leishmaniasis has various clinical manifestations, which can be classified into cutaneous leishmaniasis (CL), visceral leishmaniasis (VL), and mucocutaneous leishmaniasis (MCL) [2,3]. *Leishmania major* (*L. major*) is one of the species that causes CL, which is not fatal but is responsible for disfiguring lesions and social stigma [4]. The symptoms after the sandfly bite begin from a small edema to a papule and finally develop into a nodule that grows into necrosis and ulceration [5]. There are about 600,000 to 1 million new cases of CL annually, 75% of which are diagnosed in Afghanistan, Brazil, Iran, Iraq, and Syria [6]. Many anticancer drugs have been developed and used clinically for years. However, cancer is still a leading cause of death in many countries. Among various cancers, lung cancer causes the most deaths worldwide [7]. A549 is a cell line derived from human lung cancer and is frequently used in anticancer drug screening.

**Figure 1:**
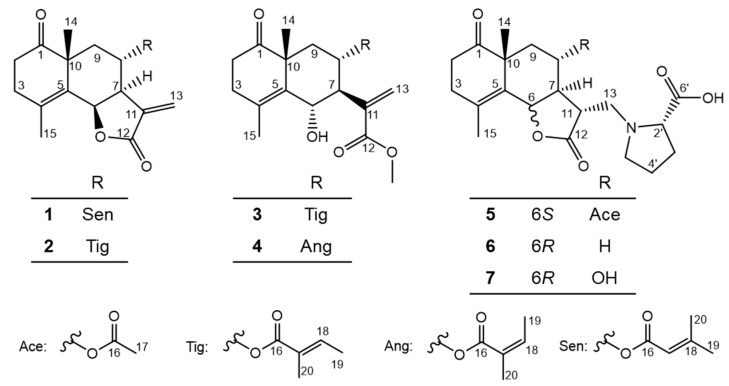
Structures of new *ψ*-santonin derivatives (**1**–**7**) from *C. chinense*.

*Crossostephium chinense* is an evergreen shrub belonging to the Asteraceae family and distributed in Japan, China, and other Asian countries. The Family Asteraceae is famous for its promising source of bioactive compounds, such as pyrethrin, artemisinin, and santonin from the plant *Tanacetum cinerariifolium*, *Artemisia annua*, and *Artemisia maritima*, respectively. Our research team focuses on discovering bioactive constituents against *Leishmania major* from natural resources. *C. chinense* was selected based on the primary screening results, and then, two *ψ*-santonin–amino acid conjugates that showed potent cytotoxicity against *L. major* named crossoseamine A and B were previously discovered from the EtOAc layer [7]. However, a further phytochemical investigation of *C. chinense* seems necessary because of the few reports of *ψ*-santonin derivatives and their potential bioactivities. Therefore, we targeted the 1-BuOH layer of *C. chinense*, aiming to isolate more novel *ψ*-santonin derivatives in this study. As a result, seven previously undescribed compounds (**1**–**7**) (Figure 1) together with ten known compounds (**8**–**17**) were isolated (Figure S1), of which the new *ψ*-santonin derivatives **1**–**5** showed significant cytotoxic activities against *L. major*. In addition, the A549 cancer and Vero cell lines were used to evaluate the cytotoxicity of all isolated compounds.

## 2. Results and Discussion

### 2.1. Structure Determination of New Compounds ***1***–***7***

#### 2.1.1. Structure of Crossostenin A (**1**)

**Figure 2:**
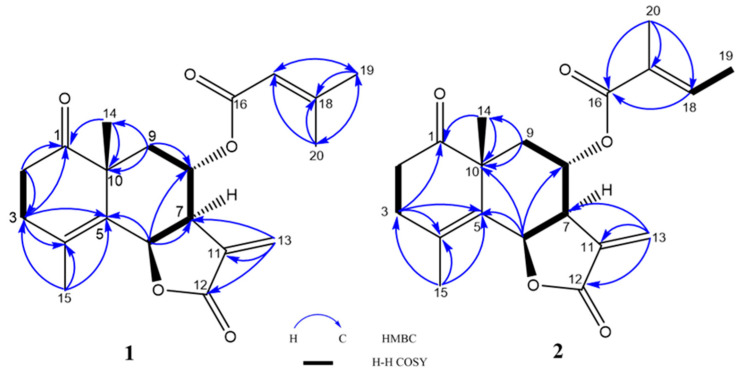
Important ^1^H-^1^H COSY and HMBC correlations of **1** and **2**.

Crossostenin A (**1**) was isolated as a white amorphous powder with a molecular formula C_20_H_24_O_5_ established by HR-ESI-MS data, which showed a positive ion [M + Na]^+^ at *m*/*z* 367.1515 (calculated for C_20_H_24_O_5_Na: 367.1516). The IR spectrum contained absorption signals at 1765 and 1710 cm^−1^ due to several carbonyl functions, of which 1765 cm^−1^ suggested a γ-lactone functional group and 1710 cm^−1^ due to ester/lactone/ketone carbonyl functions. The ^13^C NMR (Table 1) spectrum revealed the existence of three carbonyl groups (δ_C_ 166.9, 171.9, and 216.4) and three double bonds (δ_C_ 116.2, 125.8, 128.1, 139.1, 141.1, and 160.1), which provided six indices of hydrogen deficiency. According to the other signals, such as four methine carbons (including two oxygenated carbons at δ_C_ 72.7 and 77.5 and one olefinic at δ_C_ 116.2), four methylene (including one olefinic carbon at δ_C_ 125.8) and four methyl carbons (δ_C_ 20.0, 20.5, 26.2 and 27.6), the remaining three unsaturation degrees were ascribed to a three-ring skeleton, which is similar to the crossoseamine B previously isolated from the titled plant in our previous study [7]. Correspondingly, there were four olefinic protons (δ_H_ 5.49, 1H, sept, *J* = 1.4 Hz, H-17; δ_H_ 5.90, 1H, d, *J* = 2.0 Hz, H-13a; δ_H_ 6.34, 1H, d, *J* = 2.0 Hz, H-13b) and two oxygenated methine protons (δ_H_ 5.68, 1H, d, *J* = 7.1 Hz, H-6; δ_H_ 4.81, 1H, m, H-8) detected in the ^1^H NMR spectrum (Table 1). The characteristic olefinic proton (δ_H_ 5.49, 1H, sept, *J* = 1.4 Hz, H-17) coupled with two vinyl methyl protons (δ_H_ 1.88, 3H, d, *J* = 1.4 Hz, H-19; δ_H_ 2.12, 3H, d, *J* = 1.4 Hz, H-20) suggested a senecioic acid side chain [8]. The low-field shifted chemical shift value caused by the deshielding effect of the carboxyl group from H-8 (δ_H_ 3.89) in crossoseamine B to H-8 (δ_H_ 4.81) in **1** indicated the attachment of a senecioic acid moiety at C-8 (δ_C_ 72.7). The HMBC correlations (Figure 2) from H-14 (δ_H_ 1.21) to C-10 (δ_C_ 46.8) and C-1 (δ_C_ 216.4); from H-2 (δ_H_ 2.34 and 2.60) to C-1 and C-3 (δ_C_ 30.3); from H-3 (δ_H_ 2.37 and 2.75) to C-1, C-4 and C-5 (δ_C_ 141.1) indicated the position of carbonyl group at C-1. The planar structure of **1** was also analyzed by COSY (Figure 2) correlations, such as the spin-spin coupling network from H-6 to H-9, and finally confirmed, as shown in Figure 1.

**Figure 3:**
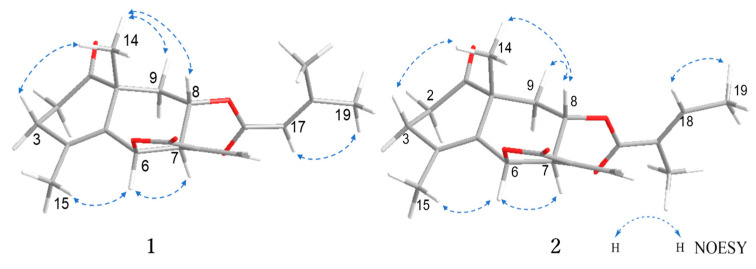
Key NOESY correlations of **1** and **2**.

The relative configuration of **1** was deduced by analysis of the 1D NMR spectrum (especially coupling constants and chemical shifts) and NOESY data (Figure 3). The following differences were observed between crossoseamine B and compound **1**. First, a smaller coupling constant (*J*_H-6/H-7_ = 7.1 Hz) in compound **1** was detected instead of a large one (*J*_H-6/H-7_ = 11.4 Hz) in crossoseamine B. Second, the chemical shifts of C-4 (δ_C_ 128.1) and C-5 (δ_C_ 141.1) in compound **1** were significantly different from C-4 (δ_C_ 126.1) and C-5 (δ_C_ 128.6) in crossoseamine B. Finally, the long-range coupling between H-6 and vinyl methyl group H_3_-15, which was detected in crossoseamine B, disappeared in compound **1**. According to the above differences, compound **1** was deduced to have the opposite stereochemistry at C-6 to crossoseamine B. Therefore, NOESY analysis was conducted to confirm it. As a result, the correlations from H-6 to H-15 and H-7; from H-14 to H-3β, H-9β and H-8 were observed, which supported the above hypothesis, and other NOESY correlations indicated the relative stereochemistry as shown in Figure 3.

**Figure 4:**
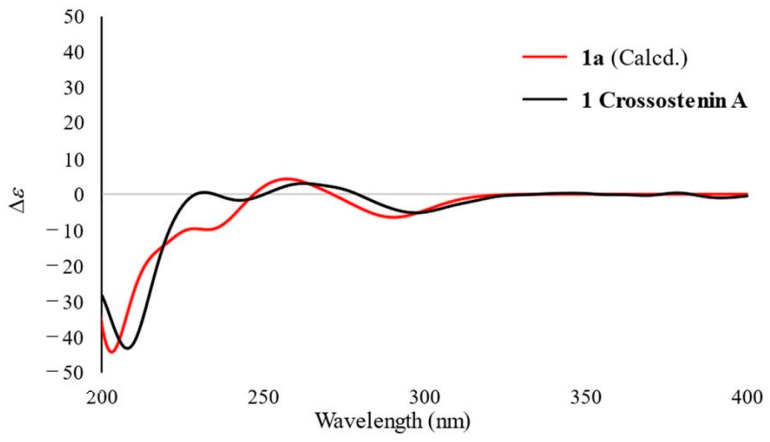
Experimental and calculated ECD spectra of **1** and **1a** in CH_3_CN.

Finally, the absolute configuration of **1** was determined by the application of the octant rule and ECD calculations. The n→π* absorption around 280–300 nm for the carbonyl group (C-1) showed a negative sign (Figure 4), which indicated the absolute stereochemistry as shown in Figure 3 by the application of the octant rule [9]. Additionally, the experimental CD and UV curves were matched to the calculated spectra of **1a** (deacyl part of **1** at C-8 to remove the unstable part for accurate calculation) (Figure 4). Considering the above results, the absolute configuration of **1** was concluded to be 6*R*, 7*R*, 8*S*, and 10*R* and designated as crossostenin A (**1**).

#### 2.1.2. Structure of Crossostenin B (**2**)

Crossostenin B (**2**) was isolated as a white amorphous powder with a molecular formula determined as C_20_H_24_O_5_ from the HR-ESI-MS data, which displayed a positive ion [M + Na]^+^ at *m*/*z* 367.1520 (calculated for C_20_H_24_O_5_Na: 367.1516). In the ^13^C NMR spectrum (Table 1), the carbon signals for the senecioic moiety in **1** were changed to δ_C_ 168.6 (C-16), 127.8 (C-17), 139.5 (C-18), 14.5 (C-19) and 12.2 (C-20), which indicated the substitution of tiglic acid moiety in **2** [10]. The multiplicity of the olefinic proton (δ_H_ 6.67, 1H, qq, *J* = 7.0, 1.4 Hz, H-18) and the vinyl methyl protons (δ_H_ 1.77, 3H, dq, *J* = 7.0, 1.4 Hz, H-19; δ_H_ 1.74, 3H, quint-like, *J* = 1.4 Hz, H-20), together with the COSY correlations from H-18 (δ_H_ 6.67) to H_3_-19 (δ_H_ 1.77) and the HMBC correlations from H-20 (δ_H_ 1.74) to C-16 (δ_C_ 168.6), 17 (δ_C_ 127.8), and 18 (δ_C_ 139.5) also validated the existence of the tiglic acid (Figure 2). The low-field shifted chemical shift value of H-8 (δ_H_ 4.83) confirmed the connection between tiglic acid and *ψ*-santonin skeleton at C-8. The relative configuration was deduced as the same as **1** by chemical shift similarity, coupling constant-based configuration analysis, and NOESY correlations (Figure 3). Specifically, NOESY correlations from H-6 to H-7 and H-15 from H-8 to H-14 and H-9β revealed the same relative configurations between **1** and **2**. Based on these spectroscopic data and similar Cotton effects (Figure S20) with compound **1**, the structure of crossostenin B (**2**) was ultimately established, as shown in Figure 1.

#### 2.1.3. Structure of Crossostenin C (**3**)

**Figure 5:**
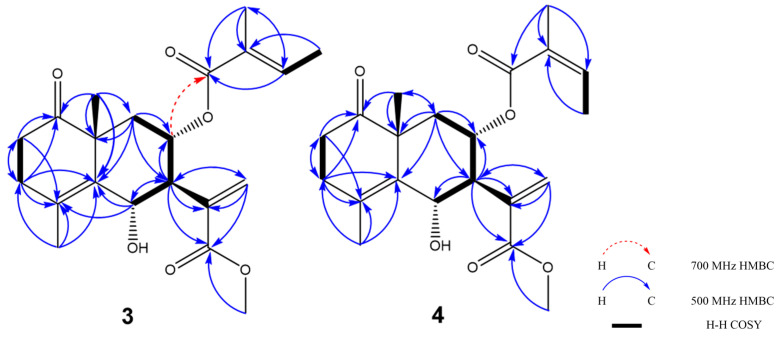
^1^H-^1^H COSY and HMBC correlations of **3** and **4**.

Crossostenin C (**3**) was obtained as a white amorphous powder. The HR-ESI-MS data showed a positive ion [M + Na]^+^ at 399.1782 (calculated for C_21_H_28_O_6_Na: 399.1778), which revealed the molecular formula of **3** was C_21_H_28_O_6_. The ^13^C NMR (Table 1) spectrum displayed three carbonyl groups (δ_C_ 216.0, 168.8 and 168.5) and three double bonds (δ_C_ 129.3, 129.8, 131.2, 135.0, 138.9 and 140.1), which provided six degrees of unsaturation. Considering the existence of a methoxy carbon (δ_C_ 52.4) in ^13^C NMR and the remaining two indices of hydrogen deficiency, the skeleton of compound **3** should be a non-lactone-type sesquiterpene structure, unlike compounds **1** and **2**. Further detailed analysis of the ^13^C NMR spectrum also revealed the presence of a tiglic acid moiety. The HMBC correlations (Figure 5) from methoxy protons (δ_H_ 3.75) to C-12 (δ_C_ 168.5) confirmed the linkage of the methoxy group and the deshielded H-8 (δ_H_ 5.44) chemical shift and the HMBC correlation from H-8 to C-16 detected by 700 MHz NMR with cryoprobe (Figure S27) revealed the connection of the tiglic acid at C-8. This HMBC correlation from H-8 to C-16 was only detected using 700 MHz NMR with a cryoprobe, not by a standard inverse probe, which is likely because of the weakening of the signal intensity by the multiple spin-spin coupling, an instability of conformation around C-8 methylene by flip-flop movement, and rotation of acyl function. The success of the detection also depended on the isolation amount (**3**, 4.7 mg), which is higher than compounds **1** (1.1 mg) and **2** (1.4 mg). Finally, the COSY coupling system (Figure 5) from H-6 (δ_H_ 4.90) to H-9 (δ_H_ 1.51 and 2.15) validated the planar structure of **3** as shown.

**Figure 6:**
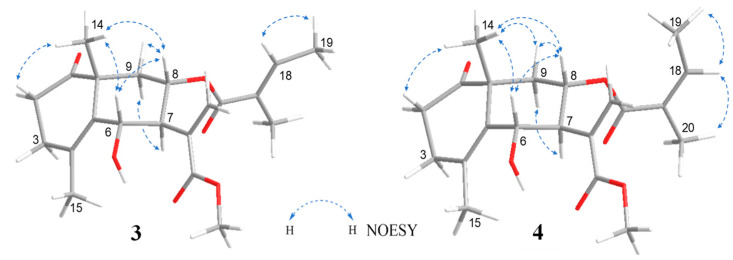
Key NOESY correlations of **3** and **4**.

The relative configuration of compound **3** was determined by the coupling constant and NOESY correlations (Figure 6). A large coupling constant between H-6 and H-7 (*J*_H-6/H-7_ = 10.1 Hz) suggested that the stereochemistry of C-6 was different from compounds **1** and **2**. In the NOESY spectrum, the correlations from H-6 to H-14 and H-8; from H-8 to H-9β and H-14; and from H-7 to H-9α were detected and consistent with the conclusion from coupling constant analysis.

**Figure 7:**
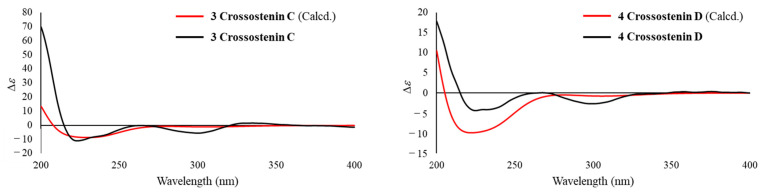
Experimental and calculated ECD spectra of **3** and **4** in CH_3_CN.

The absolute configuration of **3** was concluded by the application of the octant rule and the calculation of the ECD spectrum (Figure 7). The characteristic negative Cotton effect at around 220 nm and 300 nm was detected both in ECD and experimental CD spectra, which led to the assignment of the absolute configuration of **3** as 6*S*, 7*S*, 8*S*, and 10*R*, and named crossostenin C (**3**).

#### 2.1.4. Structure of Crossostenin D (**4**)

Crossostenin D (**4**) was obtained as a white amorphous powder with the same molecular formula as **3**, which was established by HR-ESI-MS in positive ion mode. A different side chain, instead of acetic acid, senecioic acid, and tiglic acid observed in compounds **1**–**3**, was detected in the ^13^C NMR spectrum, which showed a carbonyl group (δ_C_ 168.8), a double bond (δ_C_ 129.3 and 138.8) and two vinyl methyl groups (δ_C_ 16.1 and 20.8), and then ascribed to the angelic acid [10]. The NOESY analysis (Figure 6) and ECD calculation (Figure 7) confirmed the relative and absolute configurations, respectively. Hence, the structure of **4** was determined, as shown in Figure 1.

#### 2.1.5. Structure of Crossoseamine C (**5**)

**Figure 8:**
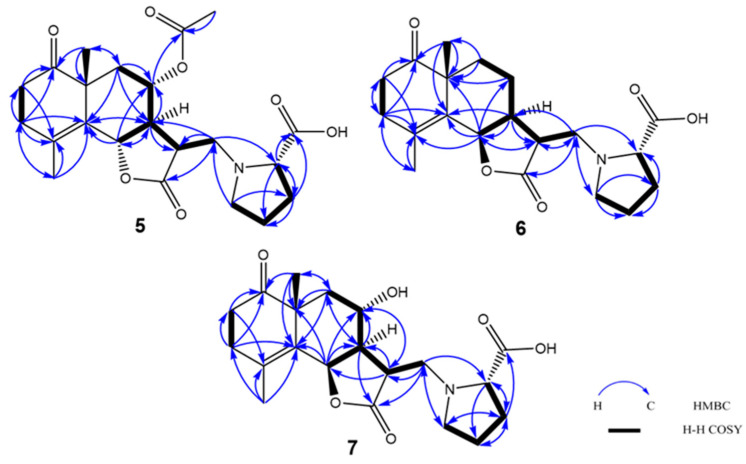
^1^H-^1^H COSY and HMBC correlations of **5**–**7**.

Crossoseamine C (**5**) was purified as a white amorphous powder and found to have a nitrogen function based on TLC examination using Dragendorff’s reagent. The molecular formula was determined as C_22_H_29_O_7_N by HR-ESI-MS data, which displayed a positive ion [M + Na]^+^ at *m*/*z* 442.1839 (calculated for C_22_H_29_O_7_NNa: 442.1836). The ^13^C NMR spectrum (Table 2) showed four carbonyl groups (δ_C_ 211.7, 176.7, 174.1, and 169.9) and two quaternary olefinic carbons (δ_C_ 126.6 and 127.9), which suggested that the remaining four indices of hydrogen deficiency belonged to four rings. In addition, five methine carbons (δ_C_ 77.5, 70.4, 66.9, 49.9, and 44.0) and three methyl groups (δ_C_ 24.4, 20.9, and 19.2) were also detected in the ^13^C NMR spectrum. The 1D NMR and HSQC spectra displayed an oxymethine proton signal at δ_H_ 4.97 (1H, dq, *J* = 11.2, 1.3 Hz, H-6) coupled with the vinyl methyl group at δ_H_ 1.89 (H_3_-15). The COSY correlations (Figure 8) from H-2′ to H-3′, H-3′ to H-4′, H-4′ to H-5′, together with the characteristic ^13^C signals at δ_C_ 174.1 (C-6′), 66.9 (C-2′), 29.2 (C-3′), 23.7 (C-4′) and 51.7 (C-5′) revealed a proline moiety [11]. Based on the chemical shifts and correlations mentioned above, the structure of compound **5** was assumed to be an acetoxy derivative of the reported compound crossoseamine B.

The HMBC correlations (Figure 8) from H-13 (δ_H_ 2.64 and 3.05) to C-2′, C-11 and C-12, from H-5′ (δ_H_ 3.02) to C-13 and C-3′ revealed the attachment of proline moiety at C-13 with N. Correlations from H-14 (δ_H_ 1.31) to C-1, C-10 and C-9; from H-2 to C-1, C-4; from H-3 to C-2, C-1, C-4, C-5 and C-15 confirmed the position of the carbonyl group at C-1. The attachment of the acetyl group was supported by the correlations from H-8 (δ_H_ 5.12) to C-16 (δ_C_ 169.9).

**Figure 9:**
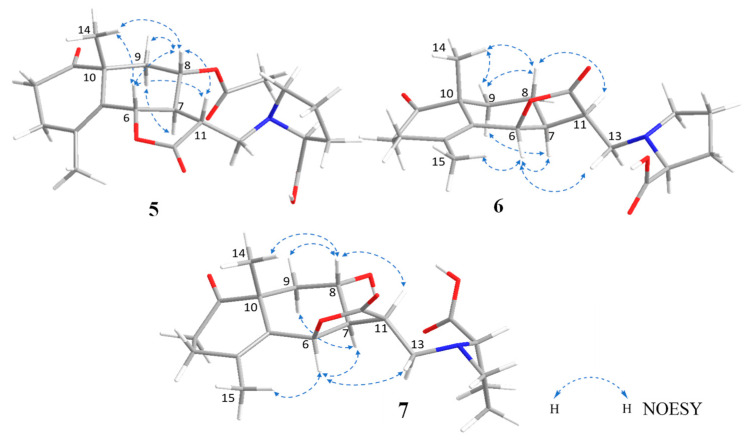
Key NOESY correlations of **5**–**7**.

**Figure 10:**
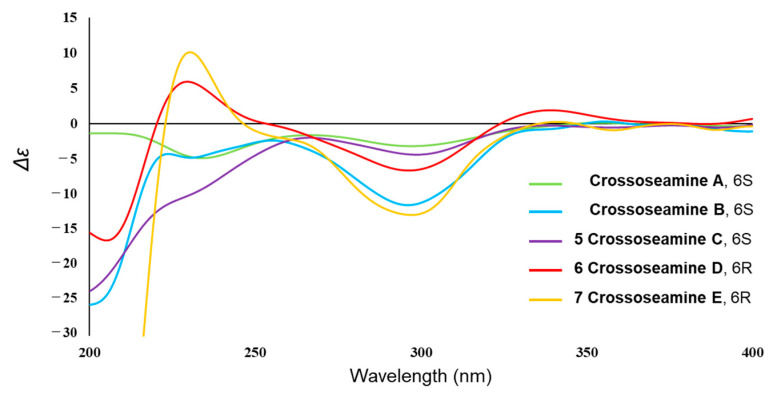
Experimental CD spectra of Crossoseamine A–E in CH_3_CN.

To determine the absolute configuration of compound **5**, NOESY, CD spectra measurement, and acid hydrolysis were conducted. The NOESY correlations between H-14 and H-6/H-8, between H-6 and H-8/H-11, between H-8 and H-9β/H-11, between H-7 and H-9α suggested the same relative configuration to crossoseamine B (Figure 9). Acid hydrolysis of **5** with 1% HCl was conducted as in previous reports and liberated L-proline by HPLC analysis with an optical rotation detector [12]. In the CD spectrum (Figure 10), the Cotton effects of **5** (∆ε (nm): −5.37 (231), −5.06 (299), CH_3_CN) were similar to crossoseamine B (∆ε (nm): −5.19 (230), −5.67 (296), CH_3_CN), which indicated the absolute configuration should be 6*S*, 7*S*, 10*R*, 11*R*, and 2′*S*. Accordingly, the structure of crossoseamine C (**5**) was determined, as shown in Figure 1.

#### 2.1.6. Structure of Crossoseamine D (**6**)

Crossoseamine D (**6**) was obtained as a white amorphous powder with the same molecular formula (C_20_H_27_O_5_N, established by HR-ESI-MS positive peaks [M + Na]^+^ at *m*/*z* 384.1780, calculated for C_20_H_27_O_5_NNa: 384.1781) to crossoseamine A [7]. The ^13^C NMR spectrum (Table 2) showed three carbonyl groups (δ_C_ 213.6, 177.9, and 174.3), one double bond (δ_C_ 127.2 and 139.1, both are quaternary carbons), and two methyl groups (δ_C_ 23.9 and 19.2). The ^1^H NMR spectrum displayed an oxygenated methine proton with a small coupling constant (δ_H_ 5.56, 1H, d, *J* = 5.6 Hz, H-6), which indicated *cis* configuration to H-7. Furthermore, ^1^H and ^13^C NMR data of compound **6** suggested a similar structure to crossoseamine A with a difference in the configuration at C-6. The HMBC and COSY correlations (Figure 8) were also consistent with this assumption. In the PS-NOESY spectrum (Figure 9), key correlations from H-6 to H-15/H-7/H-13a, from H-7 to H-9α suggested the same direction of H-6 and H-7 as α; correlations from H-14 to H-9β/H-8, from H-9β to H-8, from H-8 to H-11 revealed a β configuration of H-14, H-8 and H-11. The absolute configuration was analyzed by comparing the CD spectra (Figure 10). The Cotton effects of compound **6** were detected at 229 nm (+5.41) and 298 nm (−5.46) in CH_3_CN, which showed an opposite sign at 229 nm to the 6*S* derivatives, crossoseamine C (**5**), A, and B, which indicated that compound **6** has a 6*R* configuration. Additionally, the negative Cotton effects at 298 nm (−5.46) belonging to the n-π* transition of the 1-ketone functional group also supported the 6*R* configuration by applying the octant rule. According to the above results, the absolute configuration of **6** was determined to be 6*R*, 7*S*, 10*R*, 11*R*, and 2′*S,* as shown in Figure 1.

#### 2.1.7. Structure of Crossoseamine E (**7**)

Crossoseamine E (**7**) was purified as a white amorphous powder with a molecular formula C_20_H_27_O_6_N determined by HR-ESI-MS, which revealed a protonated molecule [M + H]^+^ at *m*/*z* 378.1912, calculated for C_20_H_28_O_6_N: 378.1911. Comparing the ^13^C NMR with compound **6**, an additional oxymethine carbon at δ_C_ 65.2 was observed, which suggested the presence of a hydroxyl group at C-8 like crossoseamine B and C (**5**). The COSY spin-spin coupling network from H-6 to H-9 and H-13, along with the HMBC correlations (Figure 8), confirmed the planar structure of **7**. The small coupling constant (*J*_H-6/H-7_ = 5.8 Hz) and the NOESY correlations from H-8 to H-9β/H-14/H-11, from H-7 to H-6/H-9α revealed the same relative configuration as **6**. To determine the absolute configuration, CD spectrum was measured (Figure 10). The positive Cotton effect in **7** at 230 nm (+5.53) and negative Cotton effect at 297 nm (−5.51) suggested the absolute configuration of **7** as 6*R*, 7*S*, 10*R*, 11*R*, and 2′*S*.

By comparing the spectroscopic data with references, the known compounds (**8**–**17**) were determined as boscialin 4′-*O*-glucoside (**8**) [13], turpinionoside A (**9**) [14], glycerine (**10**) [15], benzyl glucoside (**11**) [16], picein (**12**) [17], adenosine (**13**) [18], apigenin 7-*O*-[α-L-rhamnopyranosyl-(1→6)-β-D-glucopyranoside] (**14**) [19], apigenin 3-*O*-[α-L-rhamnopyranosyl-(1→6)-β-D-glucopyranoside] (**15**) [20], narcissin (**16**) [21], and rutin (**17**) [22].

### 2.2. Cytotoxic Activities of the Isolated Compounds

All isolated compounds (**1**–**17**) were tested against the protozoan parasite (*Leishmania major*), and their cytotoxicity against cell lines (A549 and Vero cells) was also evaluated (Table 3). Among the seven new *ψ*-santonin derivatives (**1**–**7**), crossostenin A-D (**1**–**4**) and crossoseamine C (**5**) showed extremely strong activity against *L. major* with lower IC_50_ than the positive control (miltefosine), especially compound **1** (0.81 ± 0.26 μM) and **2** (0.96 ± 0.17 μM). On the other hand, none of the evaluated compounds showed more potent cytotoxicity than doxorubicin against the A549 and Vero cells. In addition, the comparatively high selectivity (7.4 and 8.2, respectively) of crossosrenin A (**1**) and B (**2**) against *L. major* was observed. The IC_50_ data of other compounds (**8**–**17**) was not described because of the poor activities at a maximum concentration (100 μg/mL) of our experiment.

Considering the structure–activity relationship, the lactone ring formation is important for the potent anti-*Leishmania major* activity of **1** and **2**. The hydrolysis of lactone and the introduction of proline moiety reduced the activity. However, the stereochemistry at C-6 (6S) and the presence of the acyl function at C-8 as compound **5** showed the highest activity among proline conjugates **5**–**7**.

## 3. Experiments

### 3.1. General Experimental Procedure

Column chromatography was performed on silica gel 60 (Merck, Darmstadt, Germany) and Cosmosil 75C18-OPN (Nacalai Tesque, Kyoto, Japan). TLC analysis was performed on precoated silica gel 60 F_254_ plates (Merck; 0.25 mm in thickness). HPLC was performed on an Inertsil ODS-3 column (GL Science, Tokyo, Japan; Φ = 10 mm, L = 25 cm) or Cosmosil πNAP column (Nacalai Tesque, Kyoto, Japan; Φ = 10 mm, L = 25 cm), and the eluate was monitored with refractive index detector. Proline was analyzed by HPLC on a HILIC column using a chiral detector (JASCO OR-2090 plus) (Cosmosil HILIC (Nacalai Tesque, Kyoto, Japan, CH_3_CN-H_2_O (4:1), 0.7 mL/min)).

^1^H and ^13^C NMR spectra were measured on an Avance III HD spectrometer (Bruker, Billerica, MA, USA) at 500 and 125 MHz, respectively, with the residual solvent signal as references. An Avance NEO 700 MHz spectrometer with a cryoprobe (Bruker, Billerica, MA, USA) was also used for the HMBC measurement. Positive- and negative-ion HR-ESI-MS were recorded on an LTQ Orbitrap XL spectrometer (Thermo Fisher Scientific, Waltham, MA, USA). A P-1030 spectropolarimeter (JASCO, Tokyo, Japan) was used for the measurement of specific optical rotations. IR and UV spectra were measured on FT-720 (HORIBA, Kyoto, Japan) and V-520 UV/Vis spectrophotometers (JASCO, Japan), respectively. The CD spectrum was measured on the J-720 spectropolarimeter (JASCO, Japan) and J-1500 spectropolarimeter (JASCO, Japan).

### 3.2. Plant Material

The aerial parts of *C. chinense* were collected in July 2008 in Okinawa, Japan, and a voucher specimen was deposited in the Herbarium of the Department of Pharmacognosy, Graduate School of Biomedical Sciences, Hiroshima (deposition number: 08-CC-Okinawa-0708).

### 3.3. Extraction and Isolation

Air-dried aerial parts of *C. chinense* (3.5 kg) were extracted with MeOH (3 × 10 L) at room temperature. The methanol extract was evaporated to 1.5 L and then partitioned with an equal volume of *n*-hexane to obtain an *n*-hexane soluble layer (27.7 g). The remaining layer was concentrated and resuspended in 1.5 L of water and then extracted with 1.5 L EtOAc and 1.5 L 1-BuOH successively to give EtOAc (74.3 g), 1-BuOH (30.5 g) and H_2_O (171.2 g) soluble fractions.

The 1-BuOH soluble layer (27.3 g of 30.5 g) was subjected to silica gel open column chromatography (CC) (Φ = 4, L = 33 cm) with gradient solvent system of MeOH in CHCl_3_ [CHCl_3_-MeOH (20:1, 2.0 L), CHCl_3_-MeOH (10:1, 2.0 L), CHCl_3_-MeOH (7:1, 2.0 L), CHCl_3_-MeOH (5:1, 2.0 L), CHCl_3_-MeOH (3:1, 2.0 L), CHCl_3_-MeOH (2:1, 2.0 L), CHCl_3_-MeOH (1:1, 2.0 L), and (MeOH, 2.0 L)] to obtain eight fractions [Fr. 1 (5.21 g), Fr. 2 (0.76 g), Fr. 3 (1.46 g), Fr. 4 (1.93 g), Fr. 5 (2.34 g), Fr. 6 (2.63 g), Fr. 7 (1.97 g), Fr. 8 (2.31 g)], named CC-B 1 to CC-B 8. The fraction CC-B 2 (0.76 g) was separated by reversed-phase open CC (ODS, Φ = 2.5, L = 15 cm) with gradient elution [(MeOH-H_2_O 1:4, 0.5 L), (MeOH-H_2_O 3:7, 0.5 L), (MeOH-H_2_O 2:3, 0.5 L), (MeOH-H_2_O 1:1, 0.5 L), (MeOH-H_2_O 3:2, 0.5 L), (MeOH-H_2_O 7:3, 0.5 L), (MeOH-H_2_O 4:1, 0.5 L), (MeOH-H_2_O 9:1, 0.5 L) and (MeOH, 0.5 L)]] to give nine subfractions (CC-B 2-1~2-9). Fr. CC-B 2-1 (149.2 mg) was purified by HILIC HPLC [CH_3_CN-H_2_O (19:1, *v*/*v*)] to obtain glycerine (**10**, 6.1 mg), benzyl glucoside (**11**, 1.9 mg) and picein (**12**, 1.7 mg). Fr. CC-B 2-2 was separated by ODS HPLC [acetone-H_2_O (3:17, *v*/*v*)] to acquire boscialin 4′-*O*-glucoside (**8**, 1.2 mg) and turpinionoside A (**9**, 0.9 mg). Fr. CC-B 3 (1.46 g) was subjected to ODS CC with solvent [(MeOH-H_2_O 1:9, 0.5 L), (MeOH-H_2_O 1:4, 0.5 L), (MeOH-H_2_O 3:7, 0.5 L), (MeOH-H_2_O 2:3, 0.5 L), (MeOH-H_2_O 1:1, 0.5 L), (MeOH-H_2_O 3:2, 0.5 L), (MeOH-H_2_O 7:3, 0.5 L), (MeOH-H_2_O 4:1, 0.5 L), (MeOH-H_2_O 9:1, 0.5 L) and (MeOH, 0.5 L)]] to yield ten subfractions (CC-B 3-1~3-10). The subfraction CC 3–4 (182.2 mg) was repeatedly purified with ODS HPLC [acetone-H_2_O (1:3–7:13, *v*/*v*)] to obtain apigenin 7-*O*-[α-L-rhamnopyranosyl-(1→6)-β-D-glucopyranoside] (**14**, 6.3 mg) and apigenin 3-*O*-[α-L-rhamnopyranosyl-(1→6)-β-D-glucopyranoside] (**15**, 1.1 mg). Fr. CC-B 3–5 (84.6 mg) was separated by ODS HPLC [acetone-H_2_O (1:1, *v*/*v*)] to give crossostenin A (**1**, 1.1 mg) and crossostenin B (**2**, 1.4 mg). Fr. CC-B 3-6 (63.9 mg) was purified with ODS HPLC [acetone-H_2_O (11:9, *v*/*v*)] to obtain crossostenin C (**3**, 4.7 mg) and crossostenin D (**4**, 1.5 mg). Fr. CC-B 4 (1.93 g) was subjected to ODS CC with a gradient solvent system [(MeOH-H_2_O 1:19, 0.5 L), (MeOH-H_2_O 1:9, 0.5 L), (MeOH-H_2_O 1:4, 0.5 L), (MeOH-H_2_O 3:7, 0.5 L), (MeOH-H_2_O 2:3, 0.5 L), (MeOH-H_2_O 1:1, 0.5 L), (MeOH-H_2_O 3:2, 0.5 L), (MeOH-H_2_O 7:3, 0.5 L), (MeOH-H_2_O 4:1, 0.5 L), (MeOH-H_2_O 9:1, 0.5 L) and (MeOH, 0.5 L)]] to yield eleven subfractions (CC-B 4-1~4-11). The subfraction CC-B 4-2 (78.3 mg) was separated by ODS HPLC [acetone-H_2_O (1:19, *v*/*v*)] to yield adenosine (**13**, 3.3 mg). Fr. CC-B 4-5 (151.7 mg) was purified with ODS HPLC [acetone-H_2_O (3:7, *v*/*v*)] to give rutin (**17**, 11.1 mg). Fr. CC-B 4-6 (145.7 mg) was separated as in CC-B 4-5 to afford narcissin (**16**, 3.6 mg). The fraction CC-B 5 (2.34 g) was separated as in CC-B 4 to yield eleven subfractions (CC-B 5-1~5-11). The subfraction CC-B 5-3 (113. 1 mg) was purified by π-NAP HPLC [acetone-H_2_O (3:17, *v*/*v*)] to afford crossoseamine D (**6**, 5.4 mg) and crossoseamine E (**7**, 4.5 mg). Fr. CC-B 5-4 was separated by π-NAP HPLC [acetone-H_2_O (1:4, *v*/*v*)] to give crossoseamine C (**5**, 18.7 mg).

The known compounds were identified by comparison of their physicochemical data (${\left[\alpha \right]}_{D}^{}$, IR, MS, ^1^H and ^13^C NMR) with the reported data.

Crossostenin A (**1**). White amorphous powder; ${\left[\alpha \right]}_{D}^{28}$ −31.8 (c 0.11, MeOH); UV (CH_3_CN) λ_max_ (log ε): 192 (4.38), 217sh (3.84) nm; IR (film) ν_max_ 3132, 2762, 1765, 1710, 1644, 1267, 1225, 1144 cm^−1^; CD (CH_3_CN) ∆ε (nm): +4.17 (232), −4.73 (243), +4.97 (262), −5.21 (297); ^1^H-NMR (MeOD), see Table 1; ^13^C-NMR (MeOD), see Table 1; (+)-HR-ESI-MS *m*/*z* 367.1515 [M + Na]^+^ (calculated for C_20_H_24_O_5_Na, 367.1516); (+)-ESI-MS/MS *m*/*z* 284 [M + Na–C_5_H_7_O]^+^ (0.33), 267 [M + Na–C_5_H_8_O_2_]^+^ (100), 122 [M + Na–C_15_H_17_O_3_]^+^ (0.55).

Crossostenin B (**2**). White amorphous powder; ${\left[\alpha \right]}_{D}^{28}$ −66.4 (c 0.14, MeOH); UV (CH_3_CN) λ_max_ (log ε): 192 (4.30), 212sh (4.01) nm; IR (film) ν_max_ 3367, 1767, 1709, 1510, 1267 cm^−1^; CD (CH_3_CN) ∆ε (nm):−4.86 (242), +4.84 (263), −5.37 (298); ^1^H-NMR (MeOD), see Table 1; ^13^C-NMR (MeOD), see Table 1; (+)-HR-ESI-MS *m*/*z* 367.1520 [M + Na]^+^ (calculated for C_20_H_24_O_5_Na, 367.1516); (+)-ESI-MS/MS *m*/*z* 284 [M + Na–C_5_H_7_O]^2+^ (0.22), 267 [M + Na–C_5_H_8_O_2_]^+^ (100), 122 [M + Na–C_15_H_17_O_3_]^2+^ (0.38).

Crossostenin C (**3**). White amorphous powder; ${\left[\alpha \right]}_{D}^{28}$ −24.1 (c 0.34, MeOH); UV (CH_3_CN) λ_max_ (log ε): 210sh (4.20) nm; IR (film) ν_max_ 3501, 2928, 1711, 1650, 1441, 1258, 1143 cm^−1^; CD (CH_3_CN) ∆ε (nm): −5.49 (227), +4.18 (265), −5.31 (298); ^1^H-NMR (MeOD), see Table 1; ^13^C-NMR (MeOD), see Table 1; (+)-HR-ESI-MS *m*/*z* 399.1782 [M + Na]^+^ (calculated for C_21_H_28_O_6_Na, 399.1778); (+)-ESI-MS/MS *m*/*z* 299 [M + Na–C_5_H_8_O_2_]^+^ (100), 277 [M–C_5_H_7_O_2_]^+^ (10).

Crossostenin D (**4**). White amorphous powder; ${\left[\alpha \right]}_{D}^{28}$ −5.3 (c 0.15, MeOH); UV (CH_3_CN) λ_max_ (log ε): 192 (4.35), 213sh (3.87) nm; IR (film) ν_max_ 3022, 2929, 1714, 1658, 1446, 1160 cm^−1^; CD (CH_3_CN) ∆ε (nm): −4.68 (232), +4.32 (263), −4.80 (298); ^1^H-NMR (MeOD), see Table 1; ^13^C-NMR (MeOD), see Table 1; (+)-HR-ESI-MS *m*/*z* 399.1778 [M + Na]^+^ (calculated for C_21_H_28_O_6_Na, 399.1778); (+)-ESI-MS/MS *m*/*z* 299 [M + Na–C_5_H_8_O_2_]^+^ (100), 277 [M–C_5_H_7_O_2_]^+^ (24).

Crossoseamine C (**5**). White amorphous powder; ${\left[\alpha \right]}_{D}^{28}$ −58.6 (c 0.16, MeOH); UV (CH_3_CN) λ_max_ (log ε): 192 (4.42) nm; IR (film) ν_max_ 3383, 2999, 1776, 1747, 1712, 1626, 1403 cm^−1^; CD (CH_3_CN) ∆ε (nm): −5.37 (231), −5.06 (299); ^1^H-NMR (DMSO-*d_6_*), see Table 2; ^13^C-NMR (DMSO-*d_6_*), see Table 2; (+)-HR-ESI-MS *m*/*z* 442.1839 [M + Na]^+^ (calculated for C_22_H_29_O_7_NNa, 442.1836); (+)-ESI-MS/MS *m*/*z* 382 [M + Na–C_2_H_4_O_2_ (Acetyl)]^+^ (63), 327 [M + Na–C_5_H_9_O_2_N]^+^ (81), 267 [M + Na–C_7_H_13_O_4_N]^+^ (65), 137 [M + Na–C_17_H_21_O_5_]^+^ (100).

Crossoseamine D (**6**). White amorphous powder; ${\left[\alpha \right]}_{D}^{28}$ −56.1 (c 0.83, MeOH); UV (CH_3_CN) λ_max_ (log ε): 192 (4.23) nm; IR (film) ν_max_ 3383, 2929, 1760, 1706, 1639, 1406 cm^−1^; CD (CH_3_CN) ∆ε (nm): +5.41 (229), −5.46 (298); ^1^H-NMR (DMSO-*d*_6_), see Table 2; ^13^C-NMR (DMSO-*d*_6_), see Table 2; (+)-HR-ESI-MS *m*/*z* 384.1780 [M + Na]^+^ (calculated for C_20_H_27_O_5_NNa, 384.1781); (+)-ESI-MS/MS *m*/*z* 340 [M + Na–CO_2_]^+^ (100), 269 [M + Na–C_5_H_9_O_2_N]^+^ (10), 137 [M + Na–C_15_H_19_O_3_]^+^ (25).

Crossoseamine E (**7**). White amorphous powder; ${\left[\alpha \right]}_{D}^{28}$ −131.3 (c 0.45, MeOH); UV (CH_3_CN) λ_max_ (log ε): 192 (4.31) nm; IR (film) ν_max_ 3383, 2957, 1760, 1706, 1628, 1446 cm^−1^; CD (CH_3_CN) ∆ε (nm): +5.53 (230), −5.51 (297); ^1^H-NMR (DMSO-*d_6_*), see Table 2; ^13^C-NMR (DMSO-*d_6_*), see Table 2; (+)-HR-ESI-MS *m*/*z* 378.1912 [M + H]^+^ (calculated for C_20_H_28_O_6_N, 378.1911); (+)-ESI-MS/MS *m*/*z* 360 [M + H–H_2_O]^+^ (100), 332 [M–COOH]^+^ (0.34), 128 [M–C_14_H_17_O_4_]^+^ (1).

### 3.4. Acid Hydrolysis of Compounds ***5***–***7***

Compounds **5**–**7** (0.2 mg each) were treated with 1% aqueous hydrochloric acid (HCl) (0.5 mL) at room temperature for 12 h. The reaction mixture was extracted with EtOAc to obtain the EtOAc and aqueous layers. The H_2_O layer was subjected to HPLC analysis with an optical rotation detector (OR-2090 plus; JASCO) on a HILIC column (Cosmosil HILIC, 10 × 250 mm, CH_3_CN-H_2_O (4:1, *v*/*v*), flow rate: 0.7 mL/min). The peaks from **5**–**7** were identical with an authentic standard, L-proline (*t*_R_: 30.0 min, negative optical rotation) [12].

### 3.5. Computational Calculations

Conformational analyses for **1a** were performed using Spartan’20 V1.1.2. with default conformational search program (Wavefunction, Inc., Irvine, CA, USA). Stable conformers up to 40 kcal/mol for **1a** were initially searched using the Merck molecular force field (MMFF) method. Then, the stable conformers obtained from the initial search were further optimized using the Hartree–Fock (HF)/3-21G and ωB97XD/6-31G* programs. The resulting conformers were subjected to ECD calculation, and the ECD calculations for these conformers were performed with Gaussian 16 (Revision A.03 by Gaussian) [23] on the ChemPark cloud system. The dominant conformers of **1a** capable of covering 100% (conformer 1: 83.1%; conformer 2: 16.9%) of the population according to Boltzmann’s distribution were selected. Time-dependent density functional theory calculations were conducted at the CAM-B3LYP/aug-cc-pVDZ level for these conformers. The resulting rotational strength data were converted to Gaussian curves (bandwidth sigma = 0.5 eV) to obtain the ECD spectra of each conformer, and the spectra were combined after Boltzmann weighting according to their population contributions. The wavelength of the spectra was corrected (+10 nm) based on the absorptions of about 220 nm (referring to the experimental and calculated UV spectra) to give the corresponding theoretical ECD spectrum.

The ECD spectra of **3** and **4** were calculated by conformer optimizing (B3LYP/6-31G(d,p) level using PCM in acetonitrile), ECD computation (M06-2X/6-31G(d,p) level with PCM in acetonitrile, 0.333 eV standard deviation), and the Boltzmann population average of each data.

### 3.6. Anti-Proliferative Activities

The anti-*Leishmania major* activities of the isolated compounds were evaluated using an MTT assay according to an established protocol [24]. In brief, gradient concentrations of sample solutions in dimethyl sulfoxide (DMSO) and *L. major* (1 × 10^5^ parasite/well) in 100 μL of M199 medium were added to a 96-well plate and then incubated for 72 h at 25 °C. An amount of 100 μL MTT solution was then replaced and incubated overnight. The absorbance of formazan solution in DMSO was measured using a microplate reader at 550 nm. Miltefosine was used as a positive control. The human lung cancer cell line A549 was cultured in 10% FCS-supplemented DMEM. The cytotoxicity assay of isolated compounds was also performed in 96-well plates using the MTT method. Various concentrations of samples in DMSO and A549 (5 × 10^3^ cells/well) were cultured in a CO_2_ incubator for 72 h. The medium was then replaced with 100 μL MTT solution and incubated for 1.5 h in the same condition. The viability was calculated by the absorbance of formazan at 550 nm using a microplate reader. The cytotoxicity assay against the Vero cell line was conducted as in A549, which is mentioned above. Doxorubicin was used as a positive control. The concentration gradient of each activity was essentially set as 100, 50, 25, 12.5, and 6.25 μg/mL (Figures S67–S69).

## 4. Conclusions

Due to the strong anti-proliferative activities of the previously reported compounds crossoseamine A and B, a more detailed phytochemical investigation of *C. chinense* was conducted, leading to the isolation of seven new *ψ*-santonin derivatives (**1**–**7**) in this work. All new compounds were evaluated for their cytotoxic activities against *Leishmania major* and A549. As a result, compounds **1**–**5** showed more significant activity against *L. major* than the positive control miltefosine, which was also the first time the anti-proliferative potentials of *ψ*-santonin derivatives were revealed. Moreover, the high selectivity against *L. major* of **1** and **2** was revealed by comparing the cytotoxicity to the Vero cells, which suggests the possibility of being seed compounds. However, a further and comprehensive structure–activity relationship investigation seems to be required in the future.

## Figures and Tables

**Table 1:** ^1^H (500 MHz) and ^13^C NMR (125 MHz) data for compounds **1**–**4** (in MeOD).

	Crossostenin A (1)		Crossostenin B (2)		Crossostenin C (3)		Crossostenin D (4)	
Position	^1^H	^13^C	^1^H	^13^C	^1^H	^13^C	^1^H	^13^C
1		216.4		214.9		216.0		216.0
2	2.34 (m) α 2.60 (dd, 14.5, 1.6) β	35.9	2.31 (dd, 16.6, 2.2) α 2.64 (dd, 16.6, 1.6) β	35.7	2.43 (m) α 2.64 (ddd, 12.8, 6.3, 5.5) β	36.7	2.43 (ddd, 13.4, 7.1, 6.1) α 2.65 (ddd, 13.4, 8.0, 6.1) β	36.7
3	2.37 (m) α 2.75 (m) β	30.3	2.39 (ddd, 16.1, 7.5, 2.2) α 2.79 (m) β	30.5	2.35 (m) α 2.51 (m) β	34.4	2.34 (br dd, 13.1, 8.0) α 2.45 (br d, 13.1) β	34.4
4		128.1		129.7		131.2		131.3
5		141.1		141.1		135.0		135.0
6	5.68 (d, 7.1)	77.5	5.70 (d, 7.2)	77.5	4.90 (br d, 10.1)	71.5	4.90 (m)	71.6
7	3.29 (m)	45.6	3.34 (m)	45.5	2.72 (t, 10.1)	57.9	2.69 (t, 10.2)	58.1
8	4.81 (m)	72.7	4.83 (m)	73.9	5.44 (ddd, 11.0, 10.1, 4.6)	71.8	5.49 (ddd, 11.1, 10.2, 4.7)	71.4
9	1.49 (dd, 14.5, 2.1) β 2.52 (ddd, 14.5, 6.6, 1.8) α	33.8	1.49 (dd, 14.5, 2.2) β 2.61 (ddd, 14.5, 6.4, 1.6) α	33.2	1.51 (dd, 12.7, 11.0) α 2.15 (dd, 12.7, 4.6) β	39.9	1.52 (dd, 12.7, 11.1), α 2.13 (dd, 12.7, 4.7) β	40.0
10		46.8		46.6		49.8		49.8
11		139.1		139.2		140.1		140.1
12		171.9		171.		168.5		168.4
12-OCH_3_					3.75 (s)	52.4	3.74 (s)	52.4
13	5.90 (d, 2.0) 6.34 (d, 2.0)	125.8	5.91 (d, 2.0) 6.35 (d, 2.0)	125.8	5.70 (d, 1.1) 6.26 (d, 1.1)	129.3	5.69 (d, 1.1) 6.27 (d, 1.1)	129.5
14	1.21 (s)	26.2	1.23 (s)	26.3	1.40 (s)	23.9	1.36 (s)	23.9
15	1.99 (d, 1.0)	20.0	2.00 (d, 0.9)	19.9	2.0 (br s)	20.9	2.03 (br s)	20.9
16		166.9		168.6		168.8		168.8
17	5.49 (sept, 1.4)	116.2		127.8		129.8		129.3
18		160.1	6.67 (qq, 7.0, 1.4)	139.5	6.77 (qq, 7.1, 1.3)	138.9	6.04 (qq, 7.2, 1.6)	138.8
19	1.88 (d, 1.4)	27.6	1.77 (dq, 7.0, 1.4)	14.5	1.77 (dq, 7.1, 1.1)	14.5	1.88 (dq, 7.2, 1.6)	16.1
20	2.12 (d, 1.4)	20.5	1.74 (quint-like, 1.4)	12.2	1.75 (dq, 1.3, 1.1)	12.2	1.78 (dq, 1.6, 1.6)	20.8

δ_H_ in ppm (multiplicity, *J* in Hz), δ_C_ in ppm, m—multiplet or overlapped signals.

**Table 2:** ^1^H (500 MHz) and ^13^C NMR (125 MHz) for compounds **5**–**7** (in DMSO-*d*_6_).

Position	Crossoseamine C (5)		Crossoseamine D (6)		Crossoseamine E (7)	
	^1^H	^13^C	^1^H	^13^C	^1^H	^13^C
1		211.7		213.6		213.1
2	2.35–2.44 (m) α 2.67 (m) β	34.9	2.27–2.35 (m) α 2.57–2.66 (m) β	34.9	2.27–2.36 (m) α 2.57–2.62 (m) β	34.6
3	2.35–2.44 (m)	32.5	2.27–2.35 (m) α 2.57–2.66 (m) β	30.0	2.27–2.36 (m) α 2.57–2.62 (m) β	29.7
4		126.6		127.2		126.8
5		127.9		139.1		138.7
6	4.97 (dq, 11.2, 1.3)	77.5	5.56 (d, 5.6)	76.9	5.67 (d, 5.8)	78.2
7	2.42 (t, 11.2)	49.9	2.47 (dd-like, 8.8, 5.6)	49.2	2.15 (dd, 8.9, 5.8)	47.6
8	5.12 (td, 11.2, 4.4)	70.4	1.35 (m) α 1.56 (m) β	22.8	3.54 (ddd, 10.8, 8.9, 3.3)	65.2
9	1.38 (dd, 12.9, 11.2) α 1.99 (dd, 12.9, 4.4) β	40.0	1.47–1.55 (m)	30.4	1.46 (dd, 13.2, 10.8) α 1.75 (m) β	39.4
10		46.9		45.3		45.8
11	2.95 (ddd, 11.2, 5.9, 3.3)	44.0	2.43 (br t, 6.6)	38.5	2.79 (br t, 6.4)	46.5
12		176.7		177.9		177.9
13	2.64 (m) 3.05 (m)	53.8	2.98 (dd, 12.9, 6.6) 3.08 (dd, 12.9, 6.6)	53.3	3.08 (dd, 12.7, 6.4) 3.02 (dd, 12.7, 6.4)	53.3
14	1.31 (s)	24.4	1.12 (s)	23.9	1.12 (s)	24.6
15	1.89 (s)	19.2	1.87 (s)	19.2	1.86 (s)	19.4
16		169.9				
17	2.02 (s)	20.9				
2′	3.38 (dd, 9.1, 3.8)	66.9	3.30 (dd, 8.9, 4.5)	66.1	3.35 (dd, 8.7, 4.4)	66.1
3′	1.83 (m) 2.05 (m)	29.2	1.82 (m) 2.06 (m)	29.0	1.76 (m) 2.03 (m)	28.6
4′	1.70 (m) 1.77 (m)	23.7	1.74 (m)	22.5	1.74 (m)	23.4
5′	3.02 (m)	51.7	2.57–2.66 (m) 3.13 (ddd, 11.0, 7.4, 3.4)	53.7	2.57–2.62 (m) 3.06 (m)	52.5
6′		174.1		174.3		174.3

δ_H_ in ppm (multiplicity, *J* in Hz), δ_C_ in ppm, m—multiplet or overlapped signals.

**Table 3:** IC_50_ of new compounds (**1**–**7**) from *C. chinense*.

Compounds	*L. major* (μM)	A549 (μM)	Vero Cell (μM)	SI_1_	SI_2_
**1**	0.81 ± 0.26	2.97 ± 0.58	5.99 ± 0.76	7.4	2.0
**2**	0.96 ± 0.17	2.30 ± 0.26	7.84 ± 0.61	8.2	3.4
**3**	3.75 ± 0.98	5.59 ± 0.72	6.20 ± 1.36	3.3	1.7
**4**	7.66 ± 2.26	17.1 ± 1.86	17.2 ± 1.36	1.7	1.1
**5**	4.72 ± 1.26	17.4 ± 1.86	11.4 ± 0.98	2.2	1.0
**6**	52.6 ± 11.6	83.9 ± 12.1	48.5 ± 4.07	2.4	0.7
**7**	30.8 ± 5.81	58.9 ± 13.6	23.8 ± 2.47	0.9	0.6
P.C.	18.1 ± 1.69	0.45 ± 0.13	3.82 ± 0.79	-	-

P.C. (positive control, *L. major* (miltefosine), A549 and Vero cells (doxorubicin)), *n* = 3. SI (selective index), SI_1_—SI to *L. major*; SI_2_—SI to A549. Compounds (**8**–**17**) were not significantly active at 100 μg/mL).

## Data Availability

Data are contained within the article or Supplementary Material.

## Supplementary Materials

The following supporting information can be downloaded at: https://www.mdpi.com/article/10.3390/molecules28248108/s1, Figures S1–S69: 1D, 2D NMR spectra, MS and CD data of compounds **1**–**7**.
