# Supplementary material for: New ψ-Santonin Derivatives from Crossostephium chinense and Their Anti-Proliferative Activities against Leishmania major and Human Cancer Cells A549
Source: Molecules. 2023 Dec 15;28(24):8108. doi: 10.3390/molecules28248108 (PMC10746127; doi:10.3390/molecules28248108)
Supplement: Supplementary file 1 [file molecules-28-08108-s001.zip › molecules-2740921-supplementary.pdf]

Supplementary Data

New *ψ*-santonin derivatives from *Crossostephium chinense* and their anti-proliferative activities against *Leishmania major* and human cancer cells A549

Zhichao Wang [1], Yoshi Yamano [1], Susumu Kawakami [2], Tohru Taniguchi [3], Gadah Abdulaziz Al-Hamoud [4], Sachiko Sugimoto [1], Hideaki Otsuka [2], Nawal M. Al Musayeib[4], Musarat Amina[4]and Katsuyoshi Matsunami [1,*]

[1] Graduate School of Biomedical & Health Sciences, Hiroshima University; 1-2-3 Kasumi, Minami-ku, Hiroshima 734-8551, Japan. zhichao96425@gmail.com (Z.W.); ssugimot@hiroshima-u.ac.jp (S.S.); yamano@hiroshima-u.ac.jp (Y.Y.); matunami@hiroshima-u.ac.jp (K.M.)

[2] Graduate School of Pharmacy, Yasuda Women's University, Hiroshima 734-8551, Japan. Otsuka-h@yasuda-u.ac.jp (H.O.); kawakami@yasuda-u.ac.jp (S.K.)

[3] Frontier Research Center for Advanced Material and Life Science, Faculty of Advanced Life Science, Hokkaido University, North 21 West 11, Sapporo 001-0021, Japan. ttaniguchi@sci.hokudai.ac.jp (T.T.)

[4] Department of Pharmacognosy, College of Pharmacy, King Saud University, Riyadh 11451, Saudi Arabia; galhamoud@ksu.edu.sa (G.A.A.-H.); nalmusayeib@ksu.edu.sa (N.M.A.M.); mamina@ksu.edu.sa (M.A.)

* Correspondence: matunami@hiroshima-u.ac.jp (K.M.); Tel.: +81-82-257-5335 (K.M.)

Contents of Supplementary material

Figure S1. Structures of known compounds from *C. chinense*
Figure S2. ^{1}H-NMR spectrum of **1** in MeOD (500 MHz)
Figure S3. ^{13}C-NMR spectrum of **1** in MeOD (125 MHz)
Figure S4. ^{13}C DEPT135 NMR spectrum of **1** in MeOD (125 MHz)
Figure S5. 1H-1H COSY spectrum of **1** in MeOD (500 MHz)
Figure S6. HSQC spectrum of **1** in MeOD (500 MHz)
Figure S7. HMBC spectrum of **1** in MeOD (500 MHz)
Figure S8. PS-NOESY spectrum of **1** in MeOD (500 MHz)
Figure S9. HR-ESI-MS spectrum of **1**
Figure S10. ESI-MS/MS spectrum of **1**
Figure S11. ^{1}H-NMR spectrum of **2** in MeOD (500 MHz)
Figure S12. ^{13}C-NMR spectrum of **2** in MeOD (125 MHz)
Figure S13. ^{13}C DEPT135 NMR spectrum of **2** in MeOD (125 MHz)
Figure S14. 1H-1H COSY spectrum of **2** in MeOD (500 MHz)
Figure S15. HSQC spectrum of **2** in MeOD (500 MHz)
Figure S16. HMBC spectrum of **2** in MeOD (500 MHz)
Figure S17. PS-NOESY spectrum of **2** in $CDCl_3$ (500 MHz)
Figure S18. HR-ESI-MS spectrum of **2**
Figure S19. ESI-MS/MS spectrum of **2**
Figure S20. Experimental CD spectrum of **2** in CH_3CN
Figure S21. ^{1}H-NMR spectrum of **3** in MeOD (500 MHz)
Figure S22. ^{13}C-NMR spectrum of **3** in MeOD (125 MHz)
Figure S23. ^{13}C DEPT135 NMR spectrum of **3** in MeOD (125 MHz)
Figure S24. 1H-1H COSY spectrum of **3** in MeOD (500 MHz)
Figure S25. HSQC spectrum of **3** in MeOD (500 MHz)
Figure S26. HMBC spectrum of **3** in MeOD (500 MHz)
Figure S27. HMBC spectrum of **3** in MeOD (700 MHz)
Figure S28. PS-NOESY spectrum of **3** in MeOD (500 MHz)
Figure S29. HR-ESI-MS spectrum of **3**
Figure S30. ESI-MS/MS spectrum of **3**
Figure S31. ^{1}H-NMR spectrum of **4** in MeOD (500 MHz)
Figure S32. ^{13}C-NMR spectrum of **4** in MeOD (125 MHz)
Figure S33. ^{13}C DEPT135 NMR spectrum of **4** in MeOD (125 MHz)
Figure S34. 1H-1H COSY spectrum of **4** in MeOD (500 MHz)
Figure S35. HSQC spectrum of **4** in MeOD (500 MHz)

Figure S36. HMBC spectrum of **4** in MeOD (500 MHz)
Figure S37. PS-NOESY spectrum of **4** in MeOD (500 MHz)
Figure S38. HR-ESI-MS spectrum of **4**
Figure S39. ESI-MS/MS spectrum of **54**
Figure S40. ^{1}H-NMR spectrum of **5** in DMSO-d_6 (500 MHz)
Figure S41. ^{13}C-NMR spectrum of **5** in DMSO-d_6 (125 MHz)
Figure S42. ^{13}C DEPT135 NMR spectrum of **5** in DMSO-d_6 (125 MHz)
Figure S43. 1H-1H COSY spectrum of **5** in DMSO-d_6 (500 MHz)
Figure S44. HSQC spectrum of **5** in DMSO-d_6 (500 MHz)
Figure S45. HMBC spectrum of **5** in DMSO-d_6 (500 MHz)
Figure S46. PS-NOESY spectrum of **5** in DMSO-d_6 (500 MHz)
Figure S47. HR-ESI-MS spectrum of **5**
Figure S48. ESI-MS/MS spectrum of **5**
Figure S49. ^{1}H-NMR spectrum of **6** in DMSO-d_6 (500 MHz)
Figure S50. ^{13}C-NMR spectrum of **6** in DMSO-d_6 (125 MHz)
Figure S51. ^{13}C DEPT135 NMR spectrum of **6** in DMSO-d_6 (125 MHz)
Figure S52. 1H-1H COSY spectrum of **6** in DMSO-d_6 (500 MHz)
Figure S53. HSQC spectrum of **6** in DMSO-d_6 (500 MHz)
Figure S54. HMBC spectrum of **6** in DMSO-d_6 (500 MHz)
Figure S55. PS-NOESY spectrum of **6** in DMSO-d_6 (500 MHz)
Figure S56. HR-ESI-MS spectrum of **6**
Figure S57. ESI-MS/MS spectrum of **6**
Figure S58. ^{1}H-NMR spectrum of **7** in DMSO-d_6 (500 MHz)
Figure S59. ^{13}C-NMR spectrum of **7** in DMSO-d_6 (125 MHz)
Figure S60. ^{13}C DEPT135 NMR spectrum of **7** in DMSO-d_6 (125 MHz)
Figure S61. 1H-1H COSY spectrum of **7** in DMSO-d_6 (500 MHz)
Figure S62. HSQC spectrum of **7** in DMSO-d_6 (500 MHz)
Figure S63. HMBC spectrum of **7** in DMSO-d_6 (500 MHz)
Figure S64. PS-NOESY spectrum of **7** in DMSO-d_6 (500 MHz)
Figure S65. HR-ESI-MS spectrum of **7**
Figure S66. ESI-MS/MS spectrum of **7**
Figure S67. Anti-L. major data of **1-7**
Figure S68. Anti-A549 data of **1-7**
Figure S69. Vero cell cytotoxicity data of **1**-**7**

Figure S1. Structures of known compounds from *C. chinense*

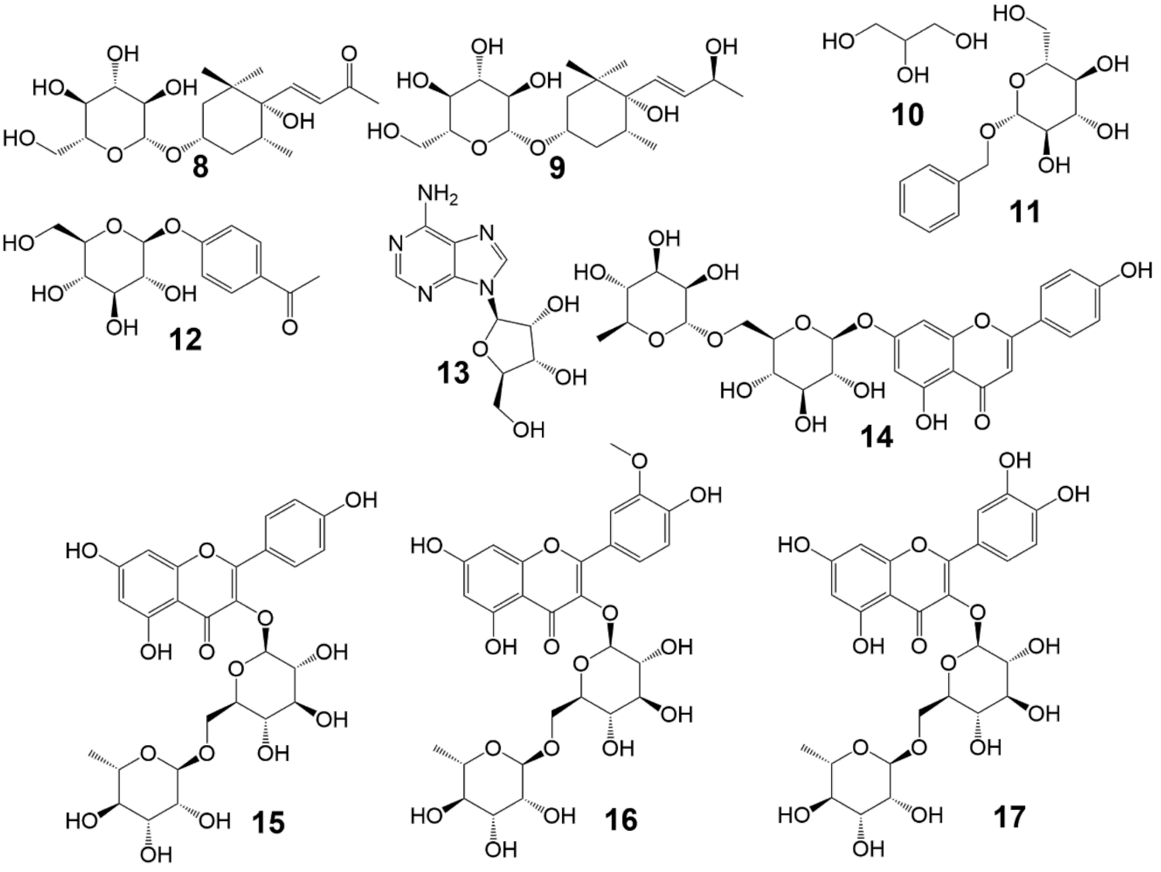

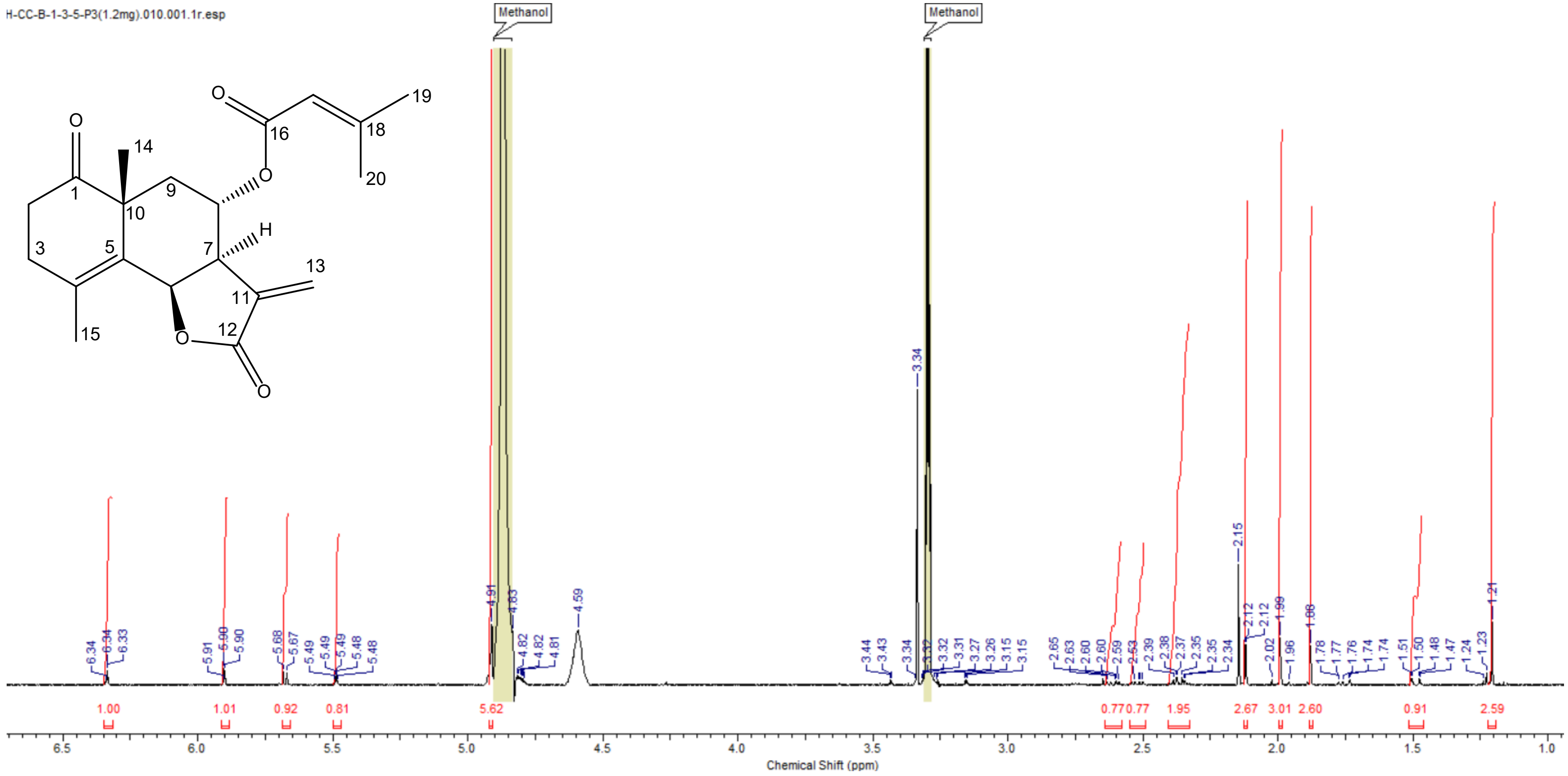

Figure S2. ^{1}H-NMR spectrum of **1** in MeOD (500 MHz)

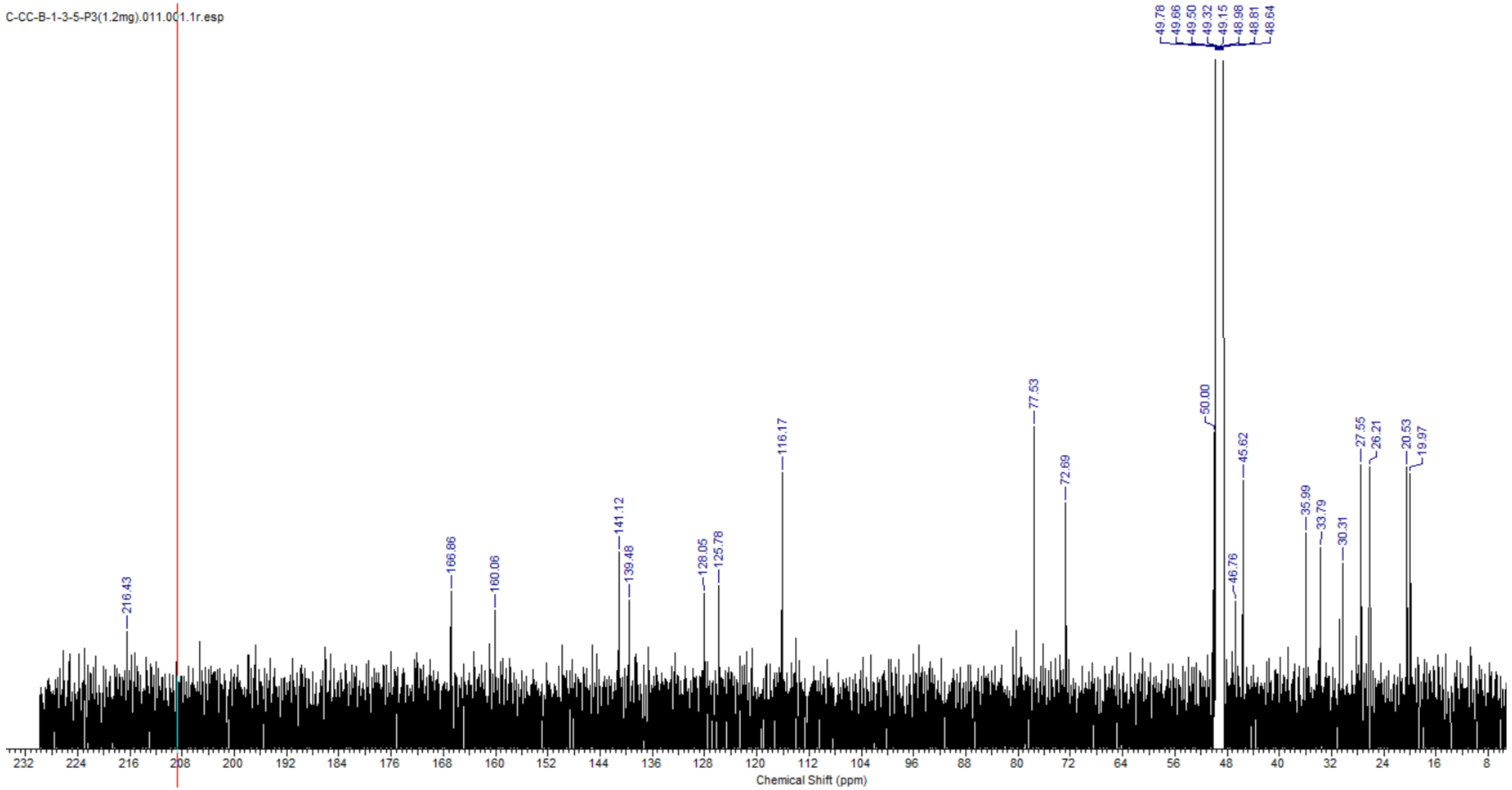

Figure S3. ^{13}C-NMR spectrum of **1** in MeOD (125 MHz)

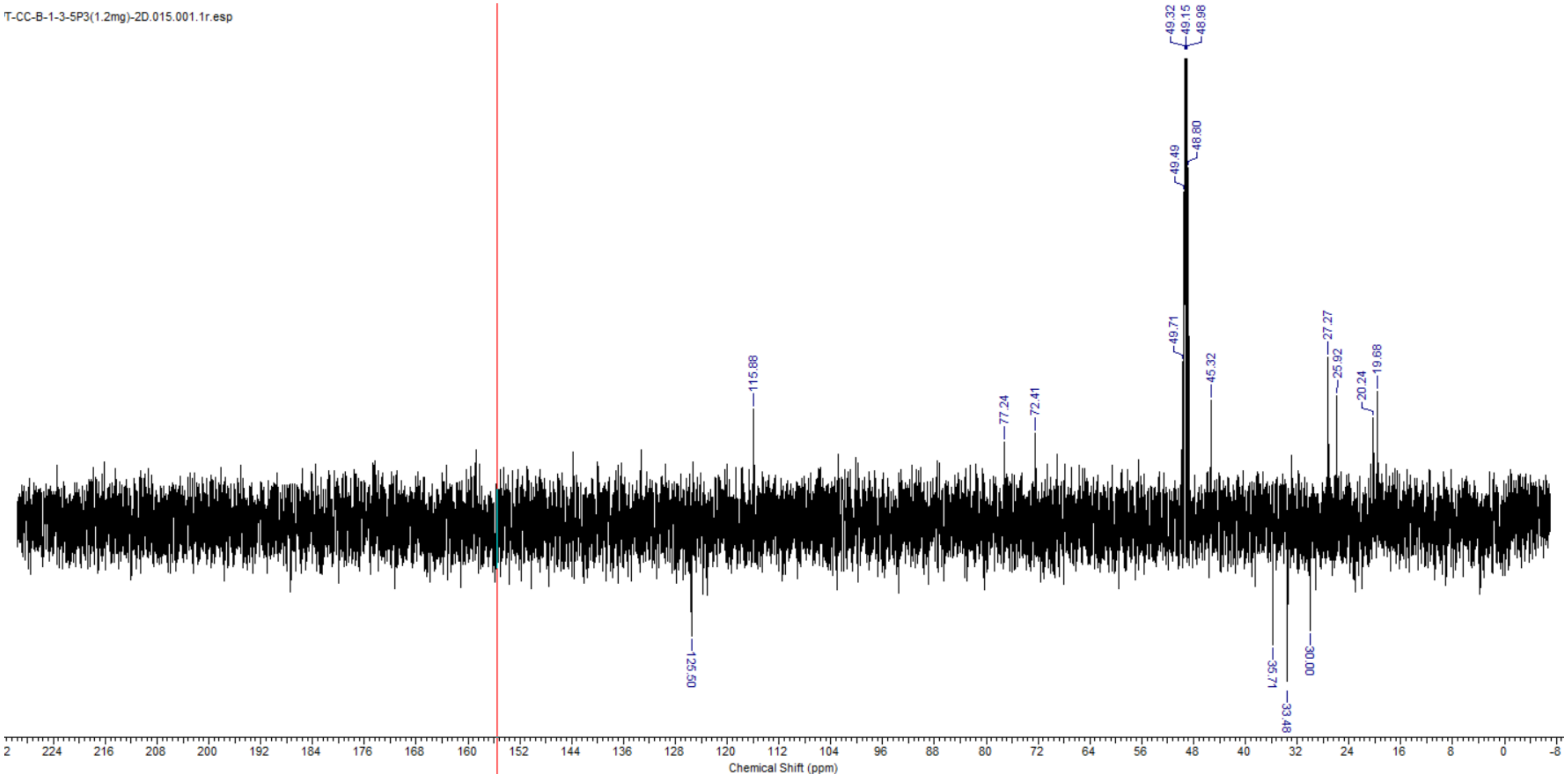

Figure S4. ^{13}C DEPT135 NMR spectrum of **1** in MeOD (125 MHz)

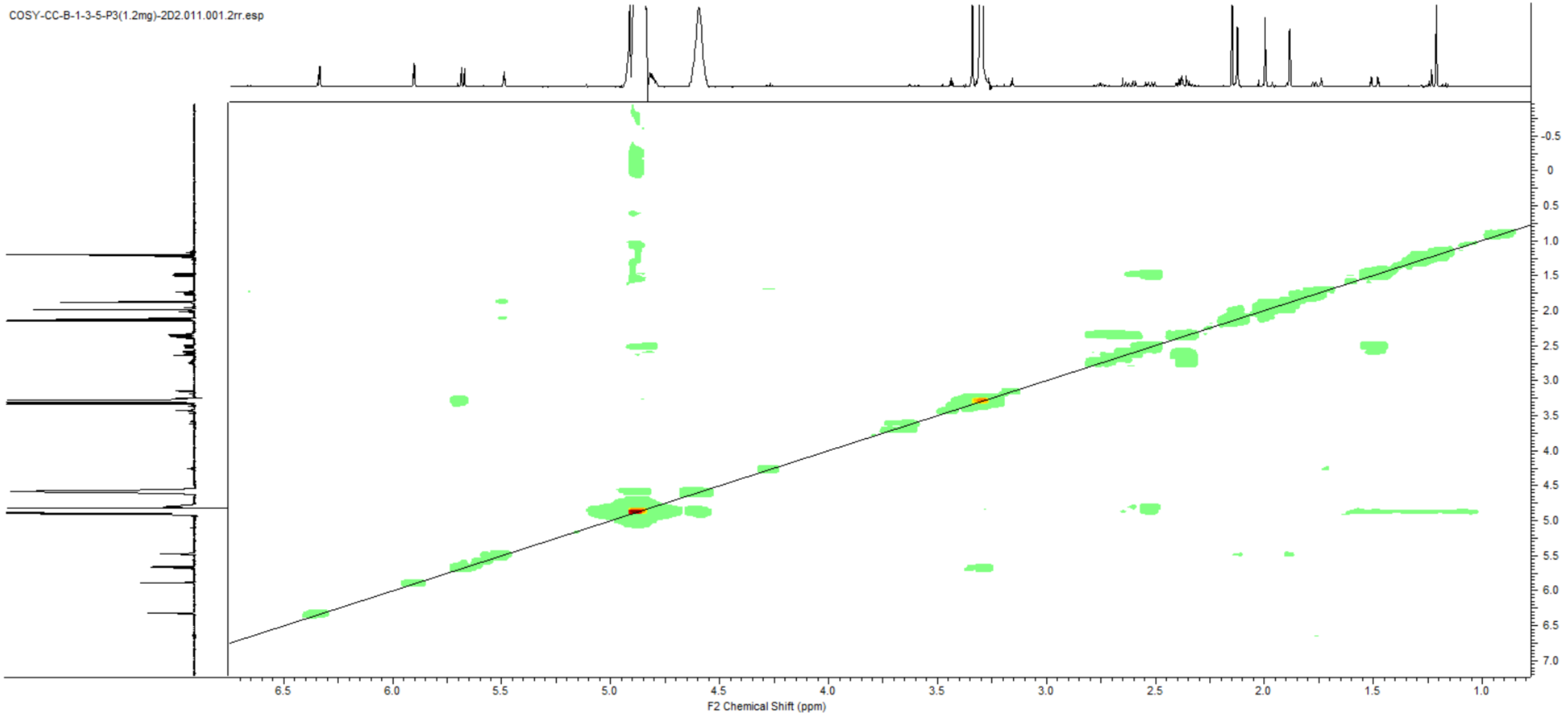

Figure S5. 1H-1H COSY spectrum of **1** in MeOD (500 MHz)

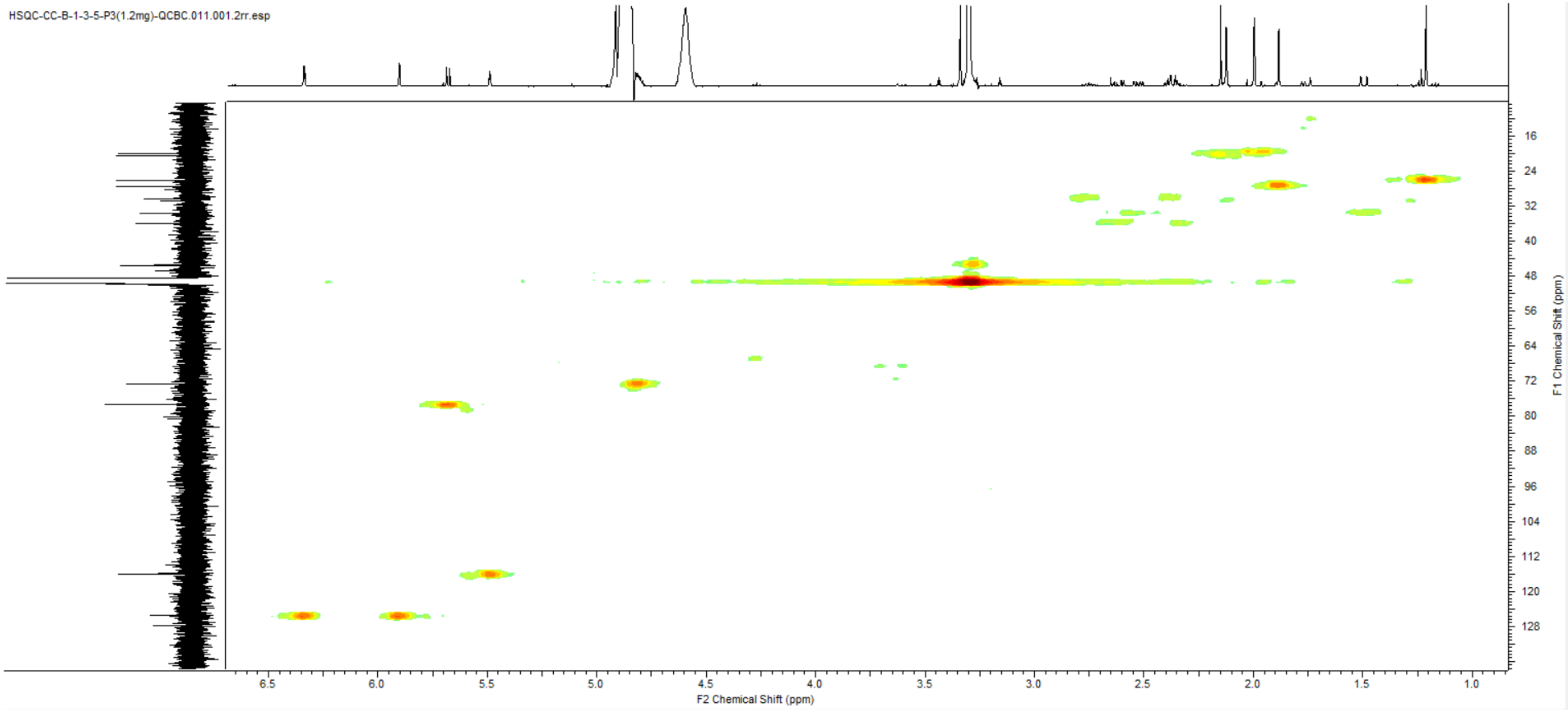

Figure S6. HSQC spectrum of **1** in MeOD (500 MHz)

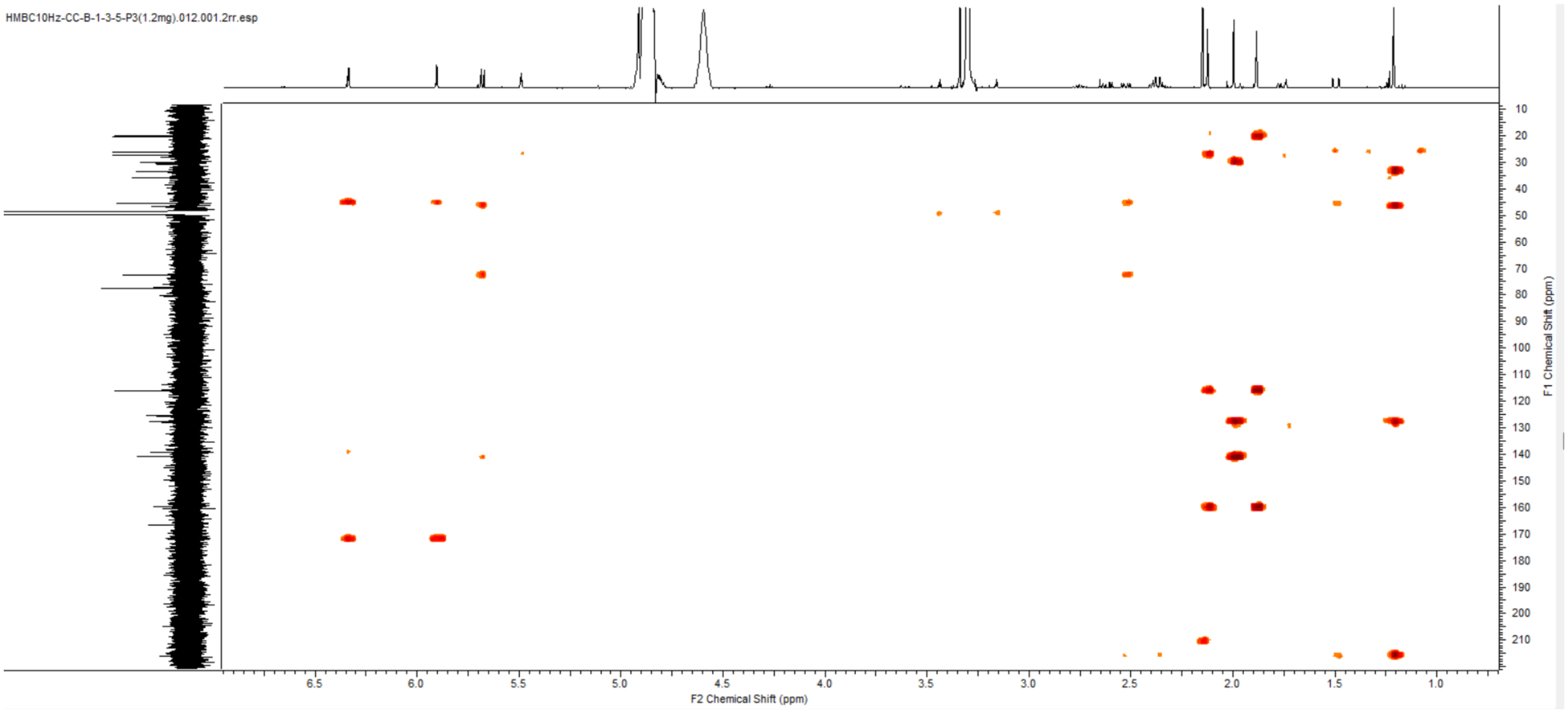

Figure S7. HMBC spectrum of **1** in MeOD (500 MHz)

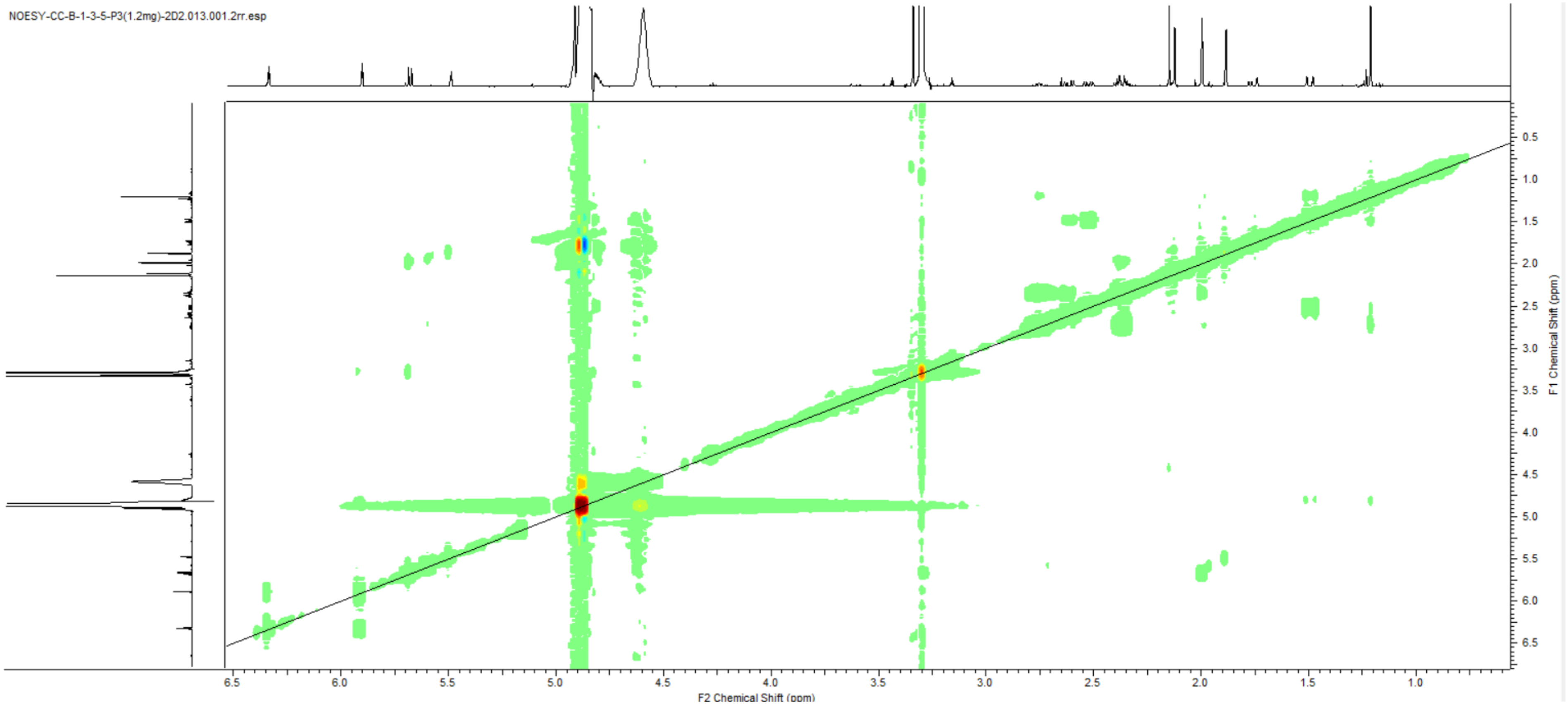

Figure S8. PS-NOESY spectrum of **1** in MeOD (500 MHz)

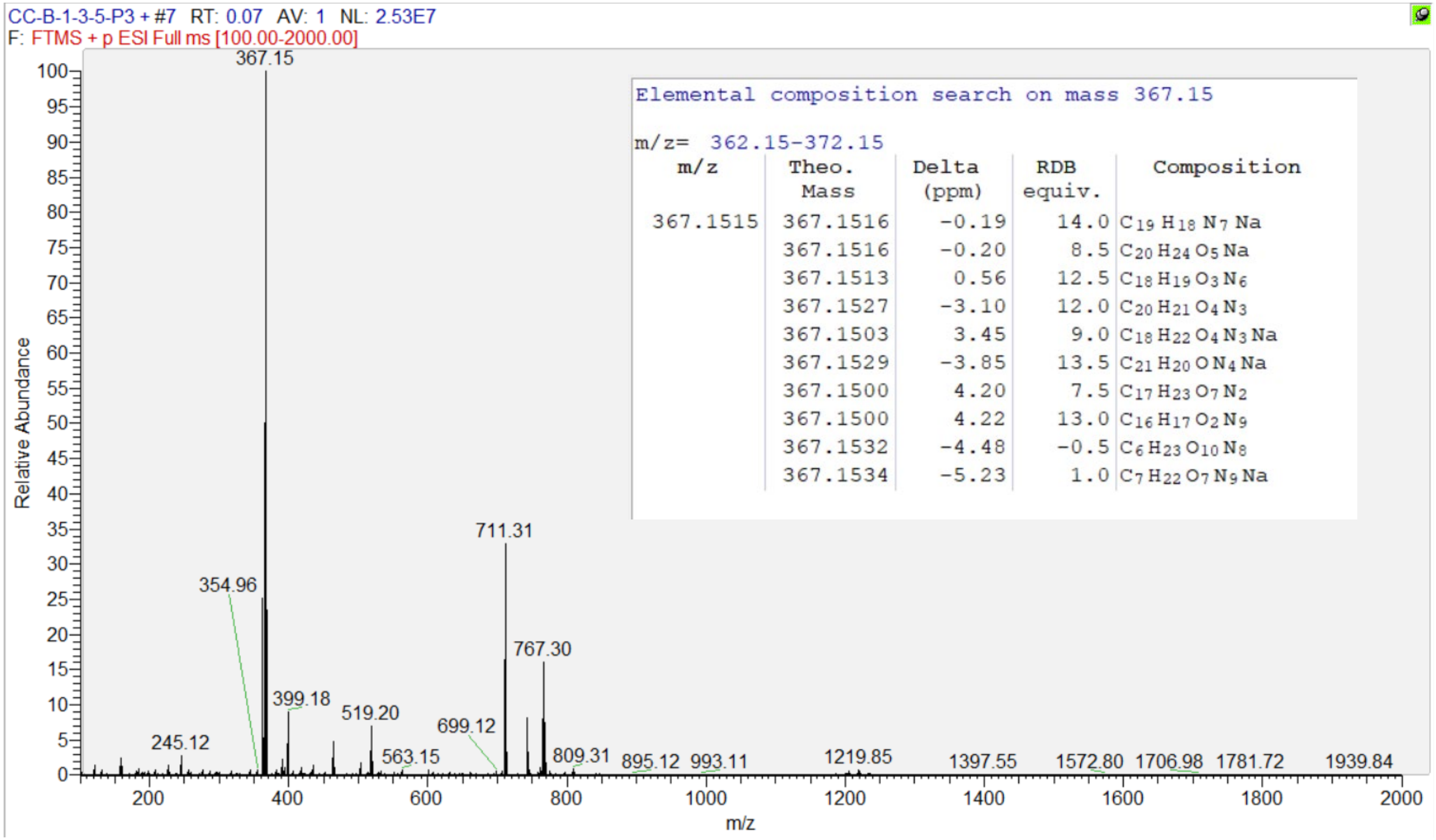

Figure S9. HR-ESI-MS spectrum of **1**

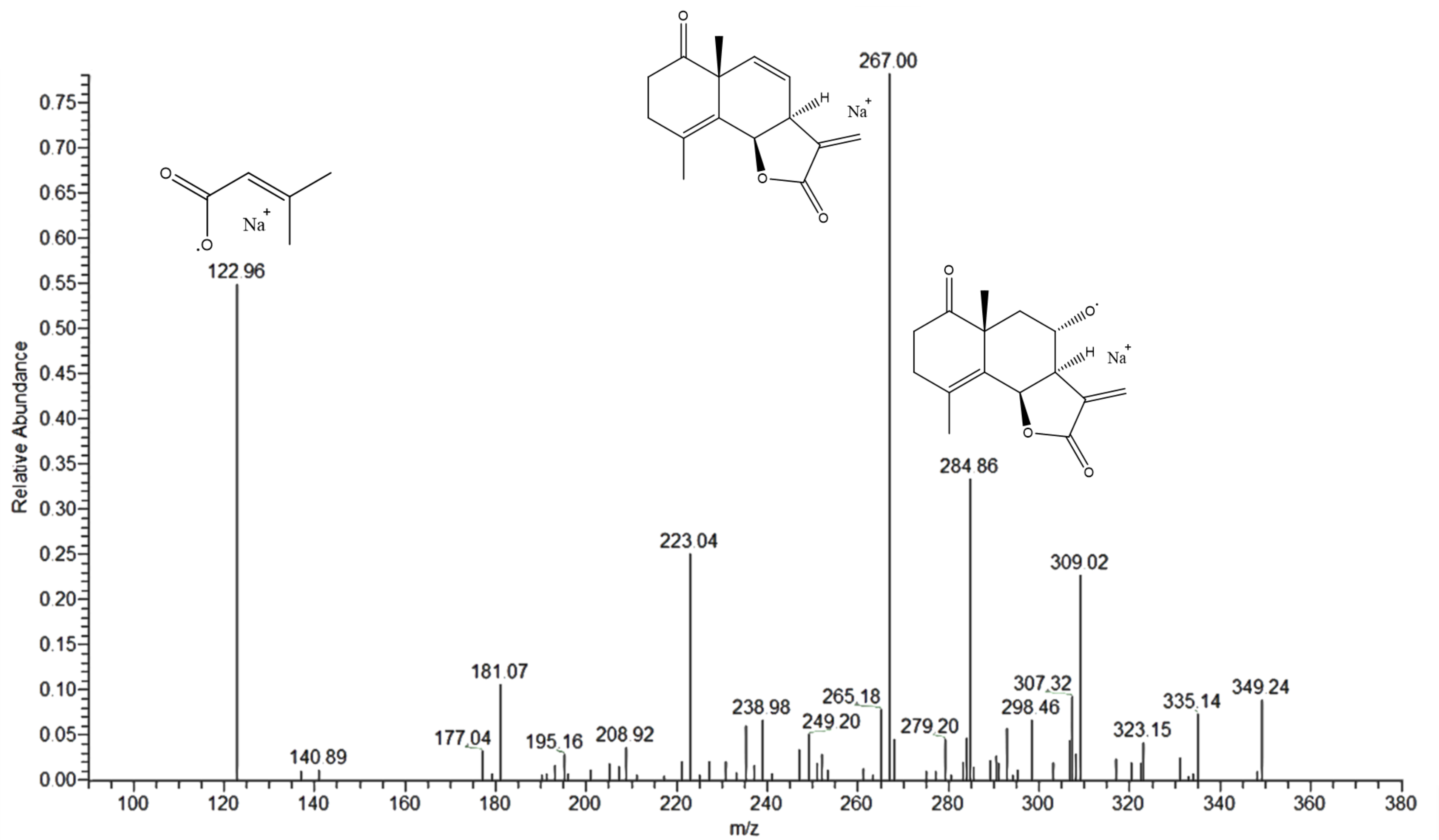

Figure S10. ESI-MS/MS spectrum of **1**

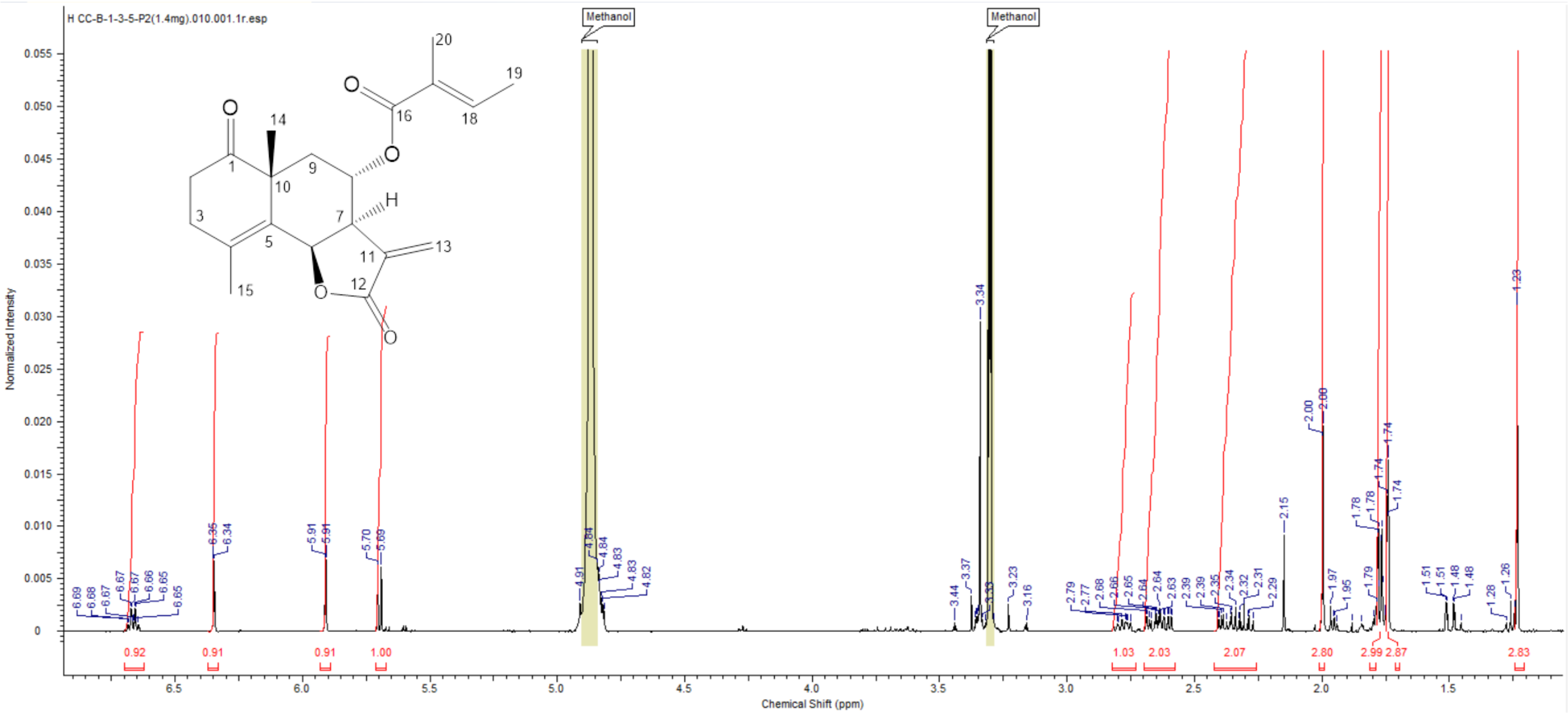

Figure S11. ^{1}H-NMR spectrum of **2** in MeOD (500 MHz)

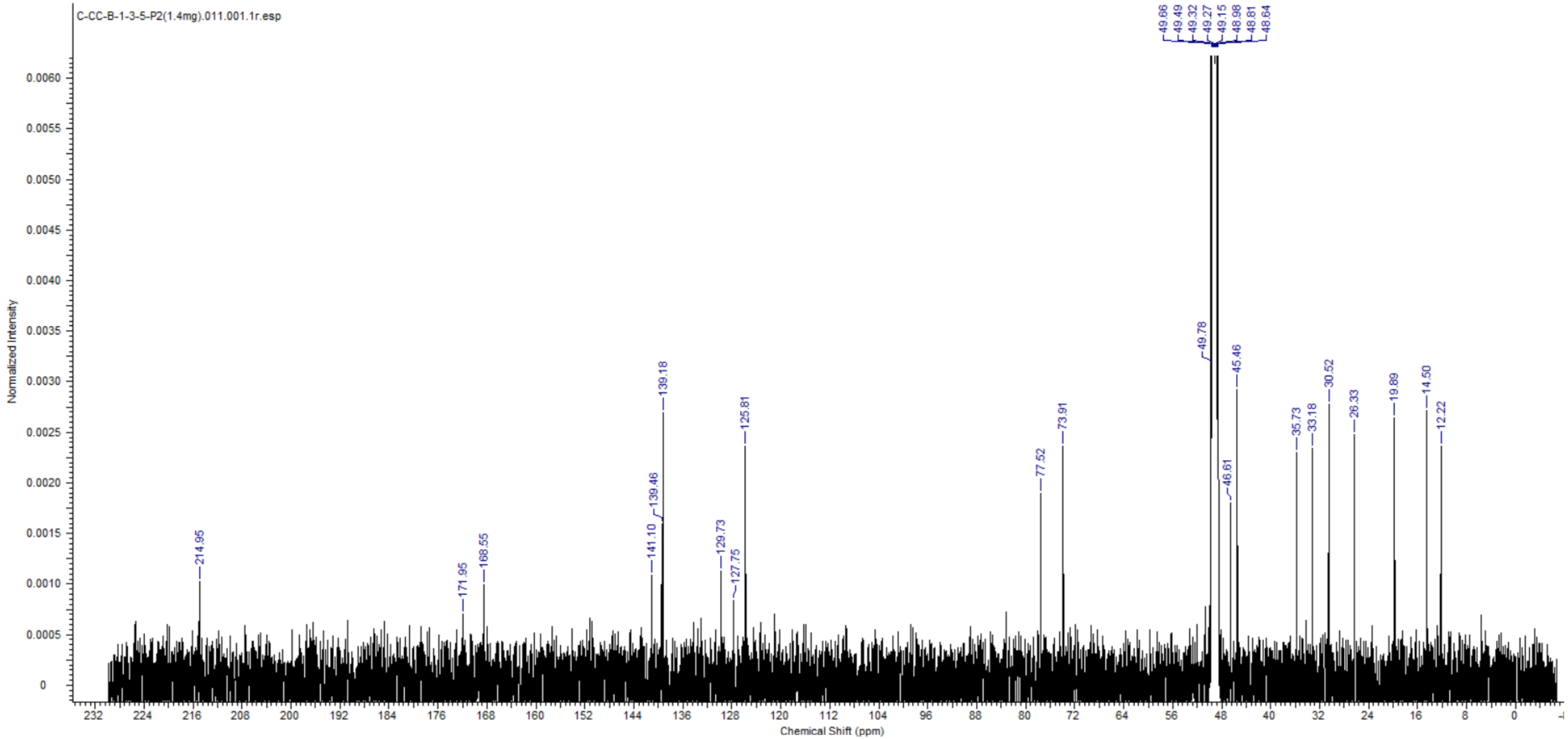

Figure S12. ^{13}C-NMR spectrum of **2** in MeOD (125 MHz)

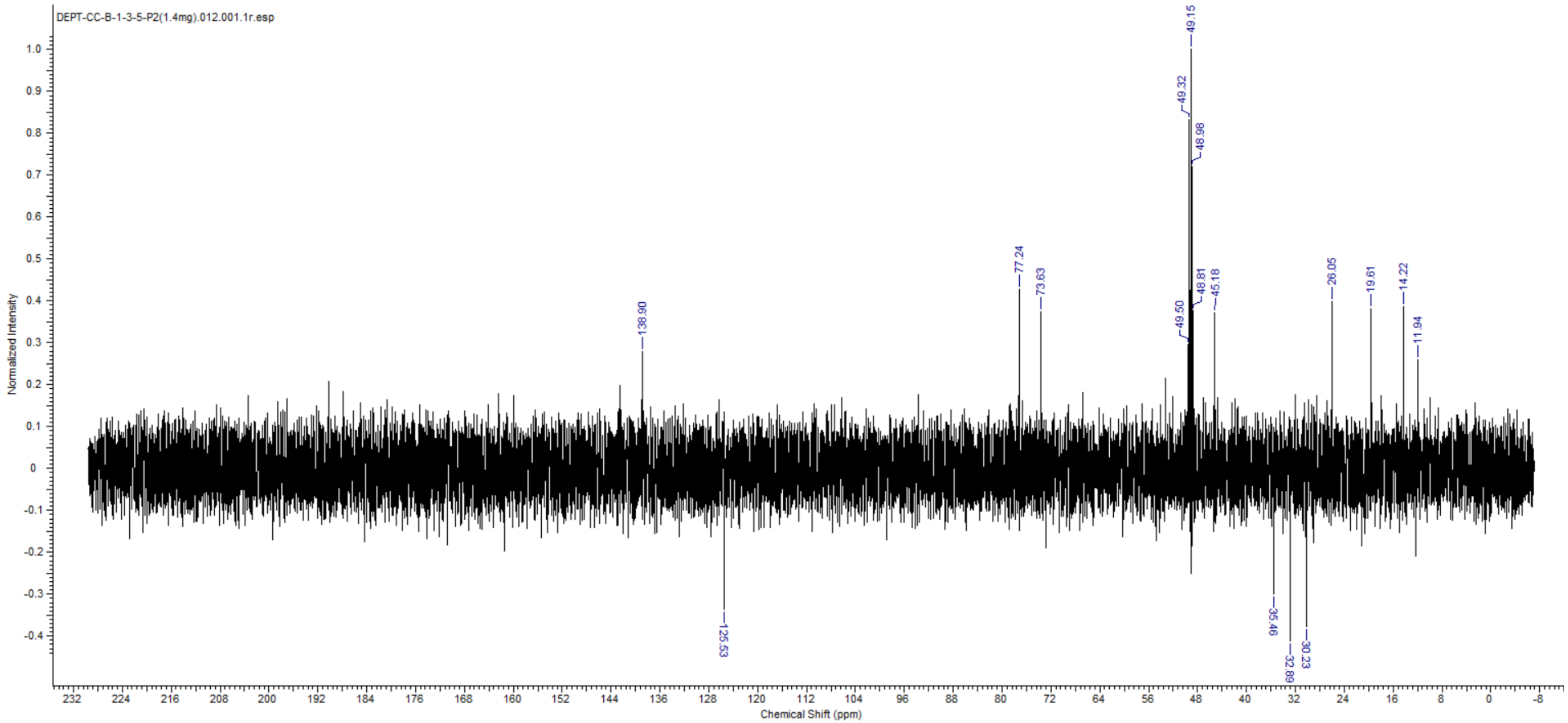

Figure S13. ^{13}C DEPT135 NMR spectrum of **2** in MeOD (125 MHz)

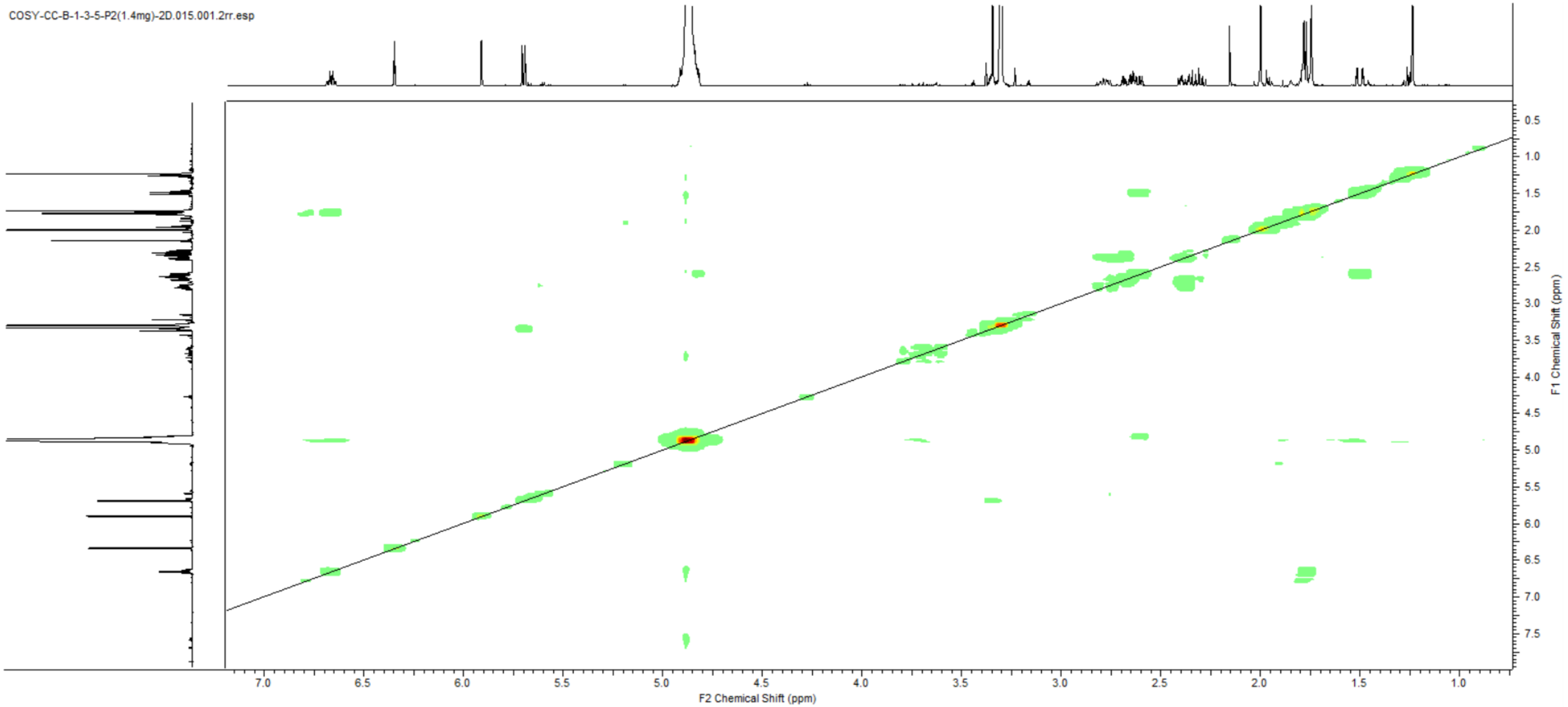

Figure S14. 1H-1H COSY spectrum of **2** in MeOD (500 MHz)

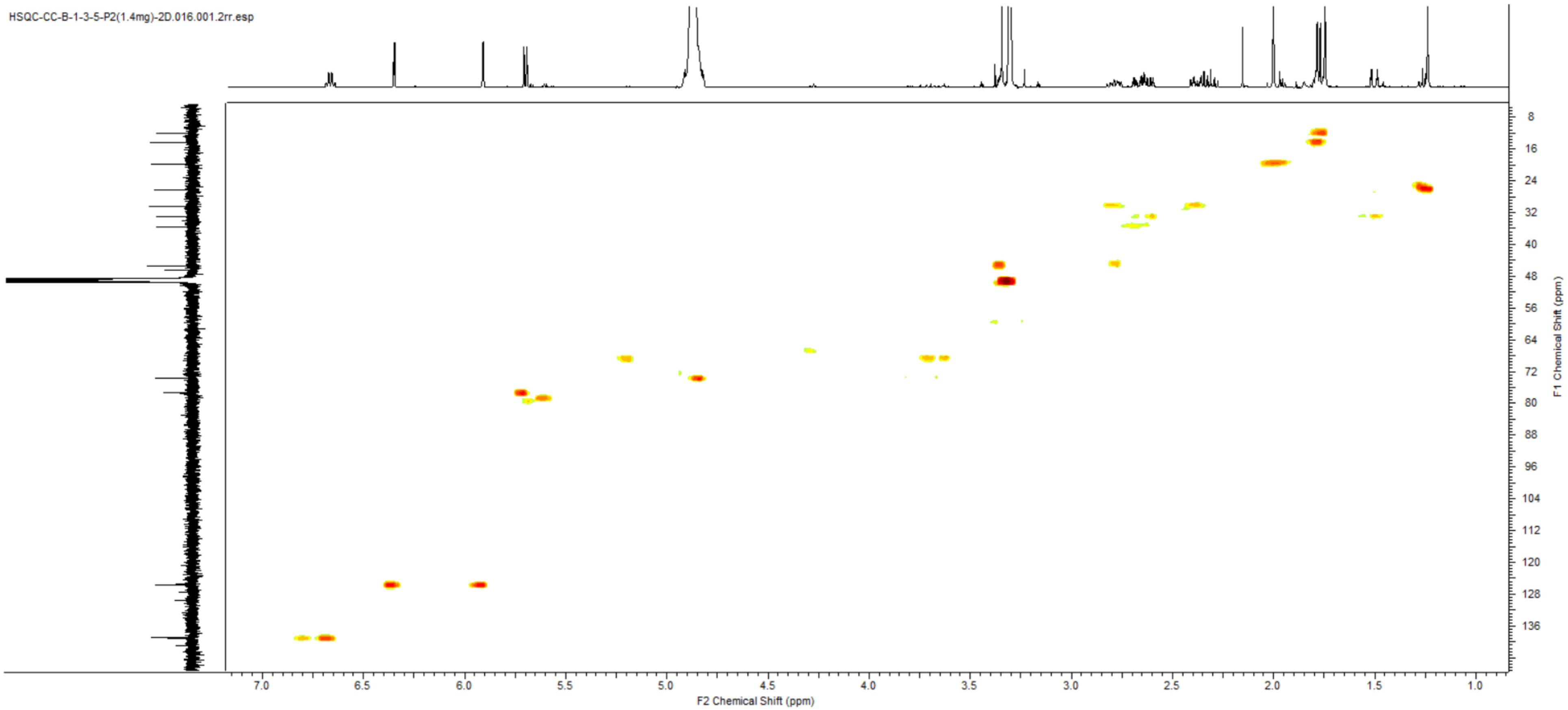

Figure S15. HSQC spectrum of **2** in MeOD (500 MHz)

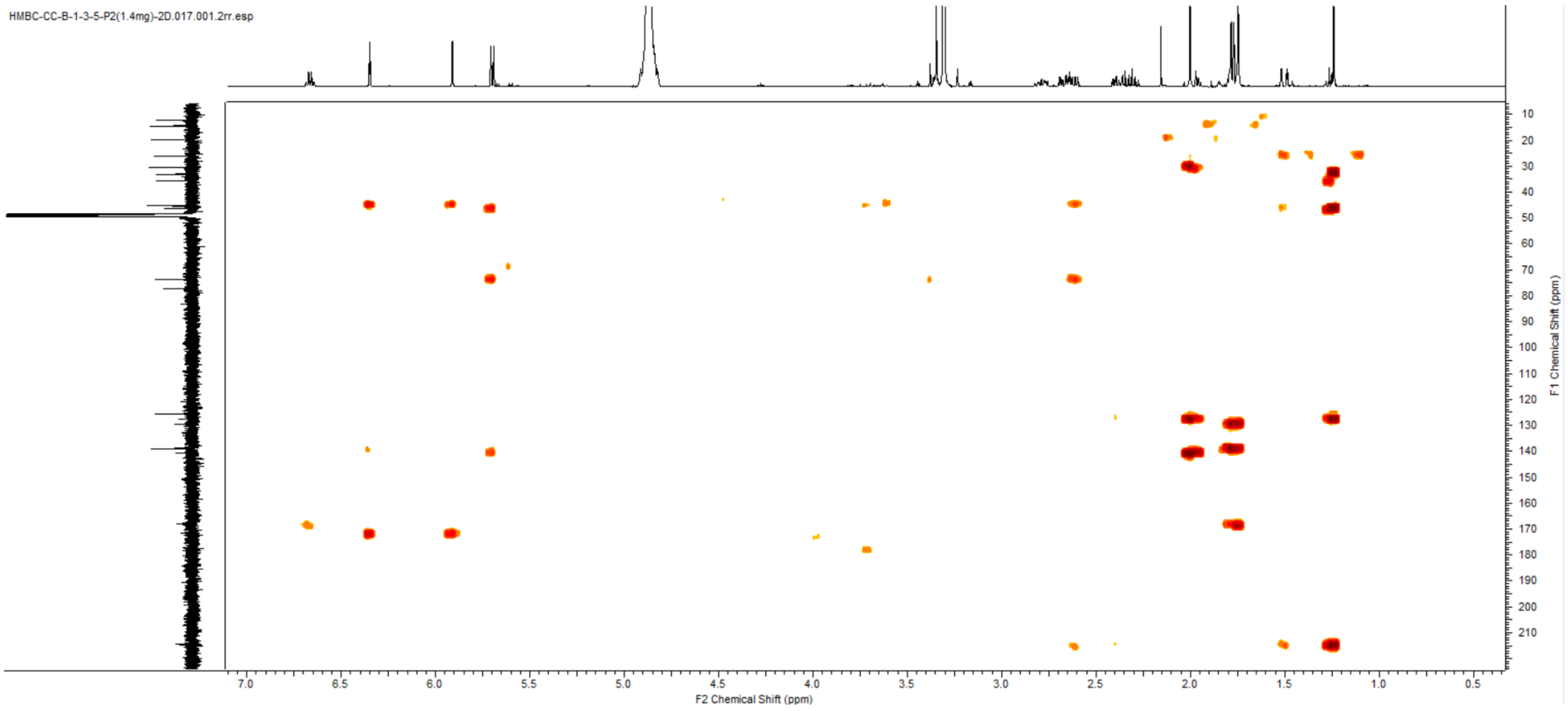

Figure S16. HMBC spectrum of **2** in MeOD (500 MHz)

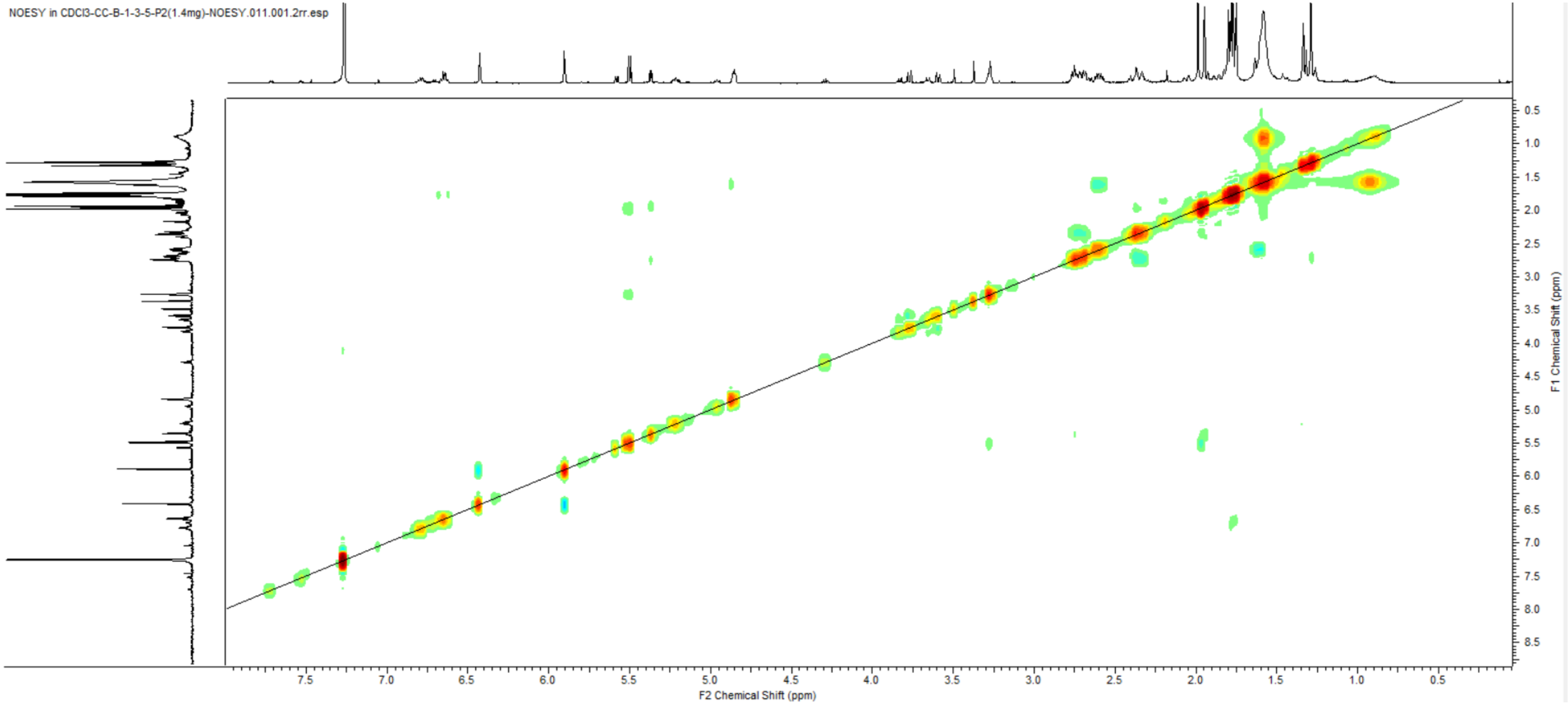

Figure S17. PS-NOESY spectrum of **2** in $CDCl_3$ (500 MHz)

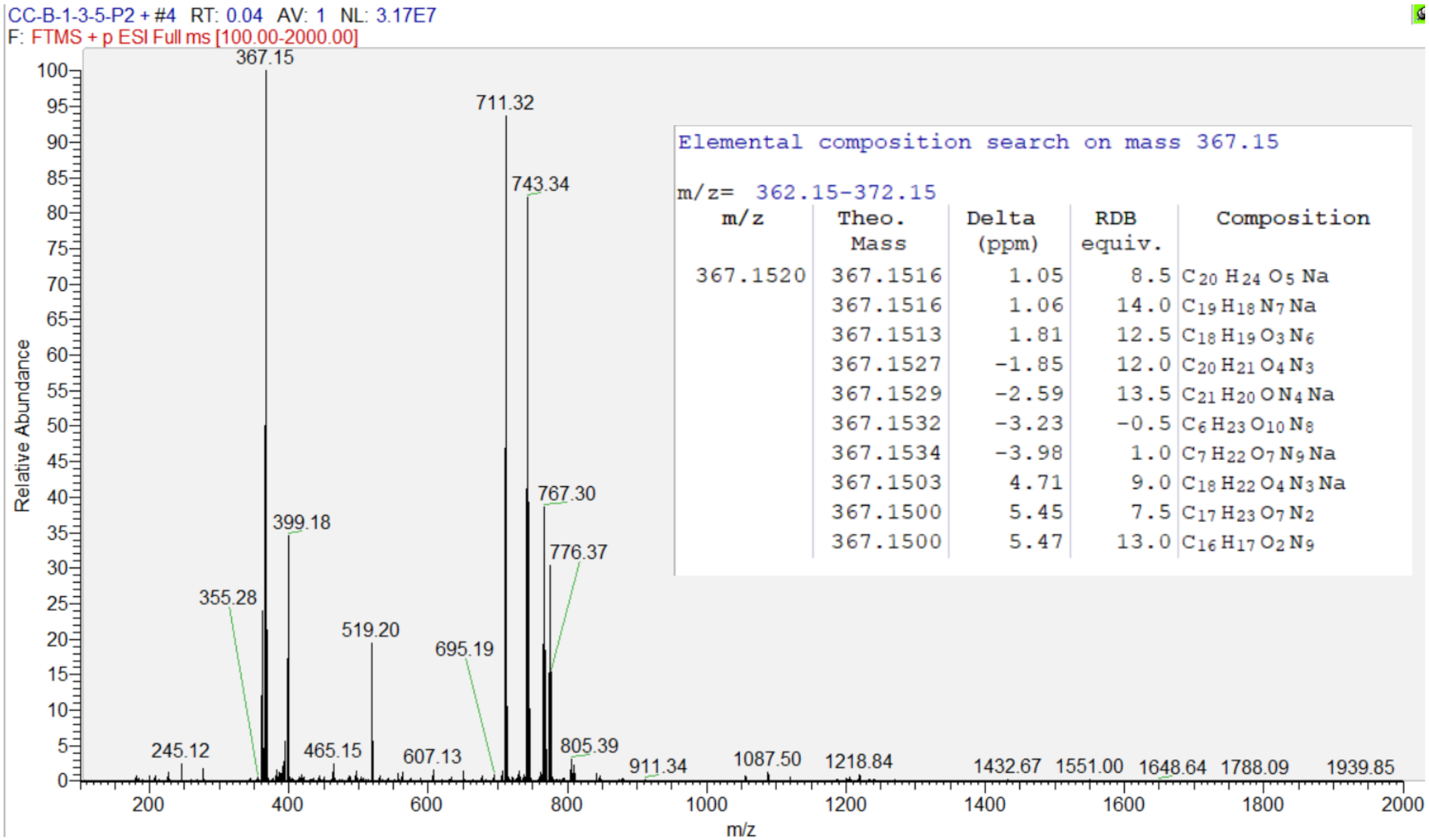

Figure S18. HR-ESI-MS spectrum of **2**

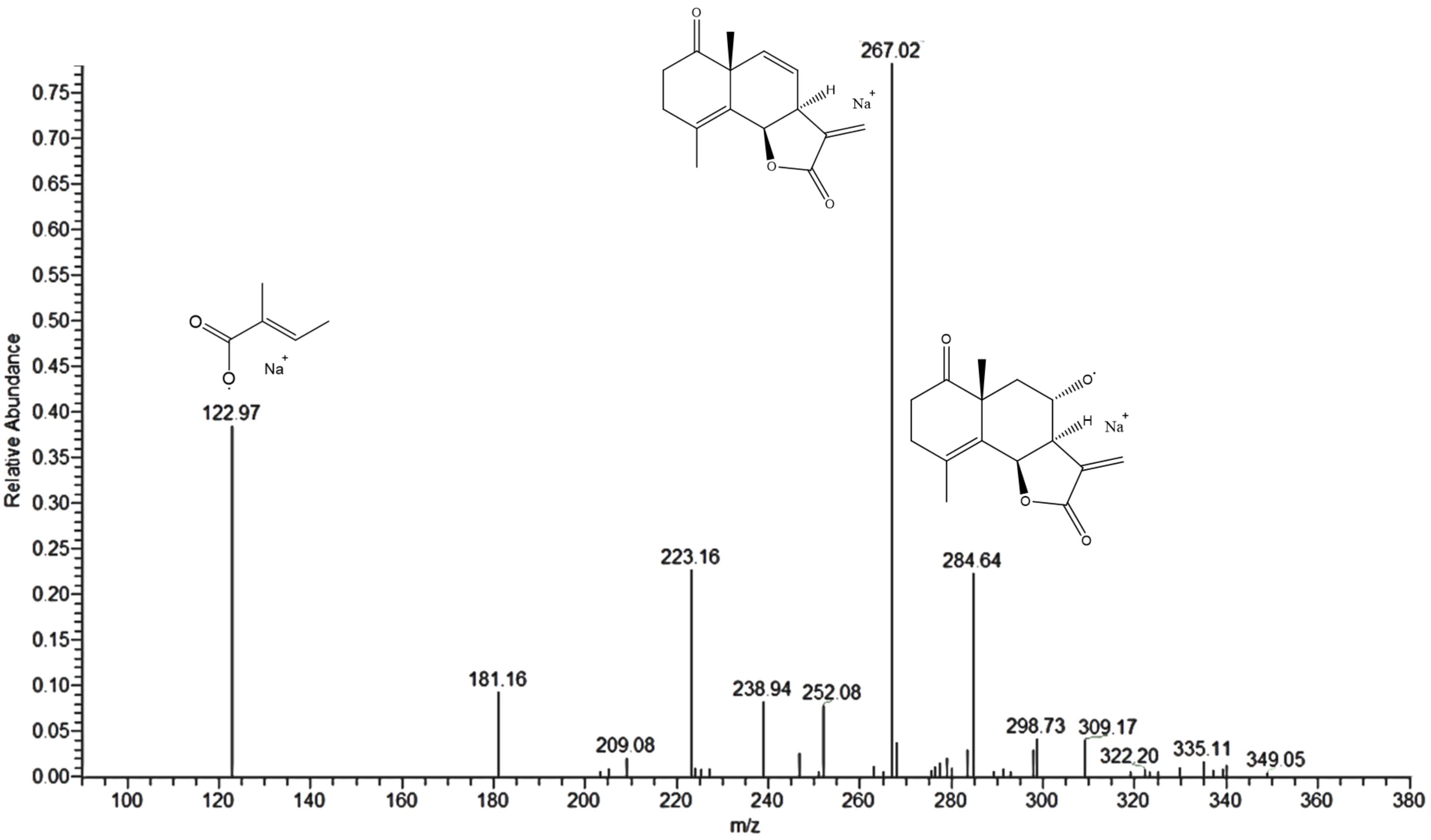

Figure S19. ESI-MS/MS spectrum of **2**

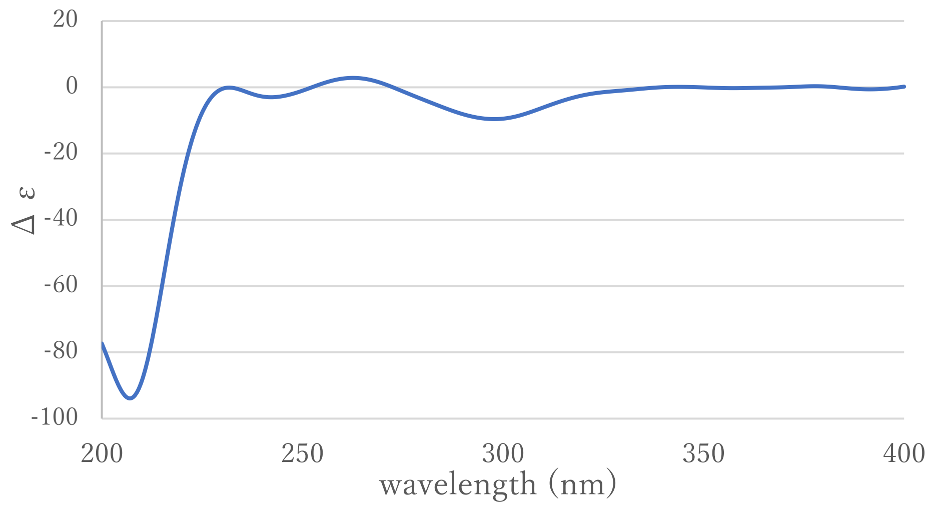

Figure S20. Experimental CD spectrum of **2** in CH_3CN

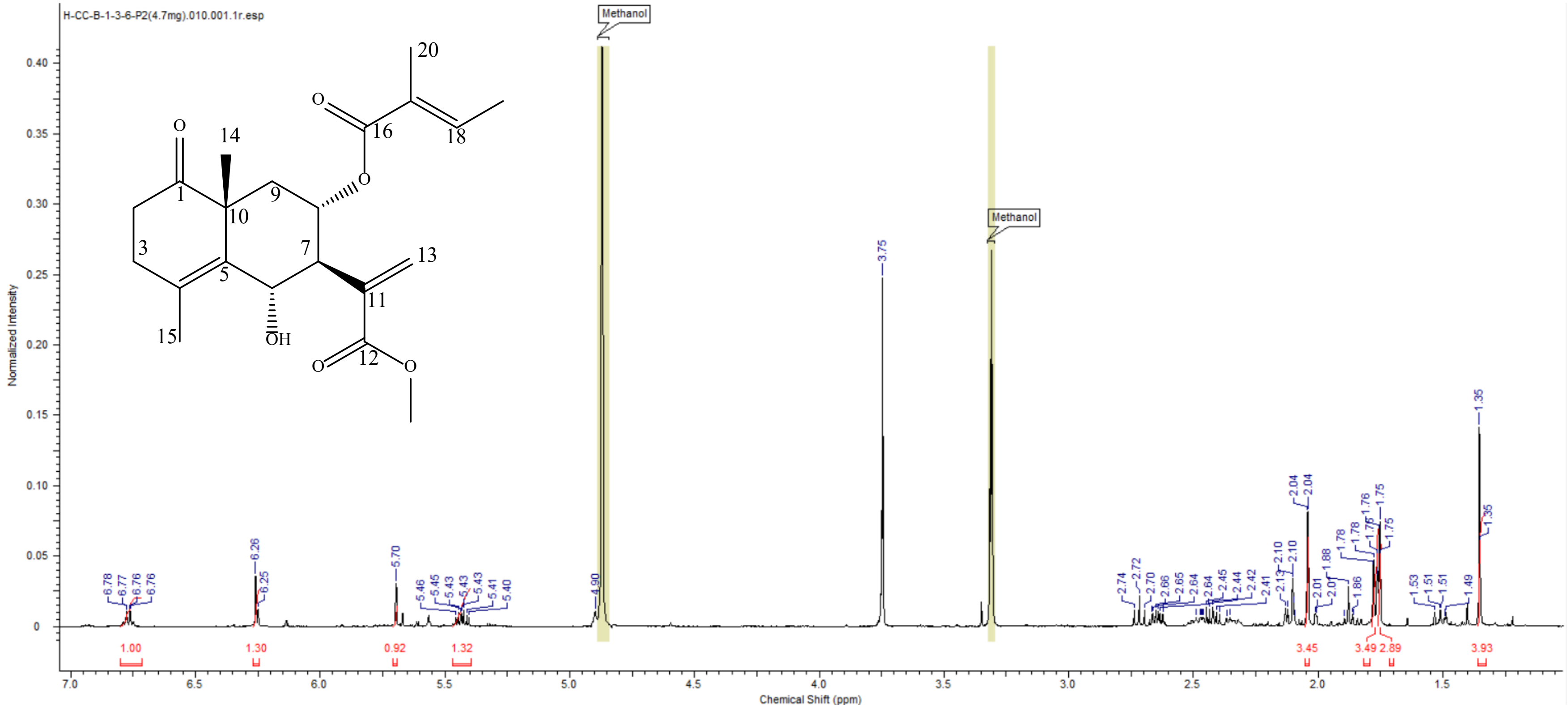

Figure S21. ^{1}H-NMR spectrum of **3** in MeOD (500 MHz)

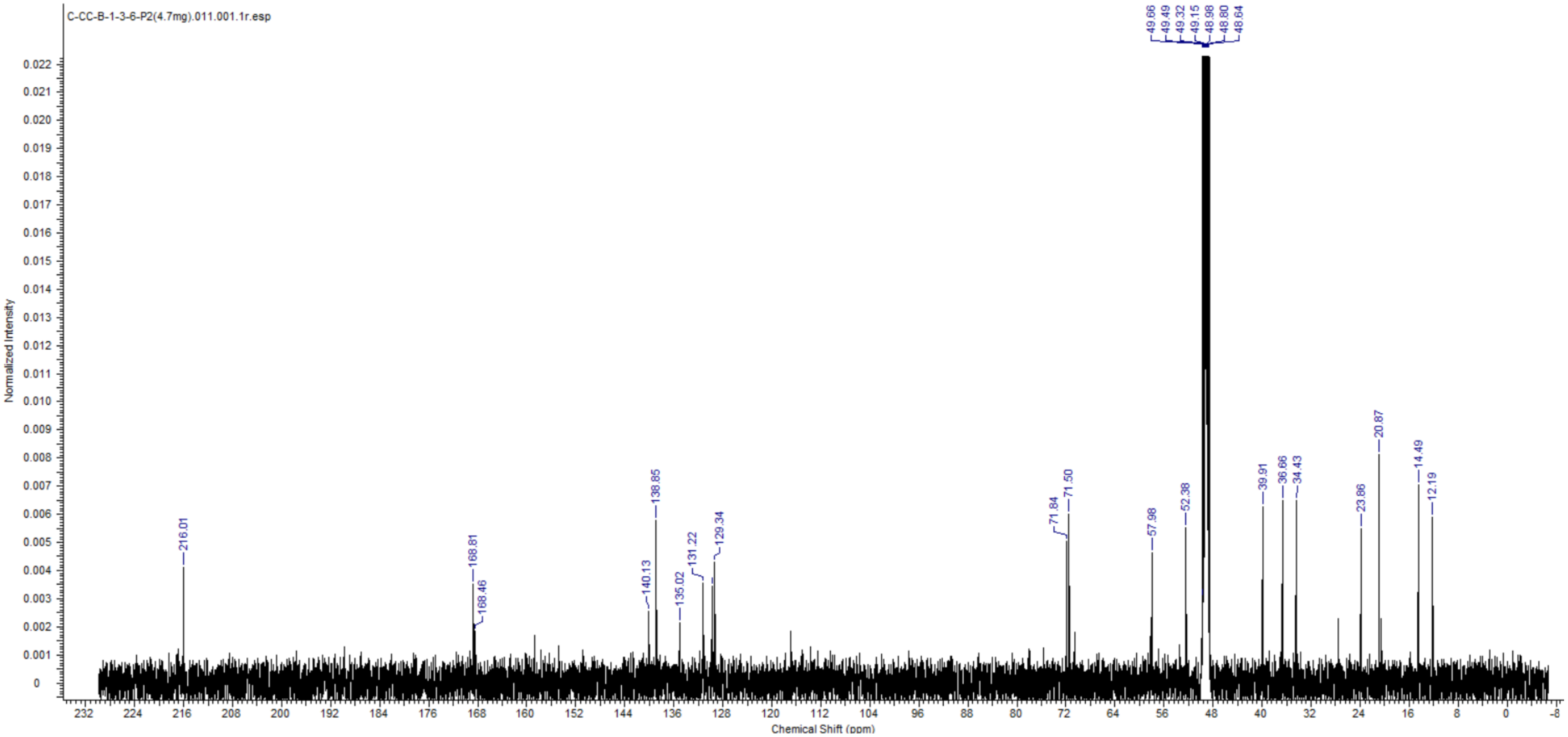

Figure S22. ^{13}C-NMR spectrum of **3** in MeOD (125 MHz)

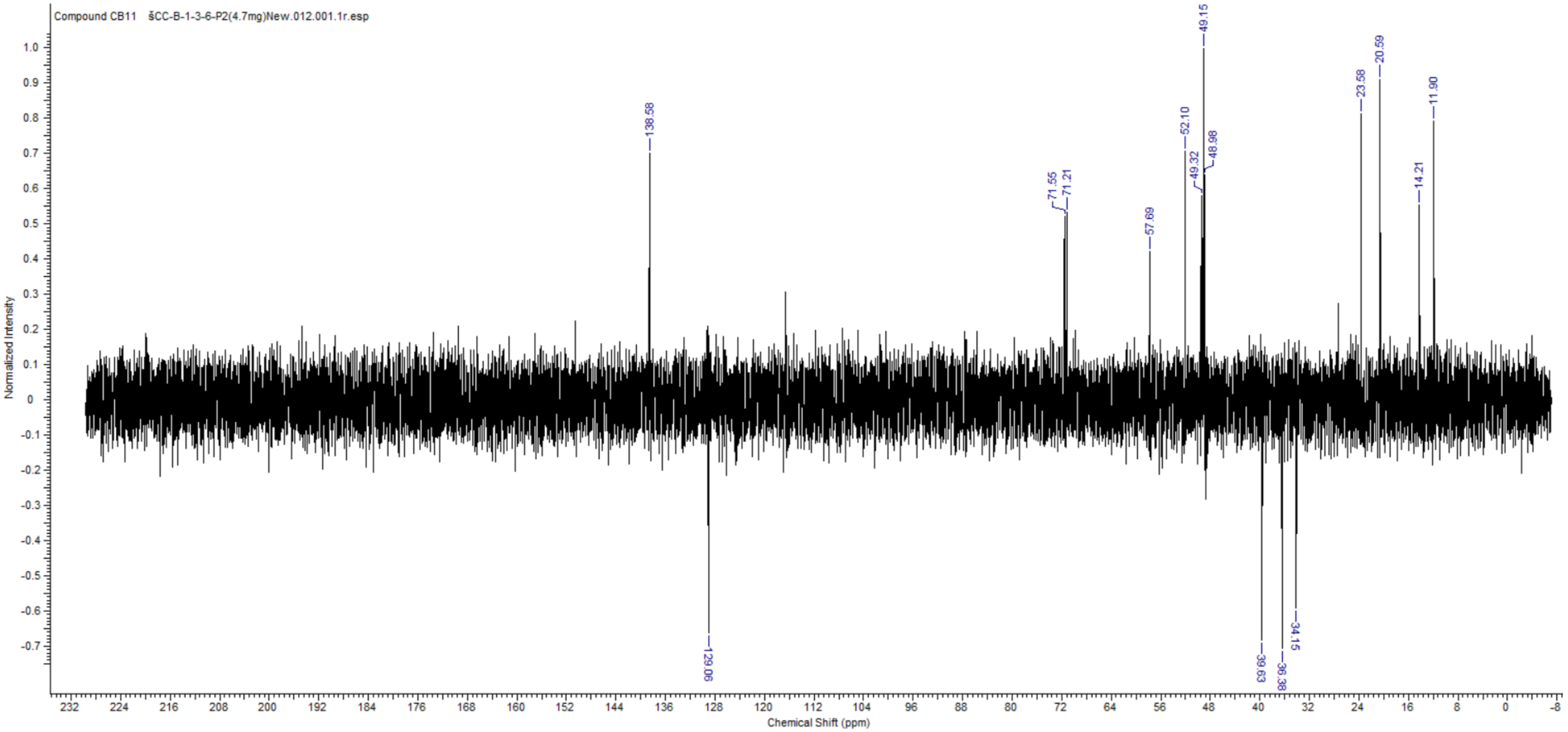

Figure S23. ^{13}C DEPT135 NMR spectrum of **3** in MeOD (125 MHz)

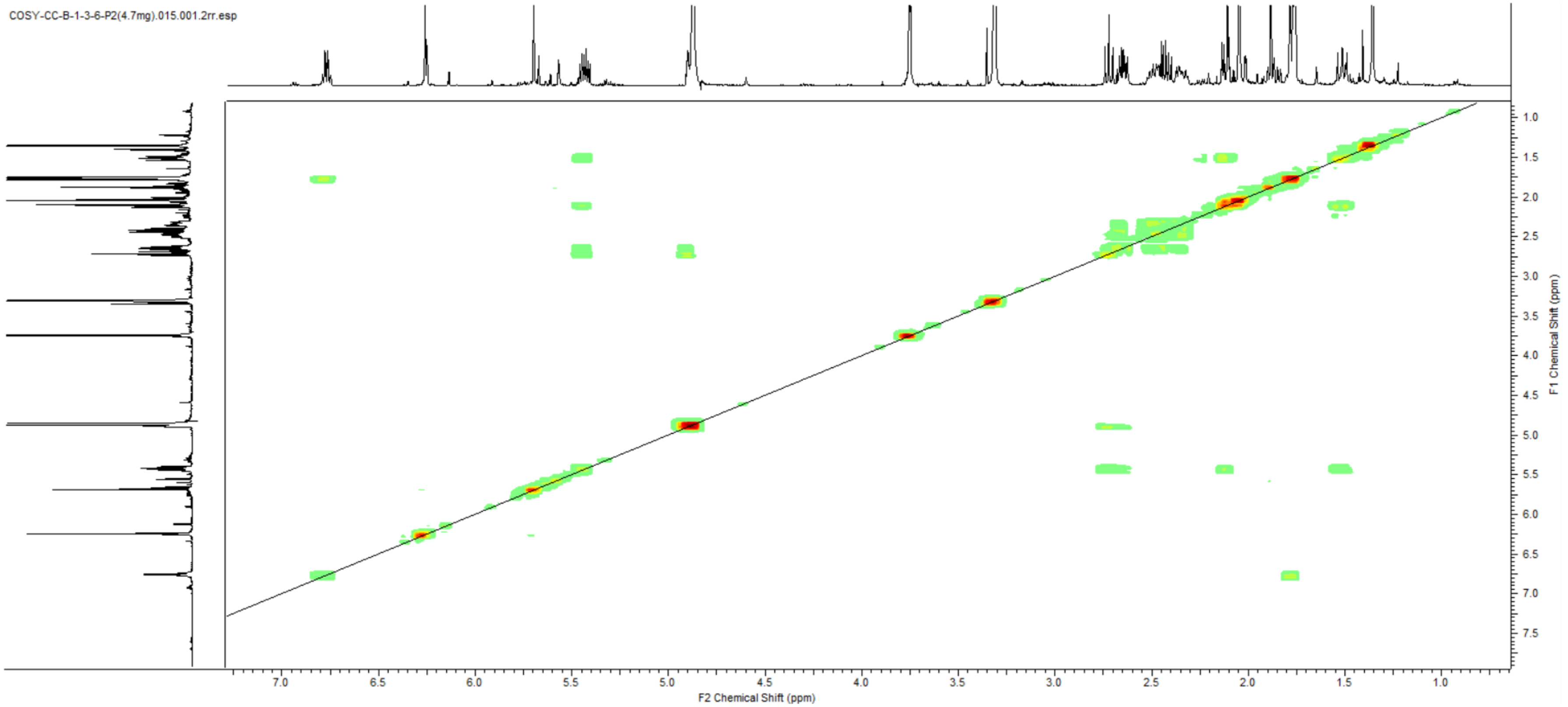

Figure S24. 1H-1H COSY spectrum of **3** in MeOD (500 MHz)

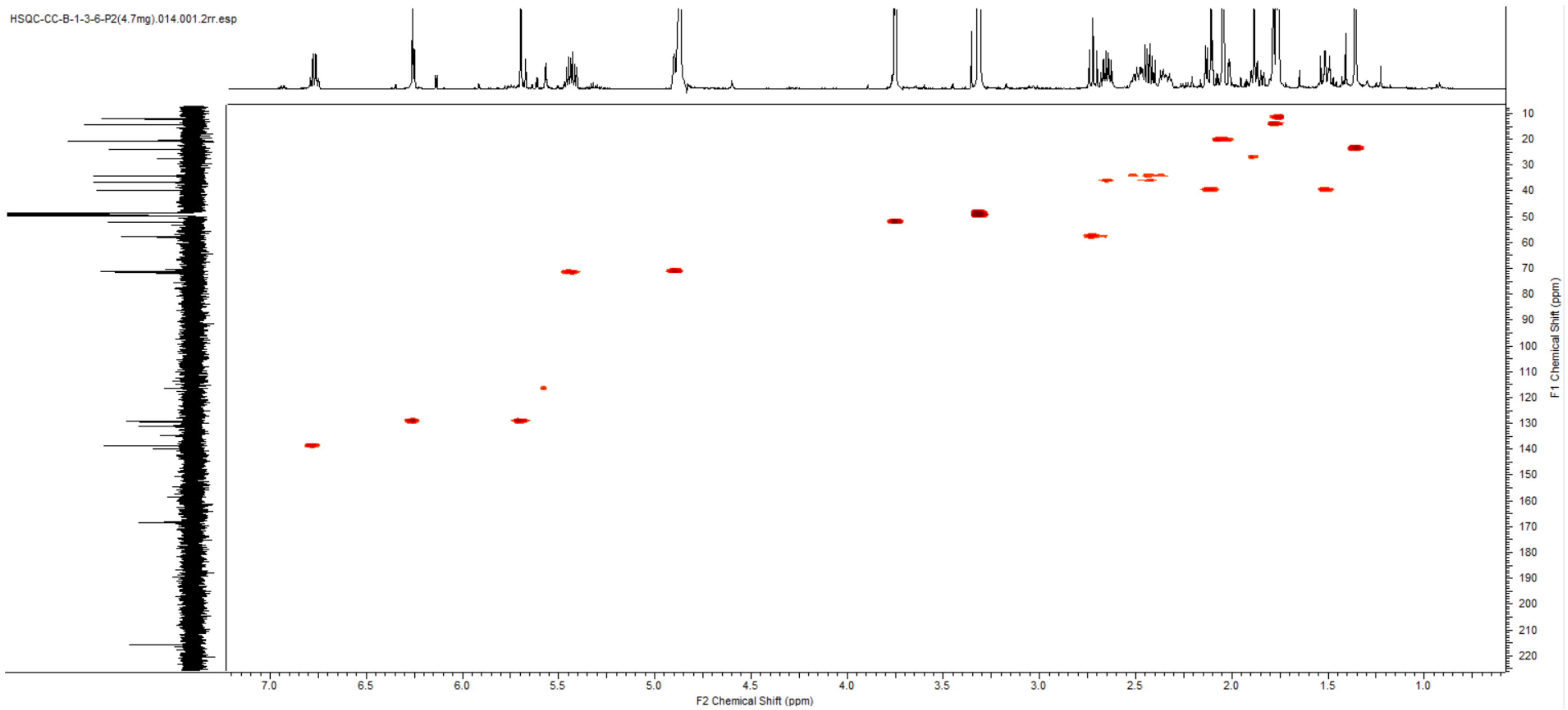

Figure S25. HSQC spectrum of **3** in MeOD (500 MHz)

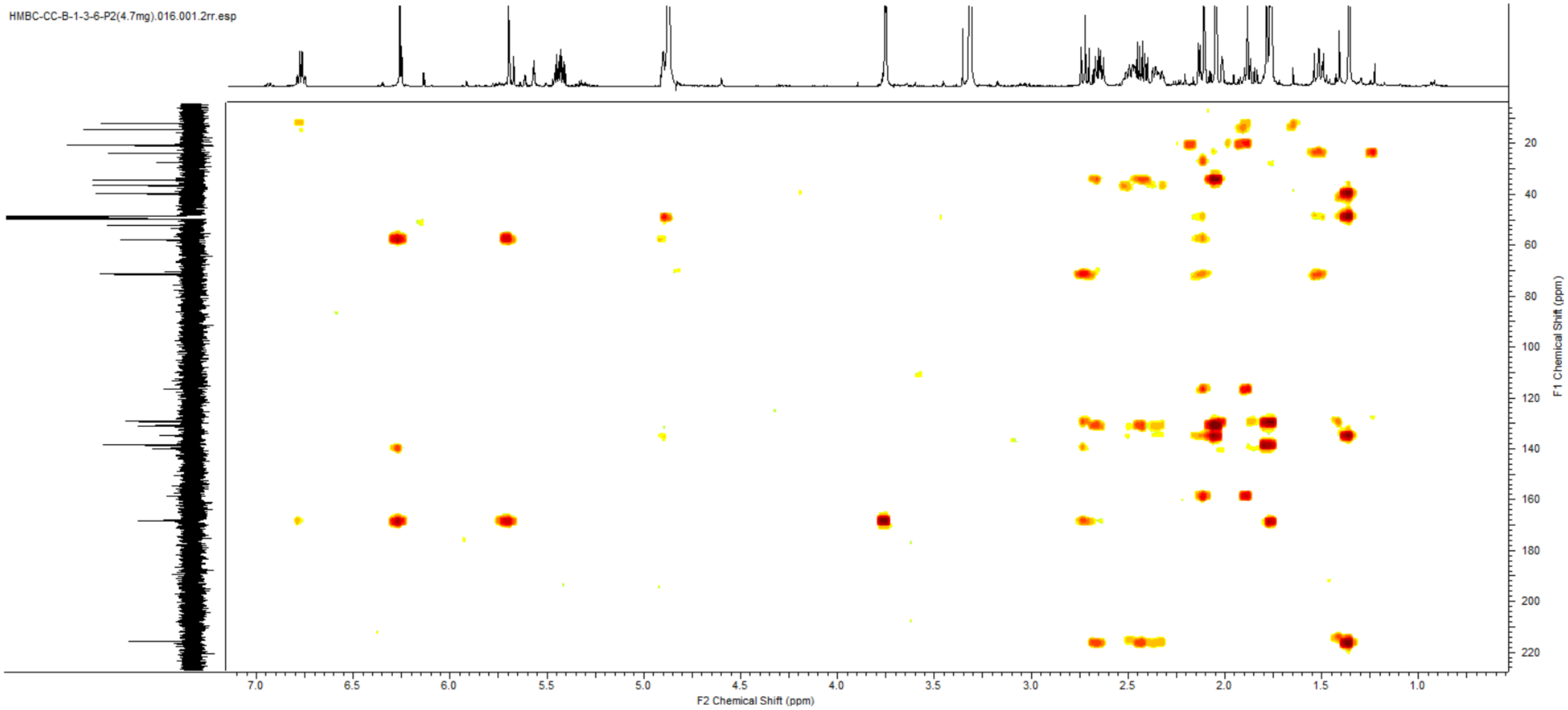

Figure S26. HMBC spectrum of **3** in MeOD (500 MHz)

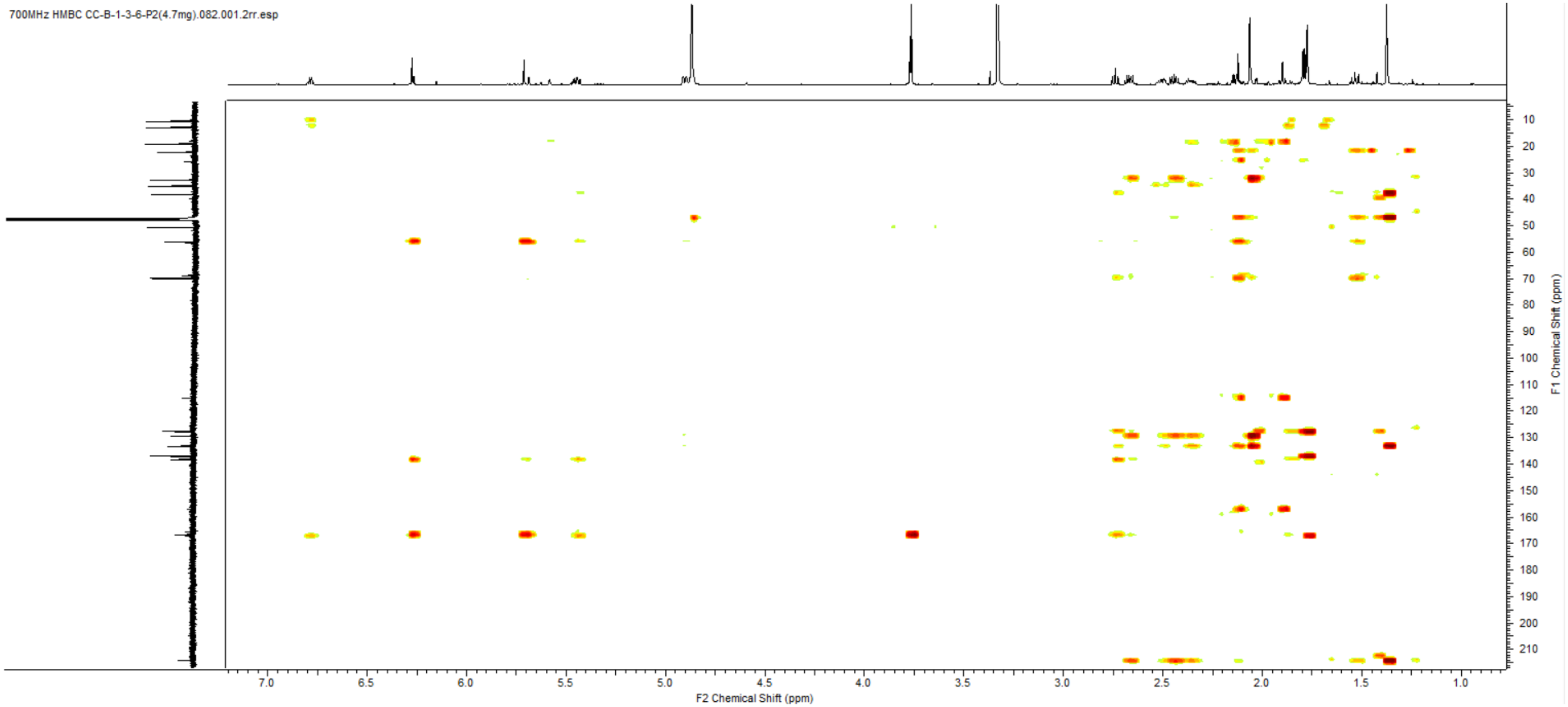

Figure S27. HMBC spectrum of **3** in MeOD (700 MHz)

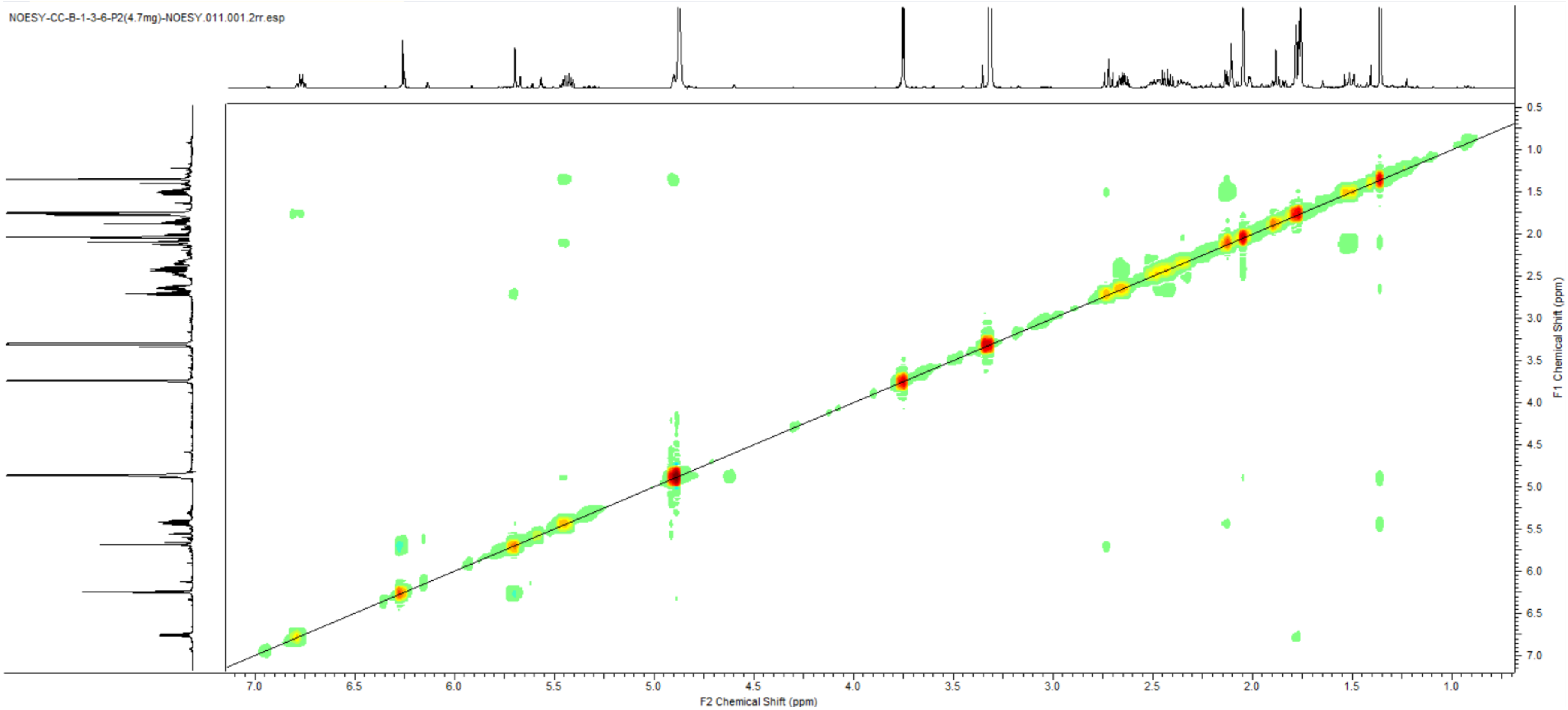

Figure S28. PS-NOESY spectrum of **3** in MeOD (500 MHz)

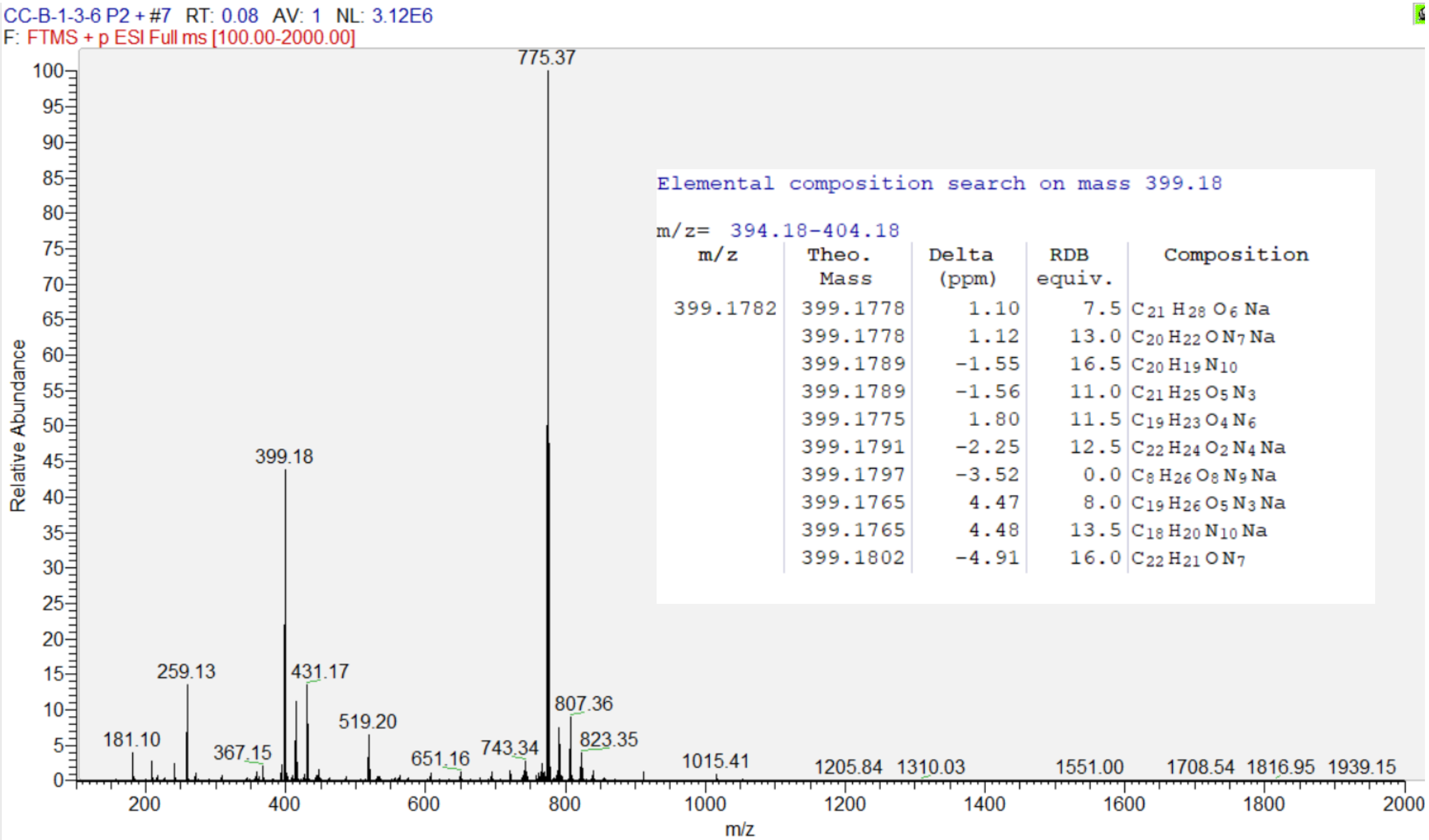

m/z	Theo. Mass	Delta (ppm)	RDB equiv.	Composition
399.1782	399.1778	1.10	7.5	$C_{21}H_{28}O_6Na$
	399.1778	1.12	13.0	$C_{20}H_{22}ON_7Na$
	399.1789	-1.55	16.5	$C_{20}H_{19}N_{10}$
	399.1789	-1.56	11.0	$C_{21}H_{25}O_5N_3$
	399.1775	1.80	11.5	$C_{19}H_{23}O_4N_6$
	399.1791	-2.25	12.5	$C_{22}H_{24}O_2N_4Na$
	399.1797	-3.52	0.0	$C_8H_{26}O_8N_9Na$
	399.1765	4.47	8.0	$C_{19}H_{26}O_5N_3Na$
	399.1765	4.48	13.5	$C_{18}H_{20}N_{10}Na$
	399.1802	-4.91	16.0	$C_{22}H_{21}ON_7$

Figure S29. HR-ESI-MS spectrum of **3**

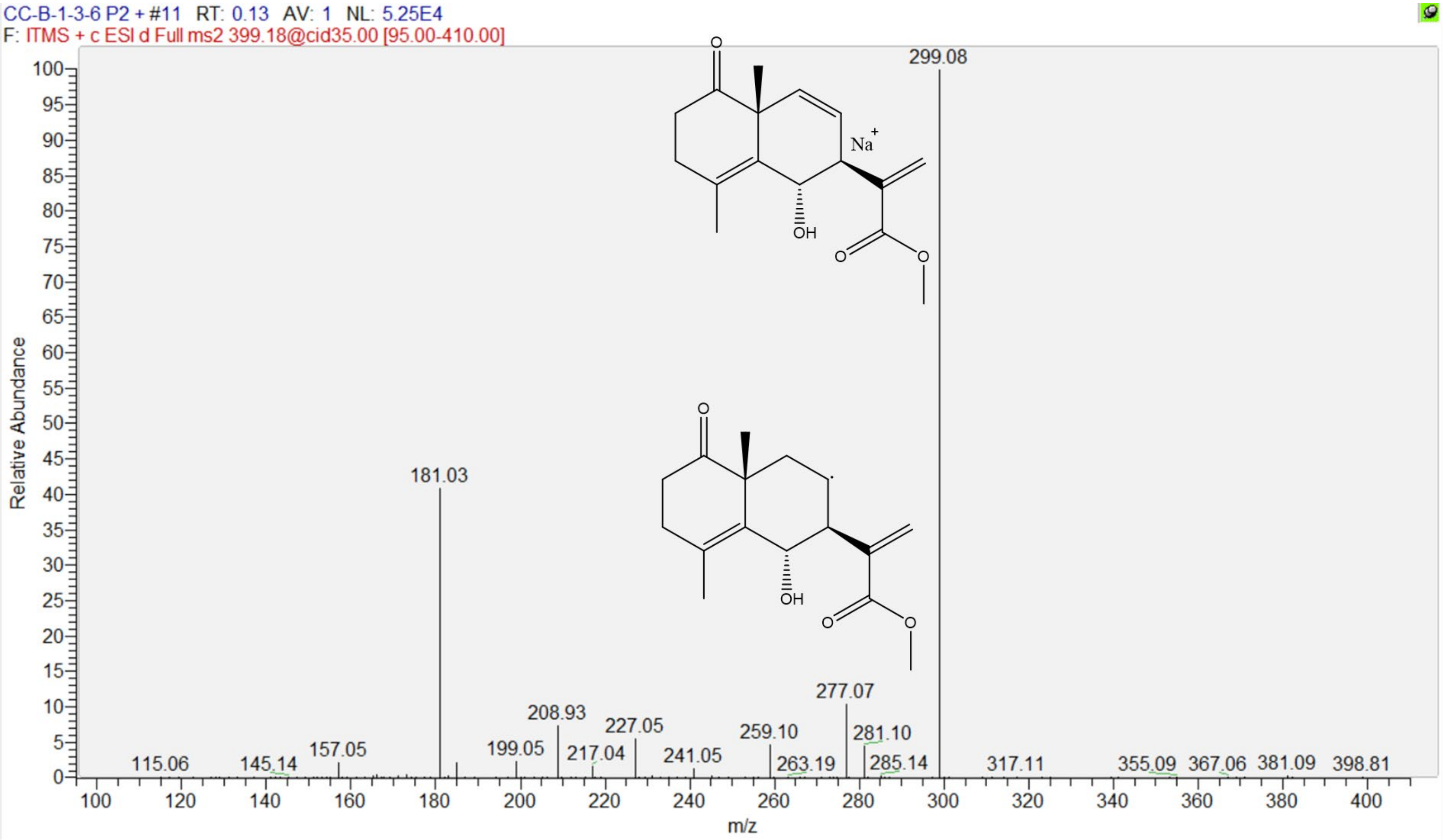

Figure S30. ESI-MS/MS spectrum of **3**

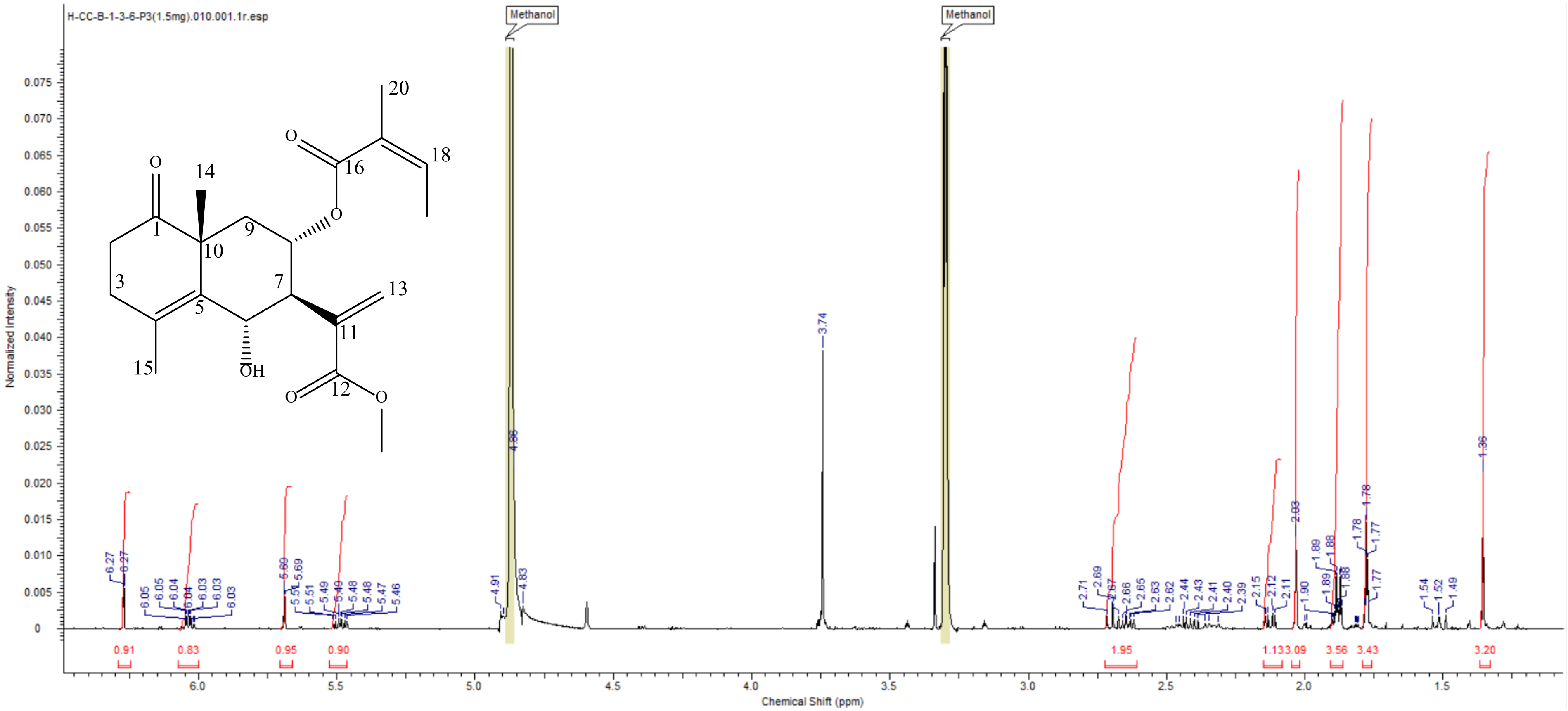

Figure S31. ^{1}H-NMR spectrum of **4** in MeOD (500 MHz)

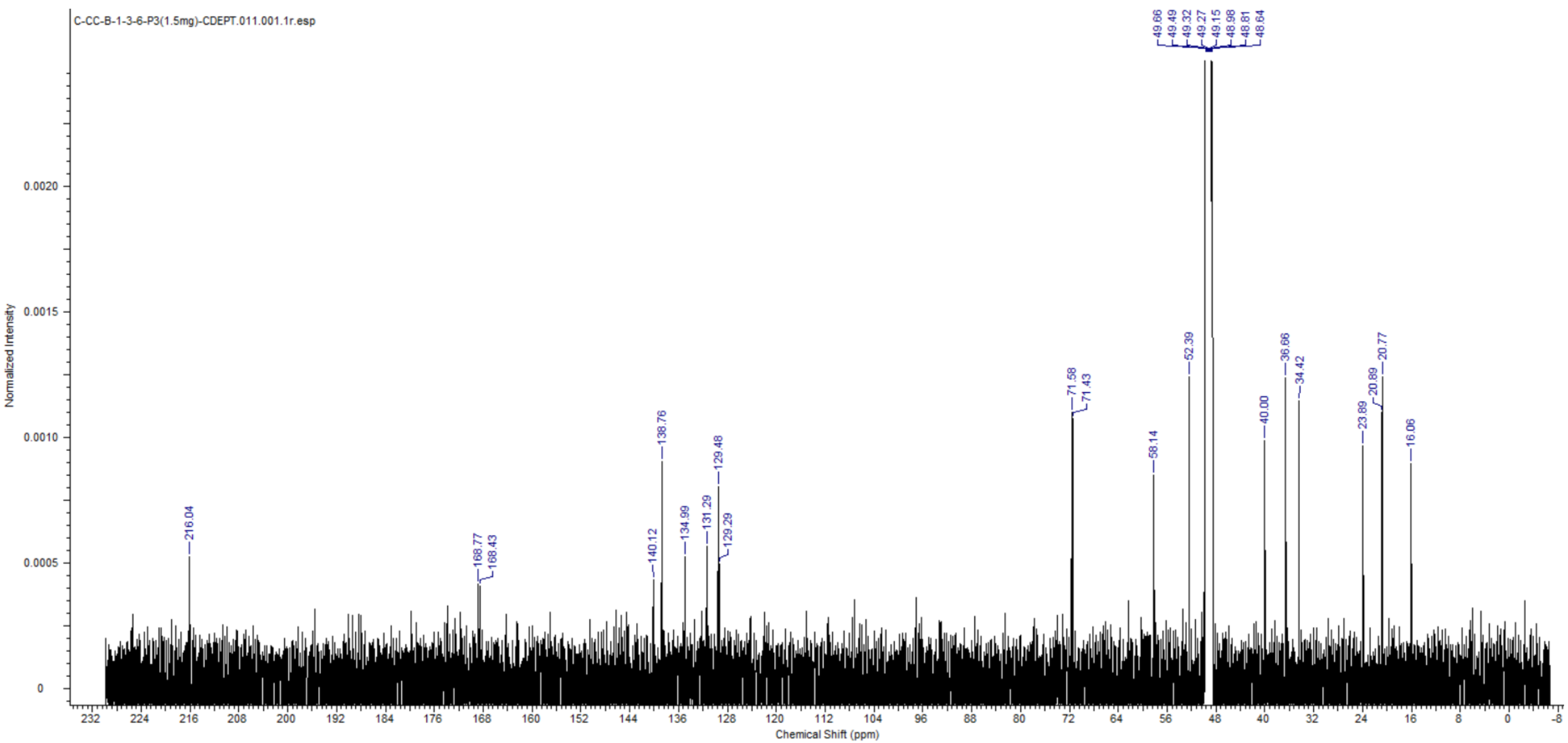

Figure S32. ^{13}C-NMR spectrum of **4** in MeOD (125 MHz)

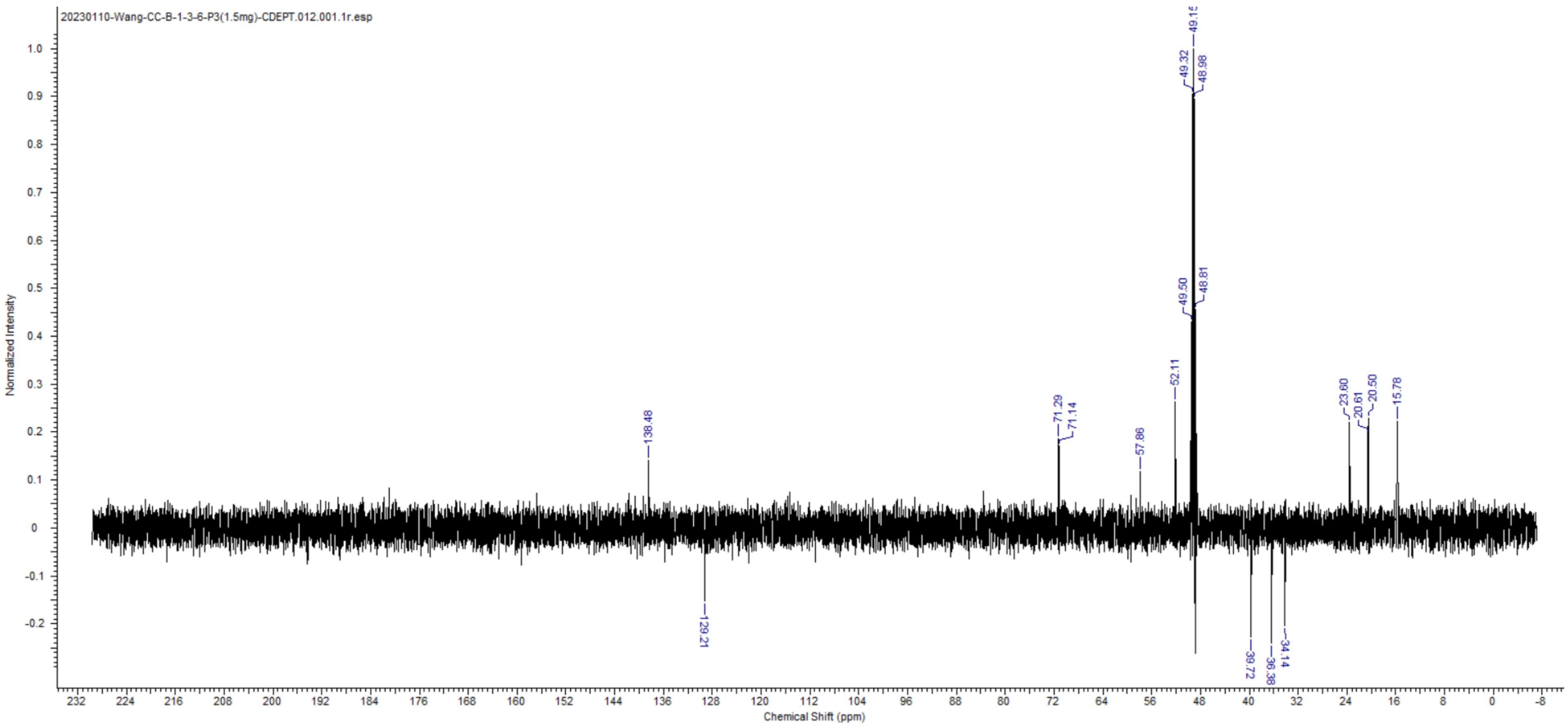

Figure S33. ^{13}C DEPT135 NMR spectrum of **4** in MeOD (125 MHz)

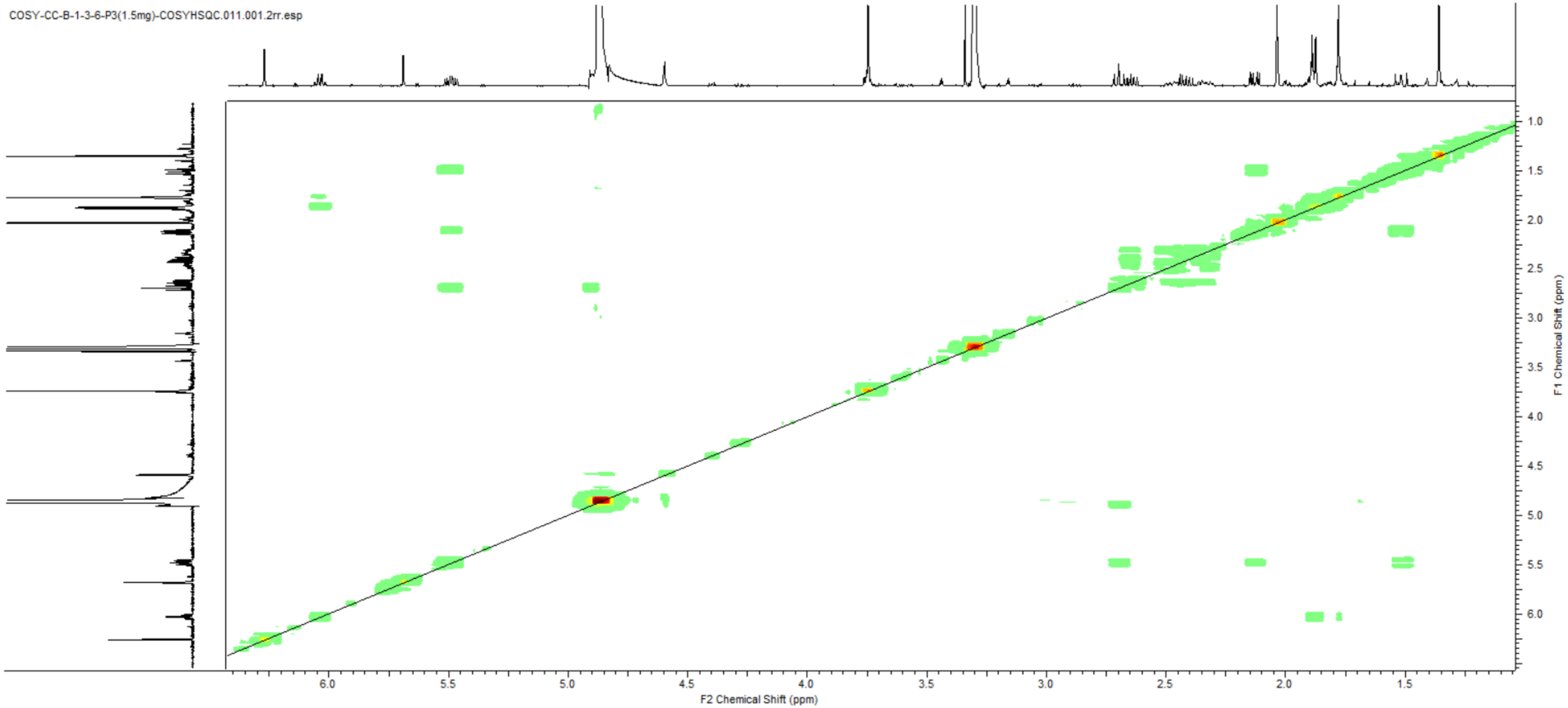

Figure S34. 1H-1H COSY spectrum of **4** in MeOD (500 MHz)

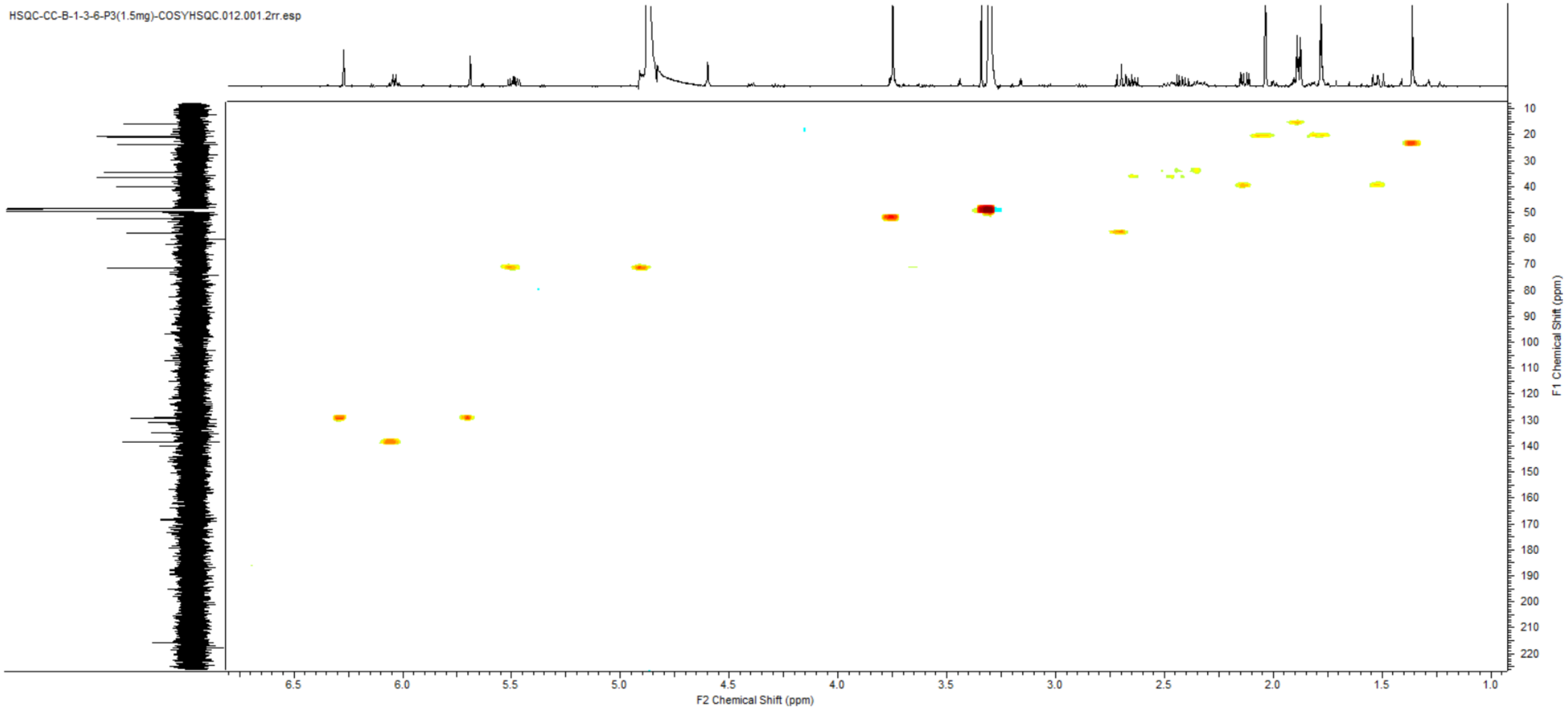

Figure S35. HSQC spectrum of **4** in MeOD (500 MHz)

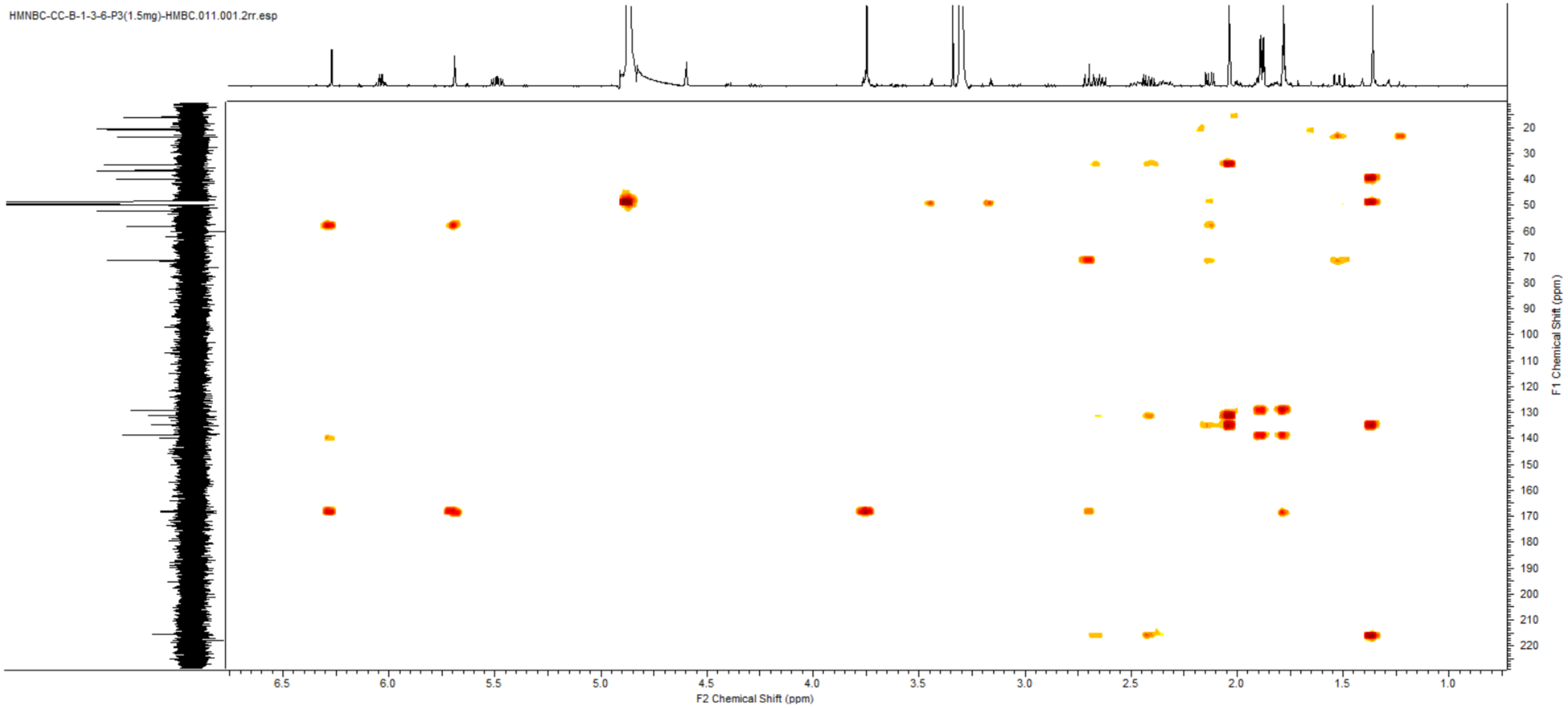

Figure S36. HMBC spectrum of **4** in MeOD (500 MHz)

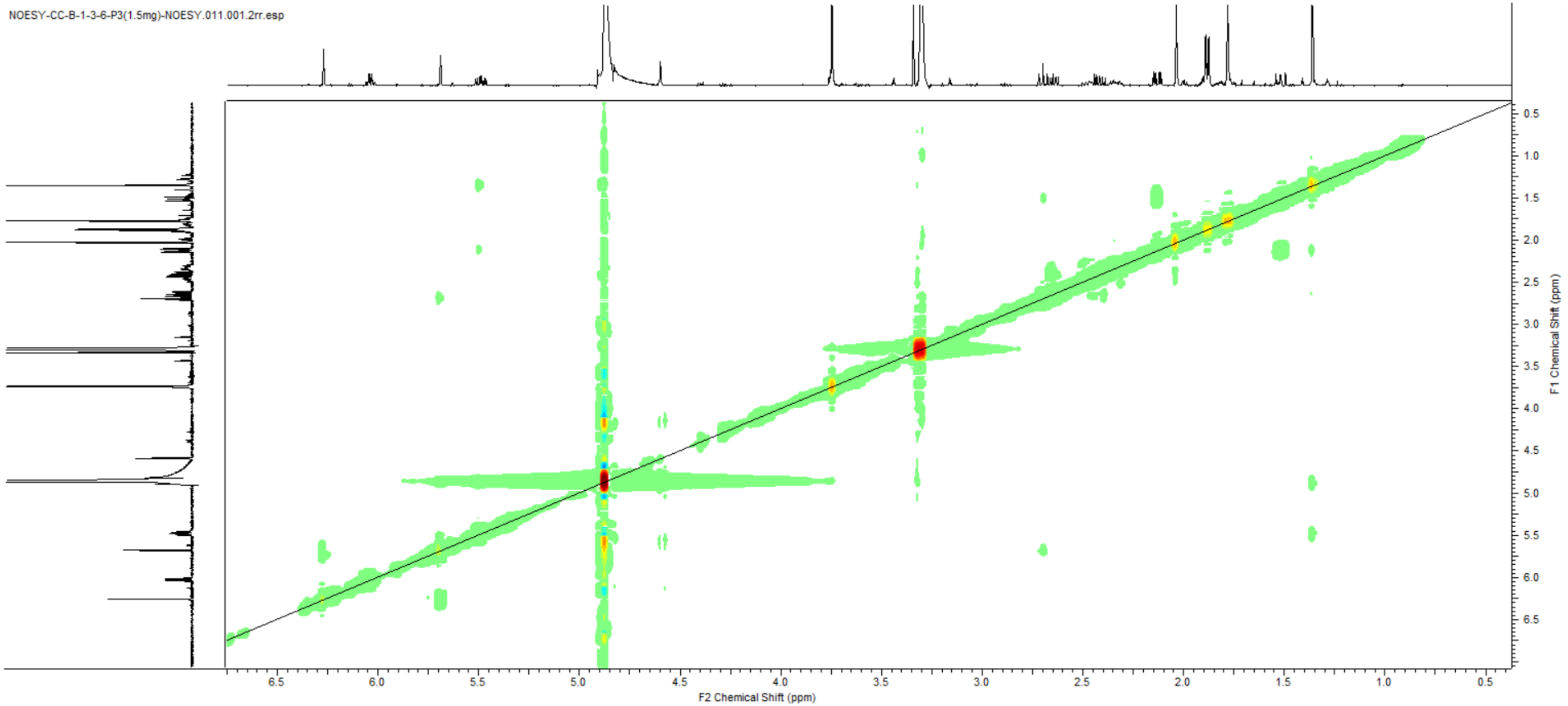

Figure S37. PS-NOESY spectrum of **4** in MeOD (500 MHz)

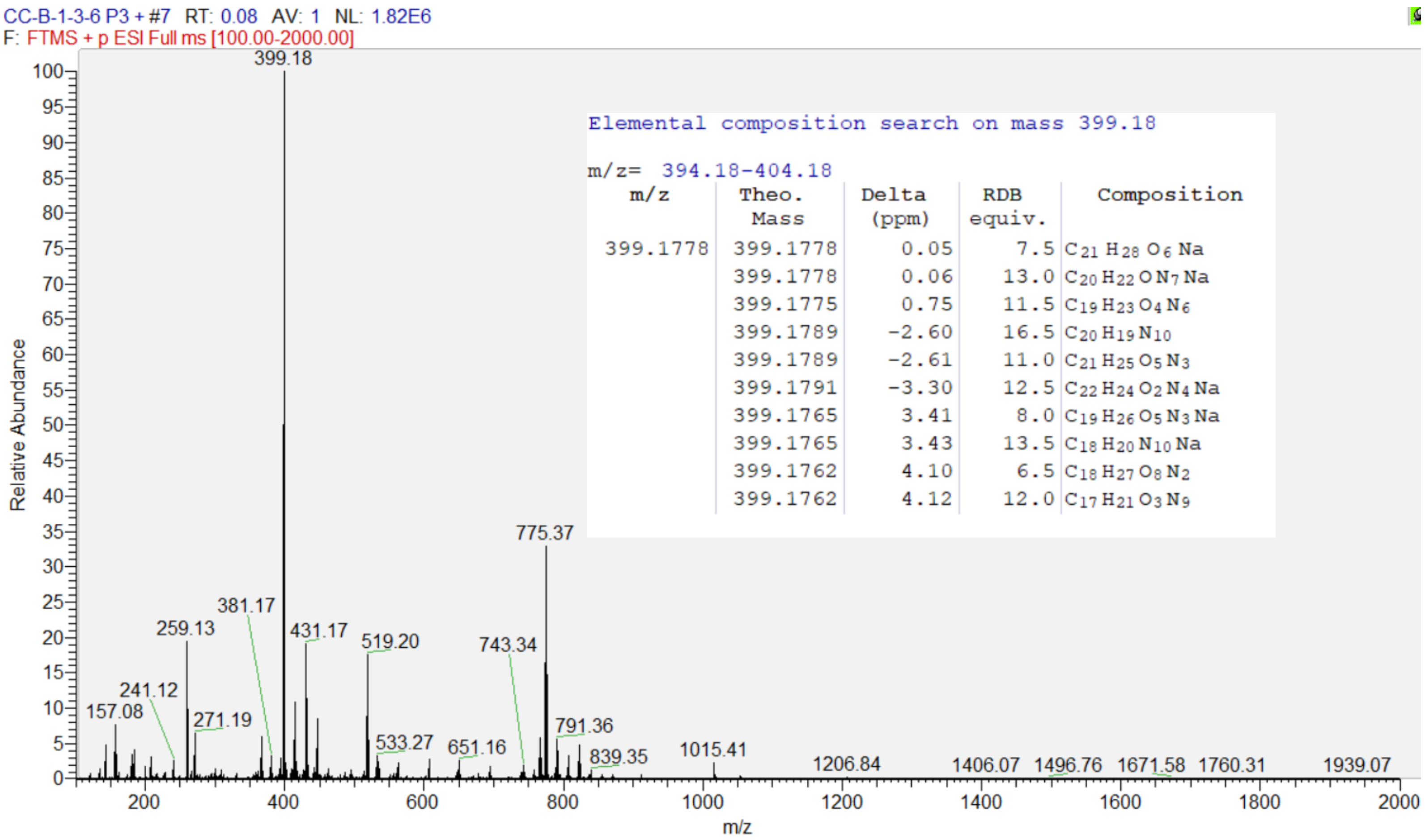

Figure S38. HR-ESI-MS spectrum of **4**

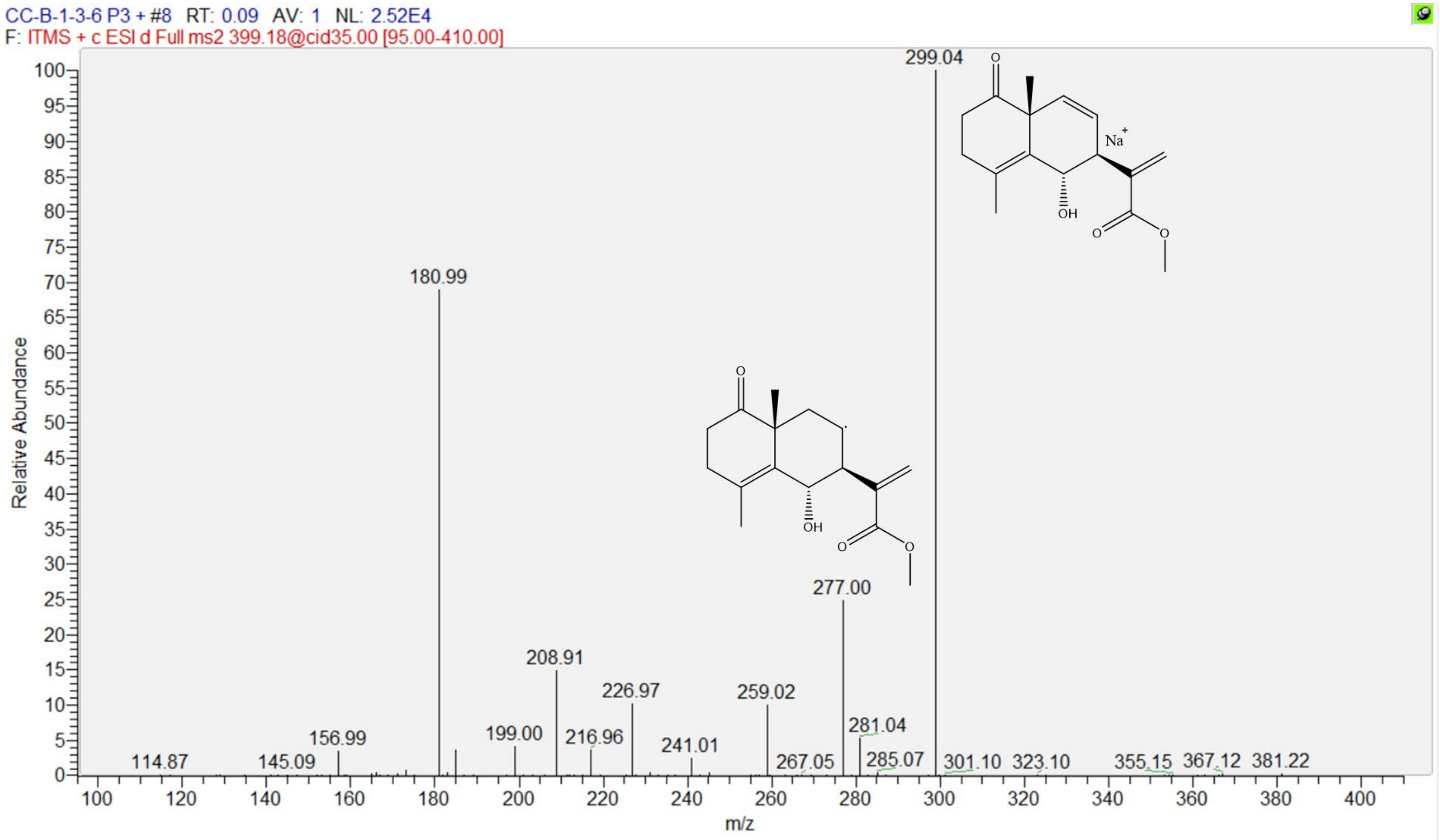

Figure S39. ESI-MS/MS spectrum of **4**

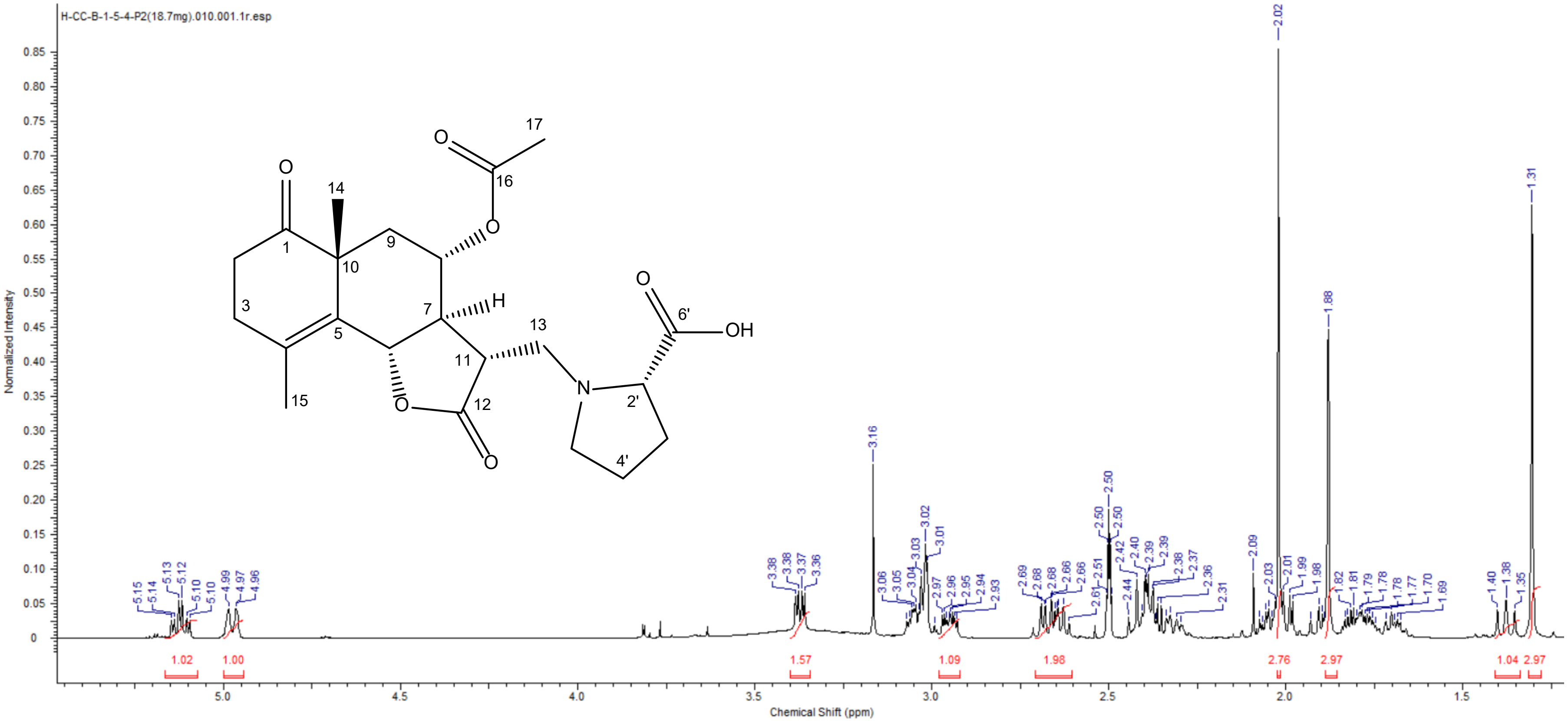

Figure S40. ^{1}H-NMR spectrum of **5** in DMSO-d_6 (500 MHz)

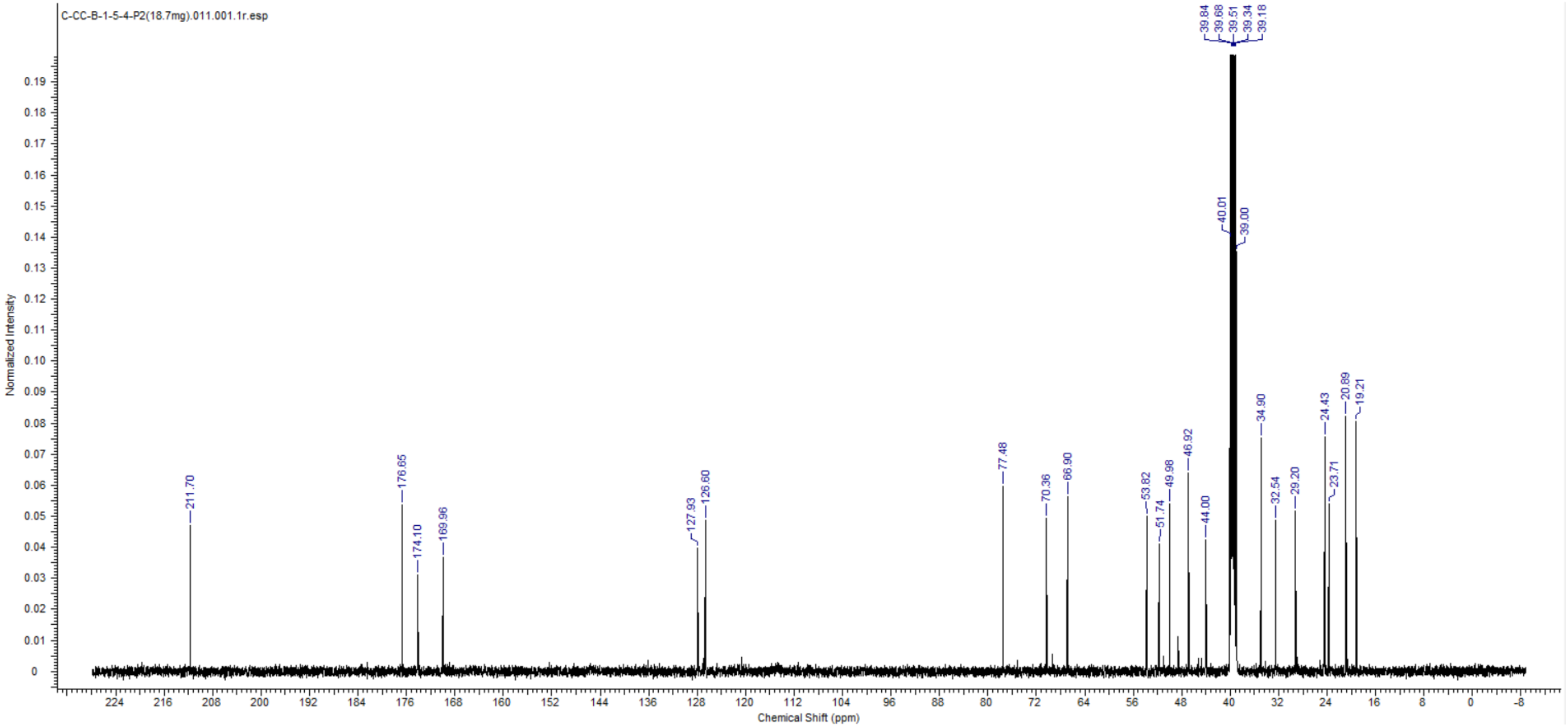

Figure S41. ^{13}C-NMR spectrum of **5** in DMSO-d_6 (125 MHz)

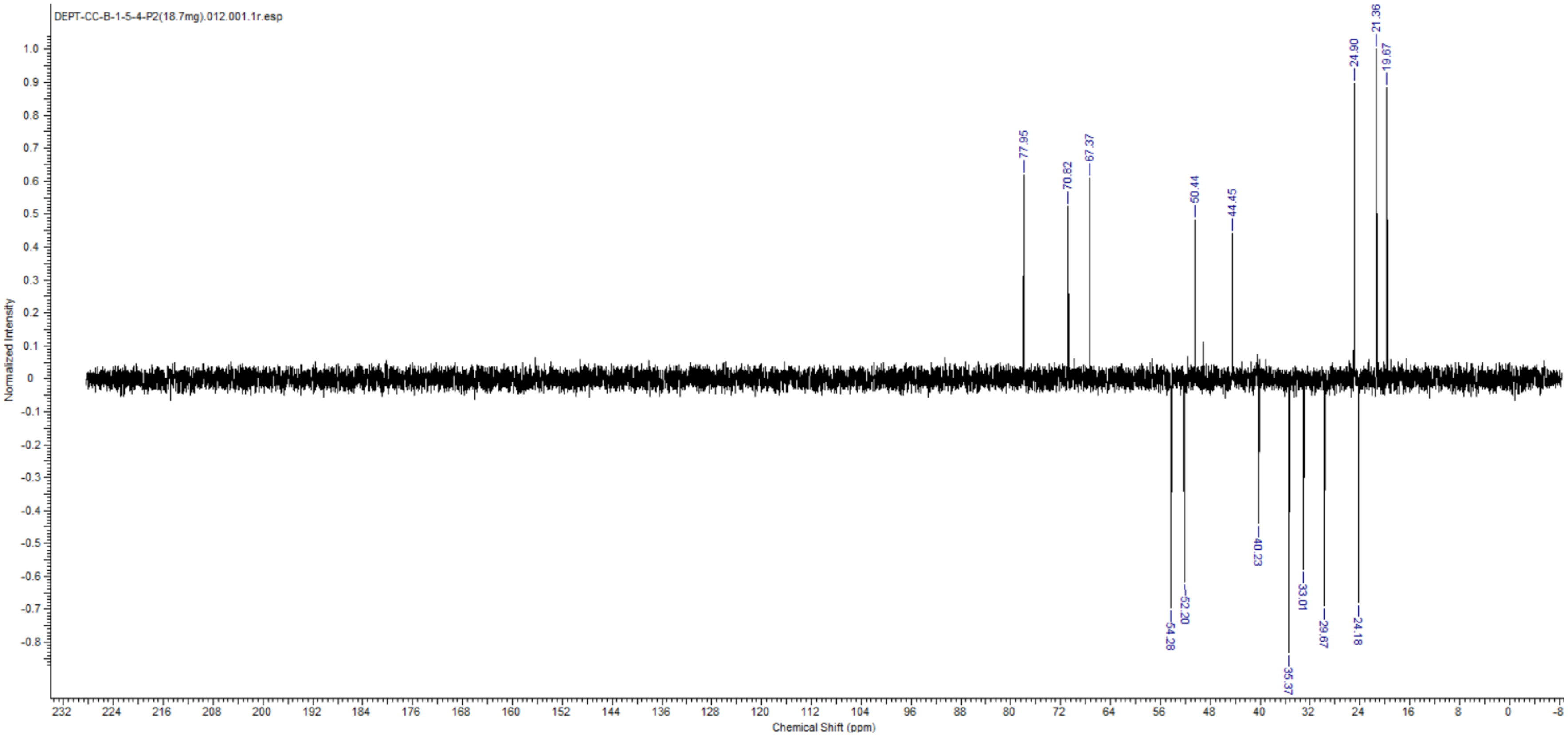

Figure S42. ^{13}C DEPT135 NMR spectrum of **5** in DMSO-d_6 (125 MHz)

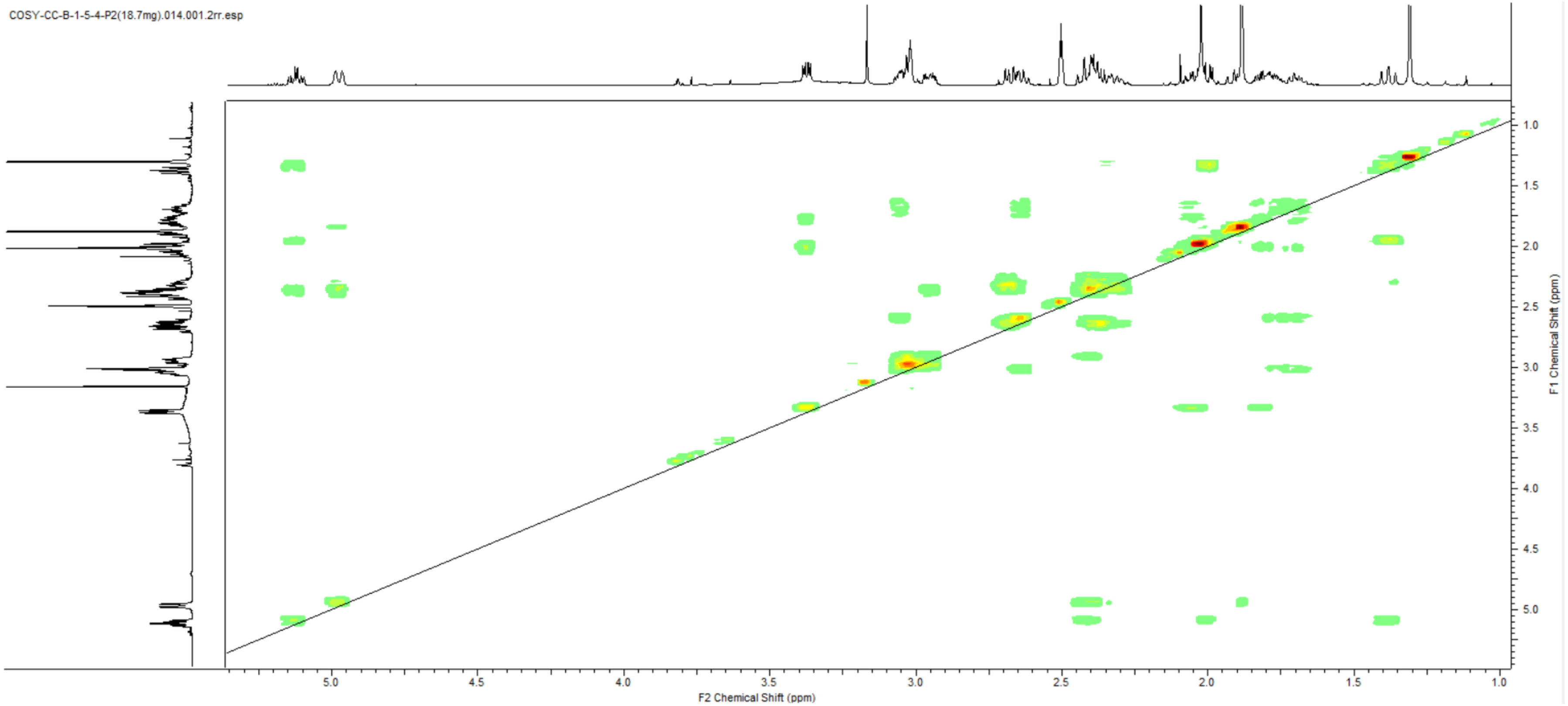

Figure S43. 1H-1H COSY spectrum of **5** in DMSO-d_6 (500 MHz)

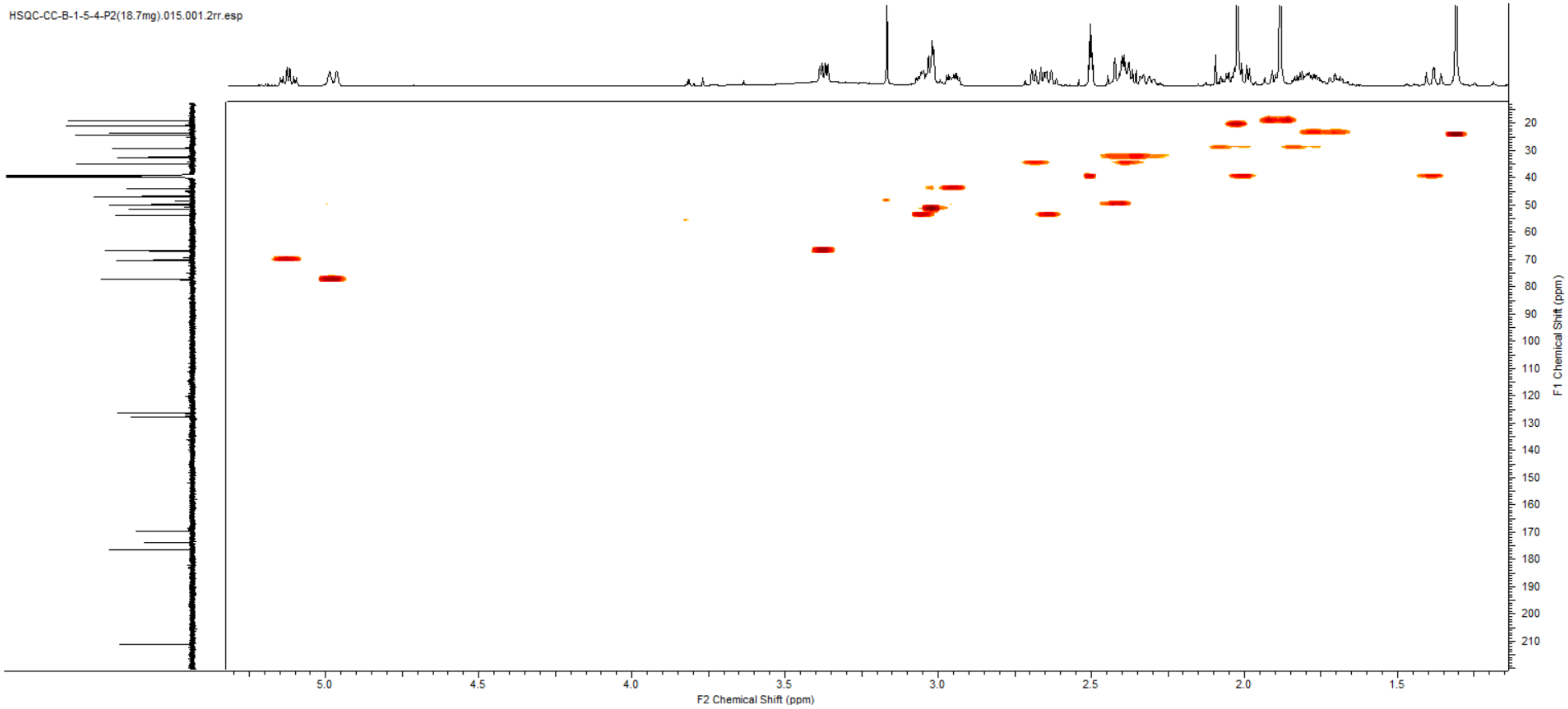

Figure S44. HSQC spectrum of **5** in DMSO-d_6 (500 MHz)

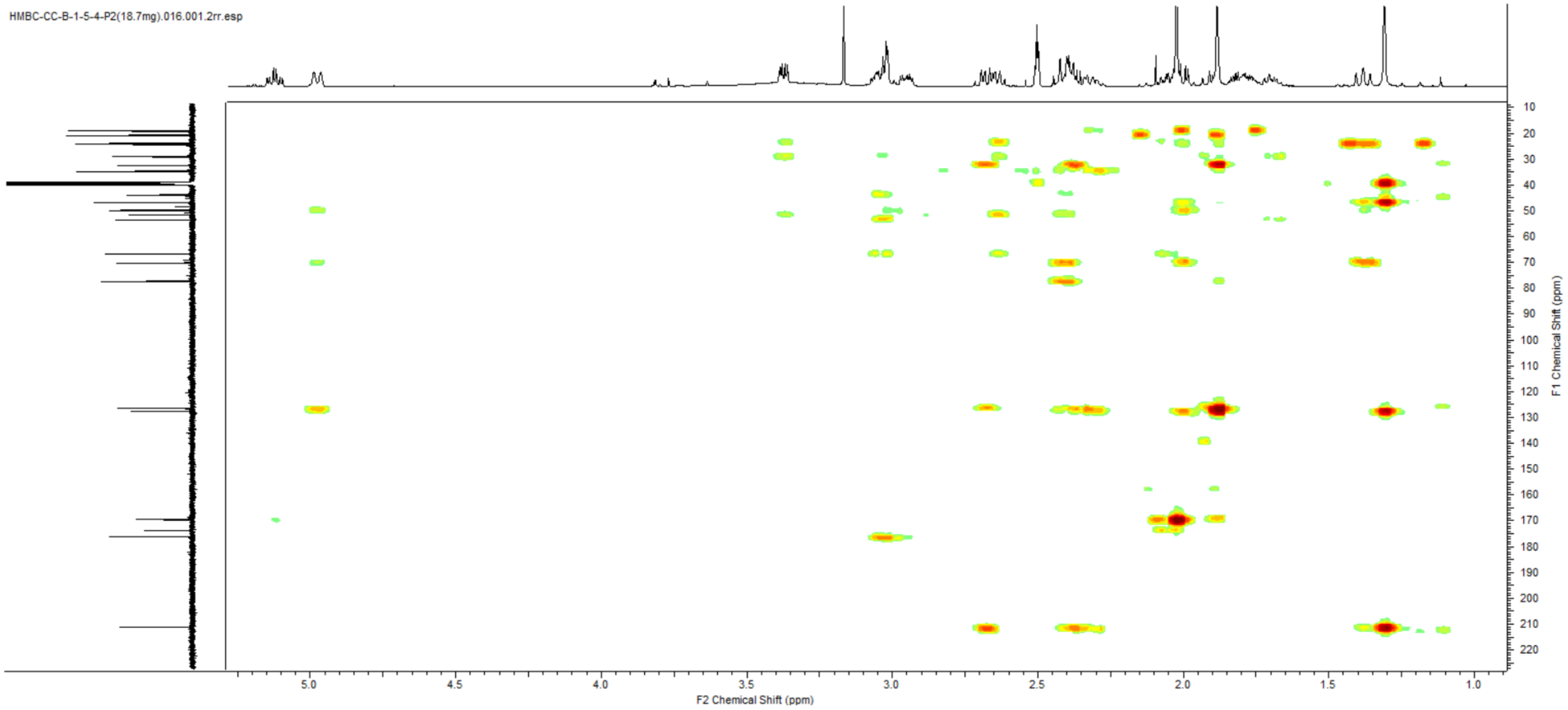

Figure S45. HMBC spectrum of **5** in DMSO-d_6 (500 MHz)

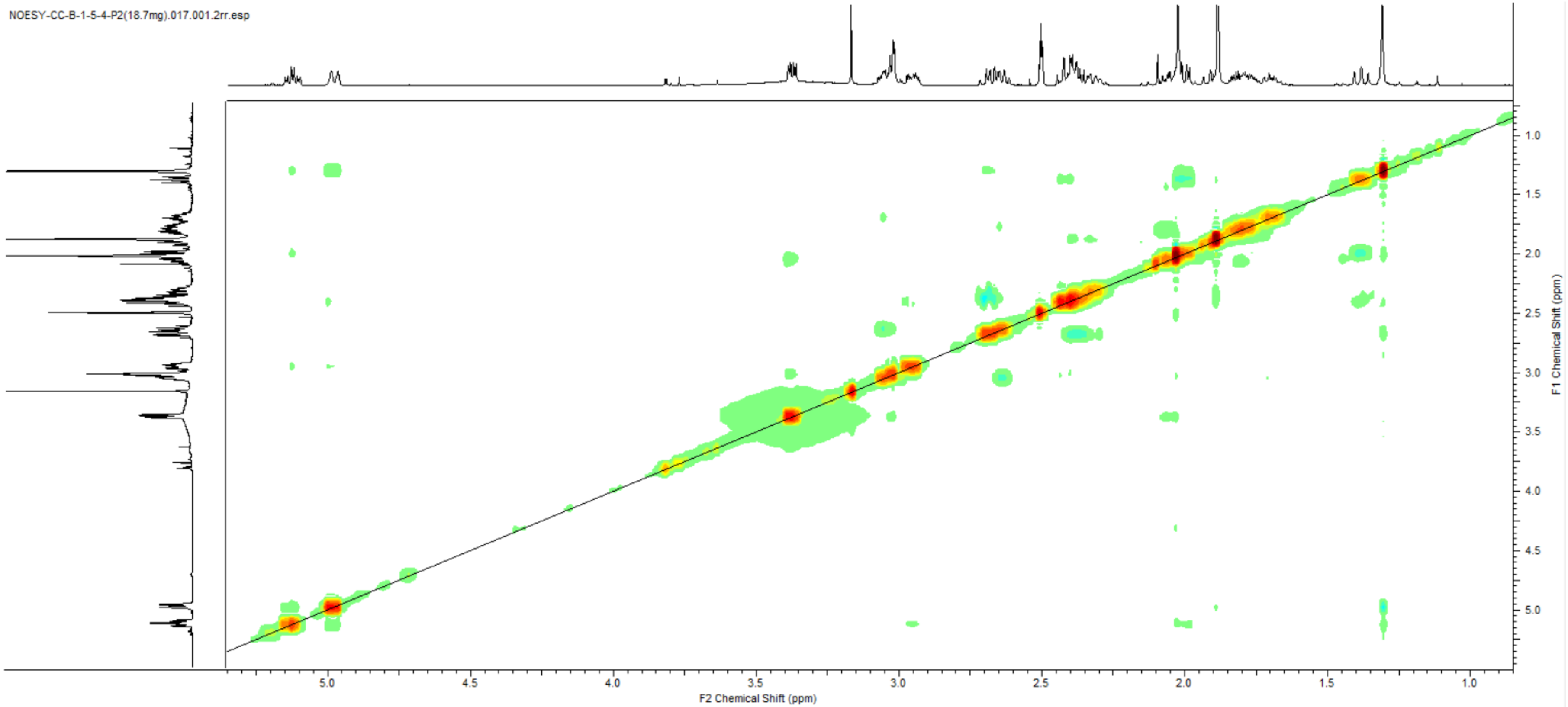

Figure S46. PS-NOESY spectrum of **5** in DMSO-d_6 (500 MHz)

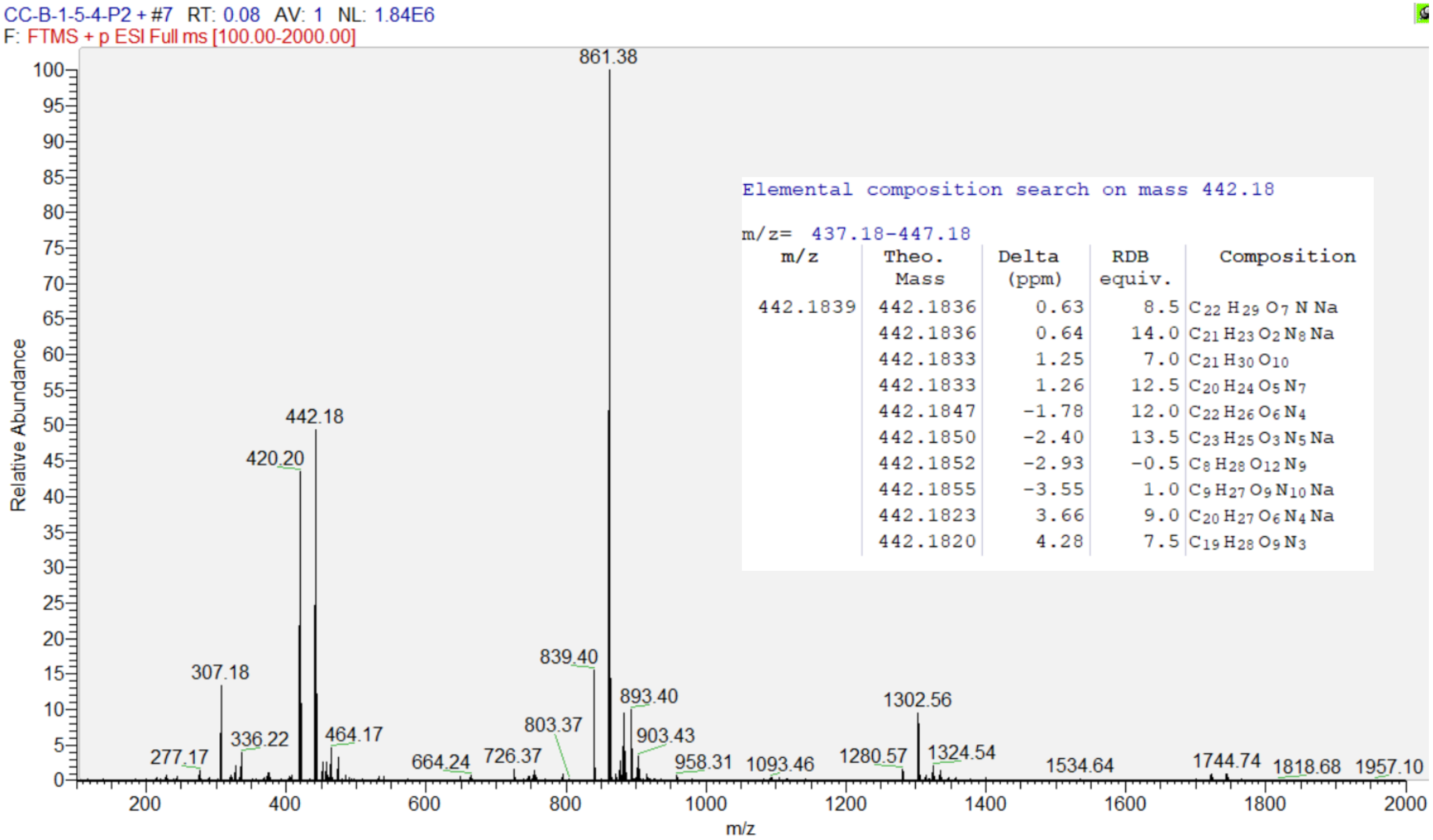

Figure S47. HR-ESI-MS spectrum of **5**

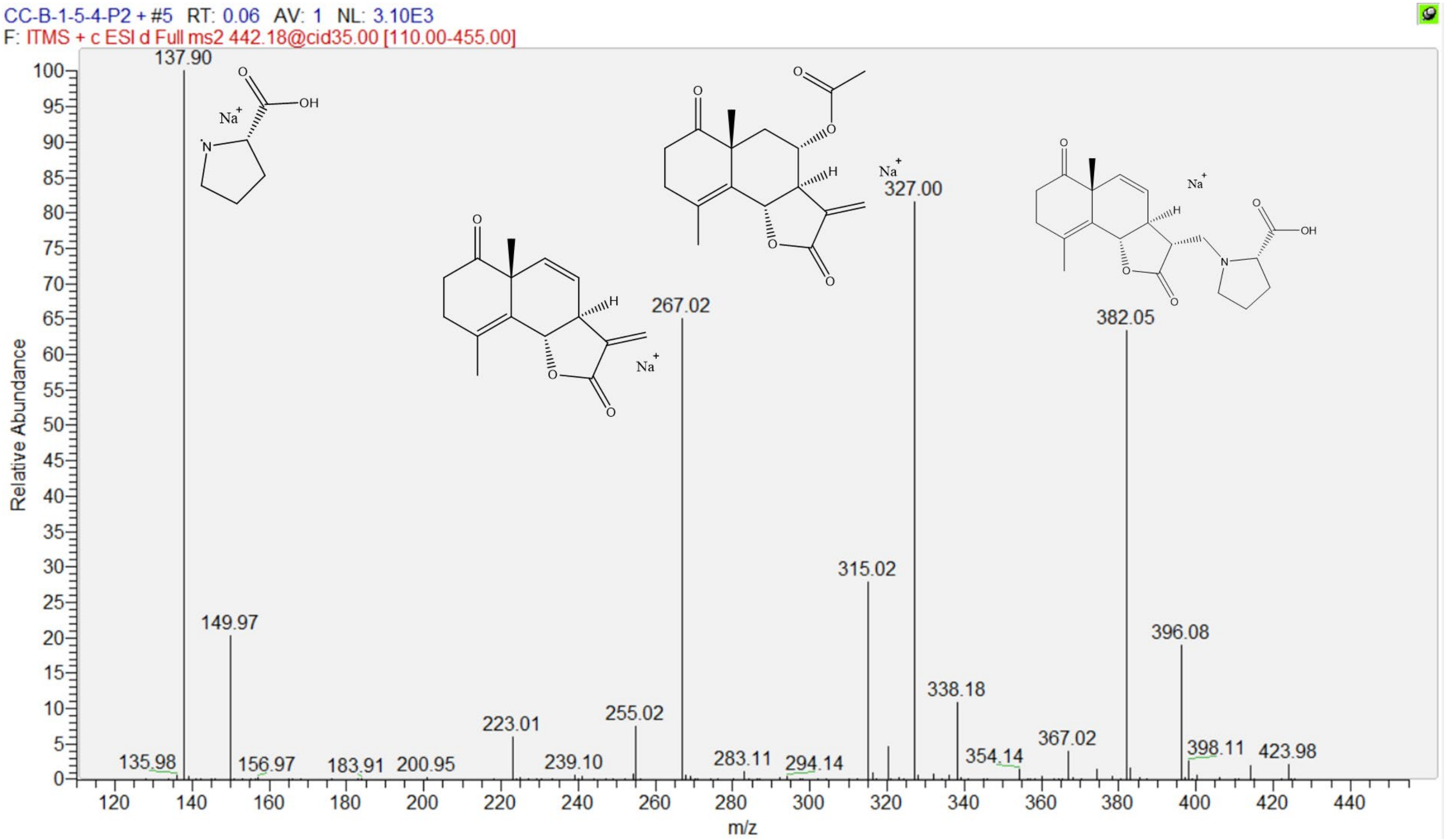

Figure S48. ESI-MS/MS spectrum of **5**

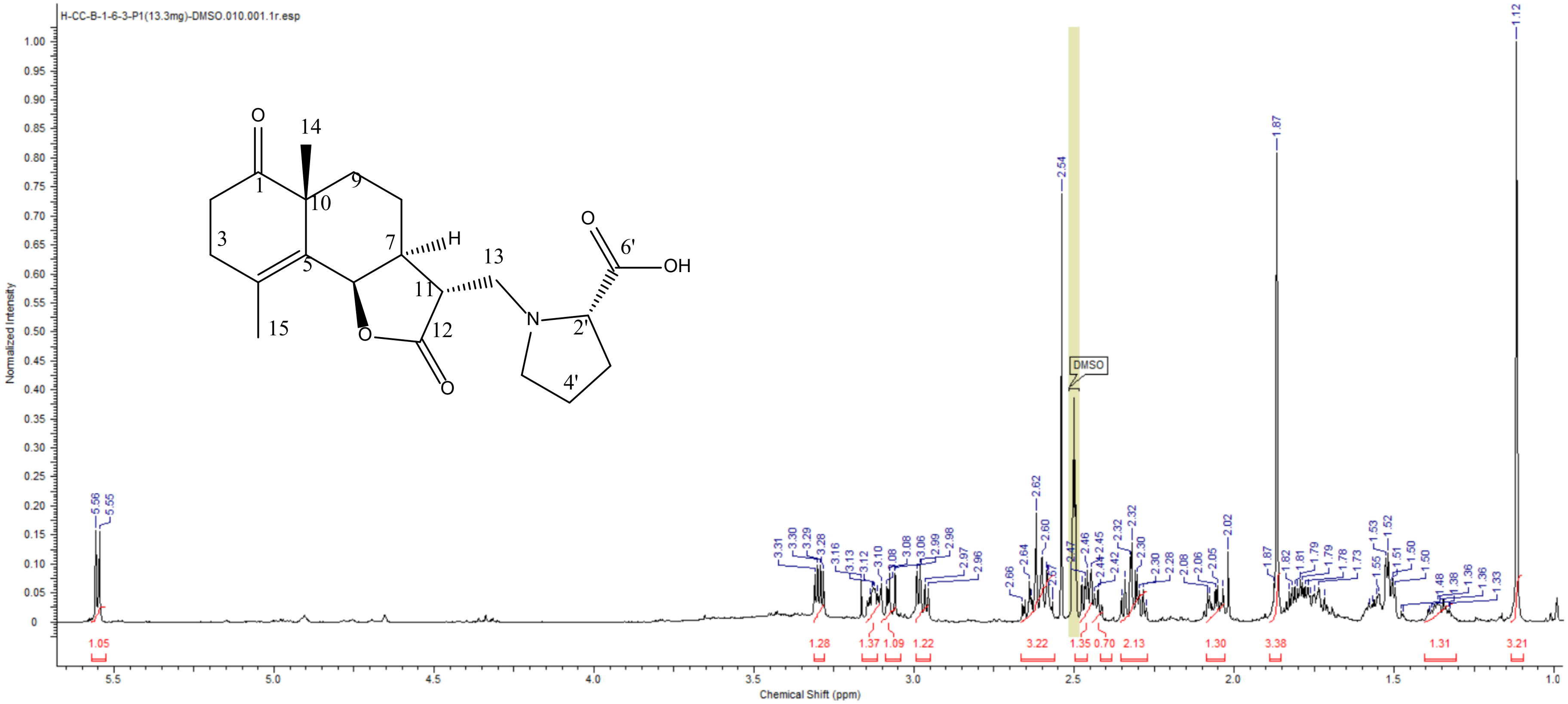

Figure S49. ^{1}H-NMR spectrum of **6** in DMSO-d_6 (500 MHz)

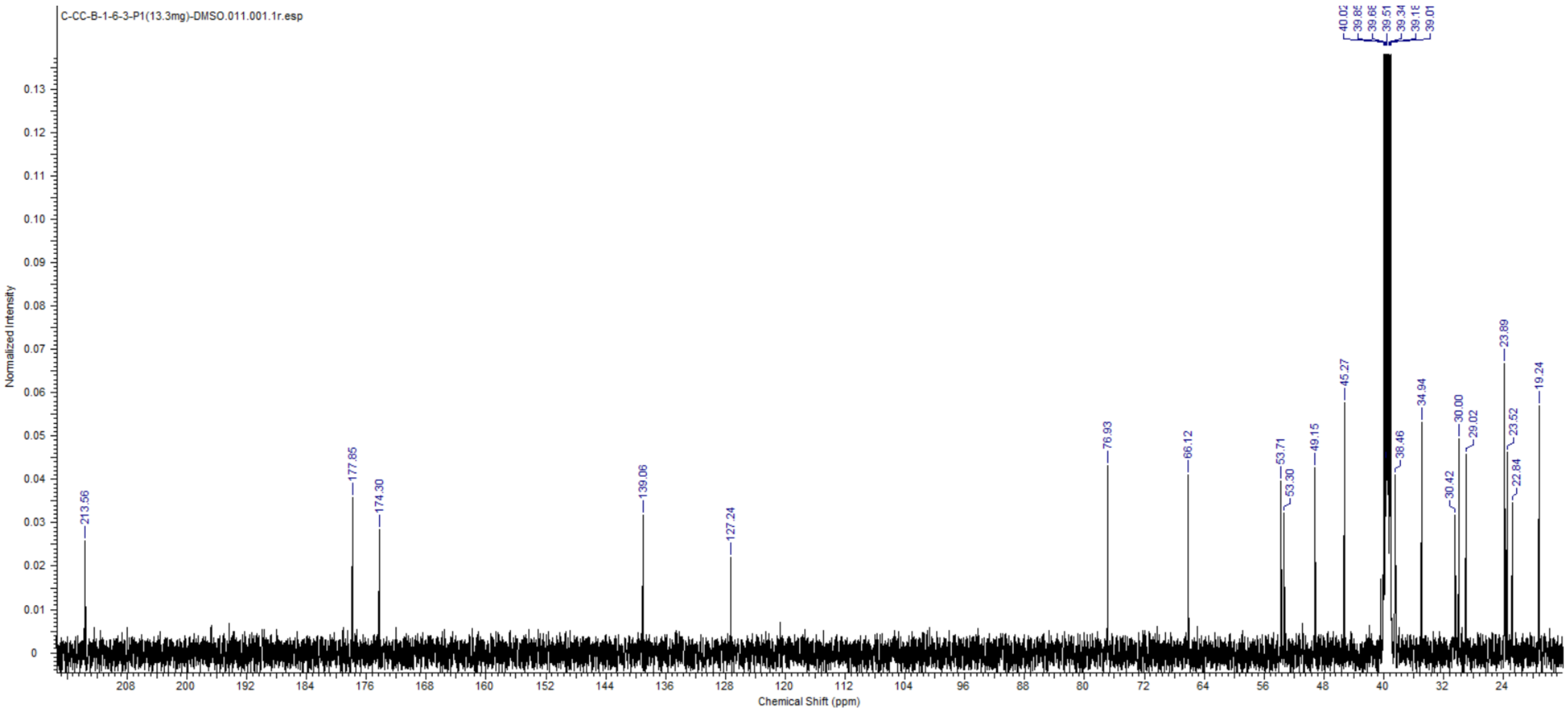

Figure S50. ^{13}C-NMR spectrum of **6** in DMSO-d_6 (125 MHz)

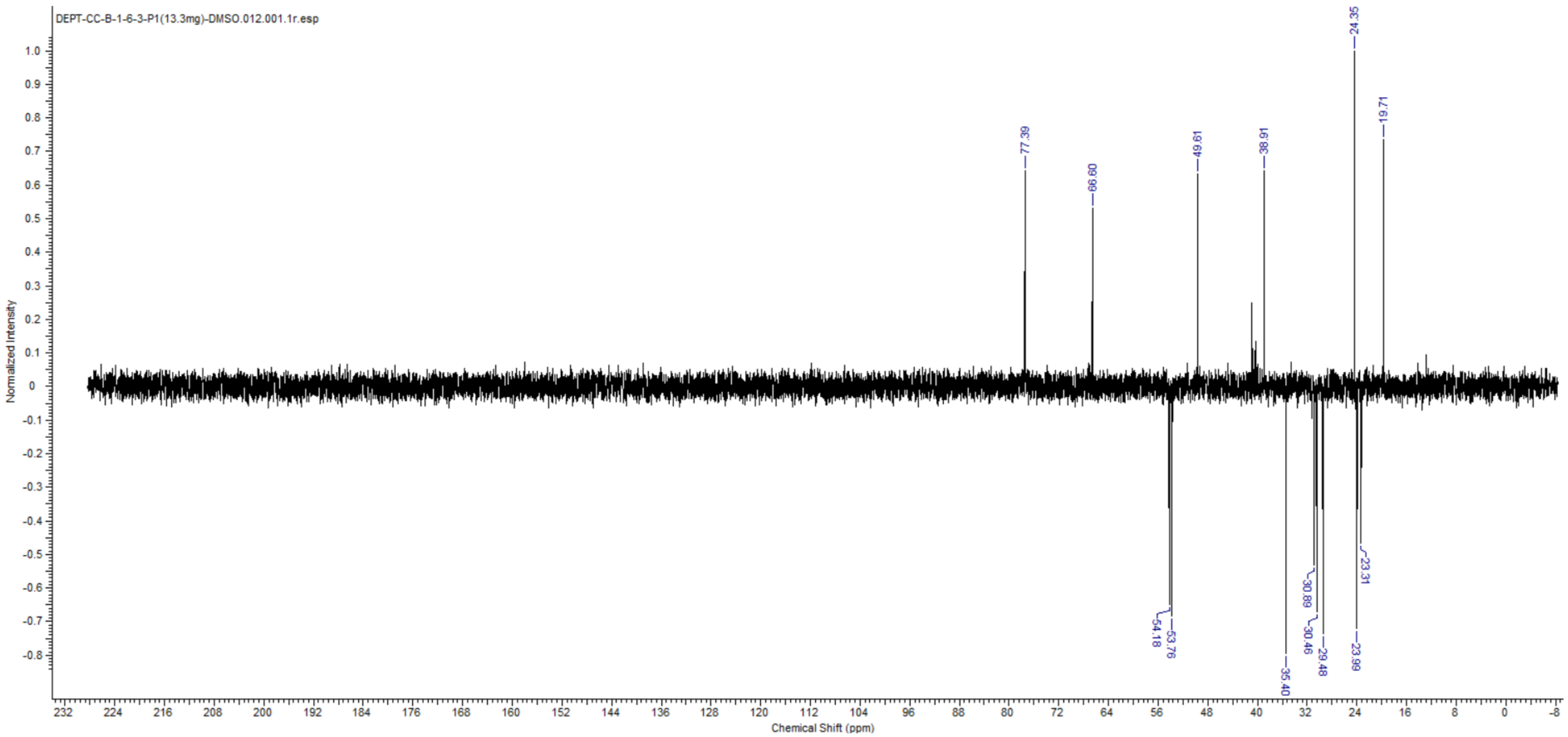

Figure S51. ^{13}C DEPT135 NMR spectrum of **6** in DMSO-d_6 (125 MHz)

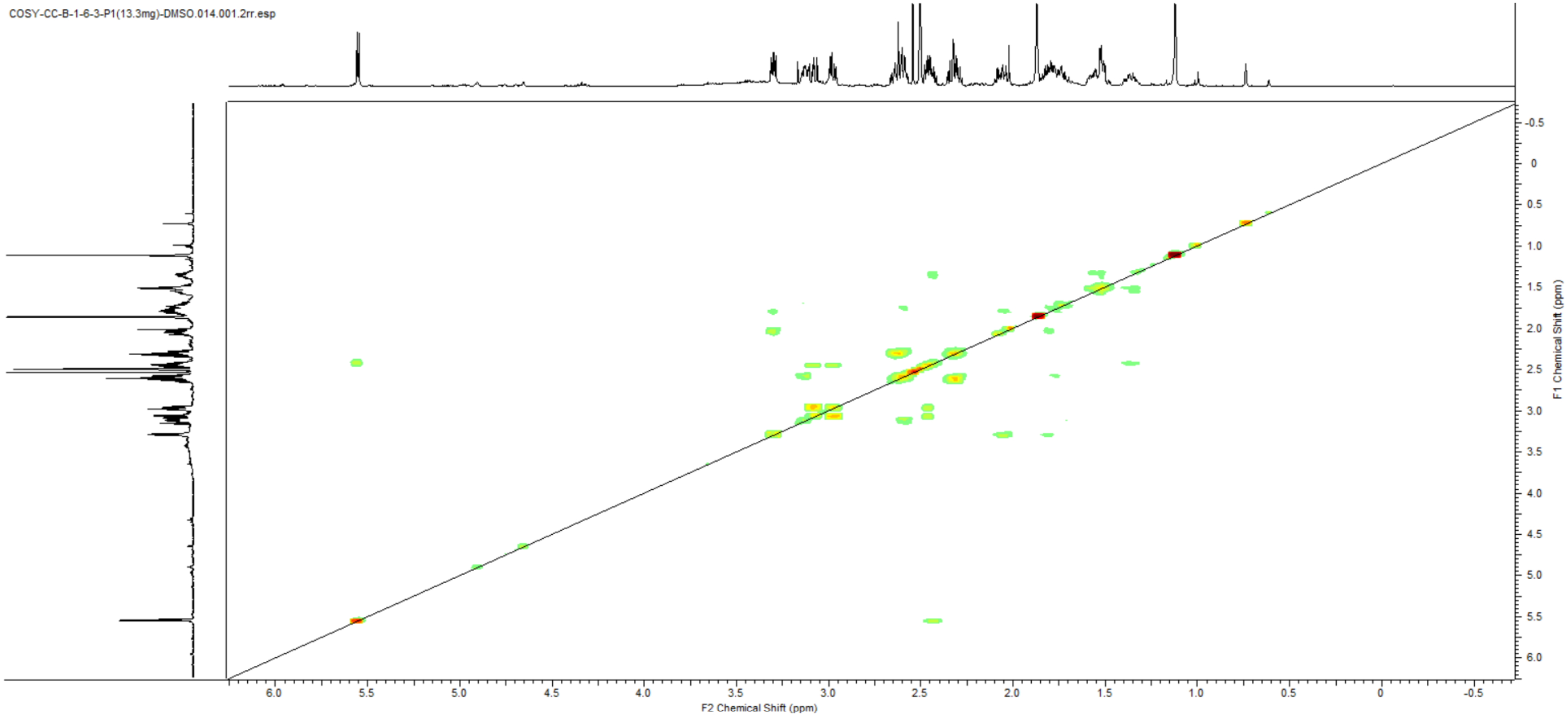

Figure S52. 1H-1H COSY spectrum of **6** in DMSO-*d*6 (500 MHz)

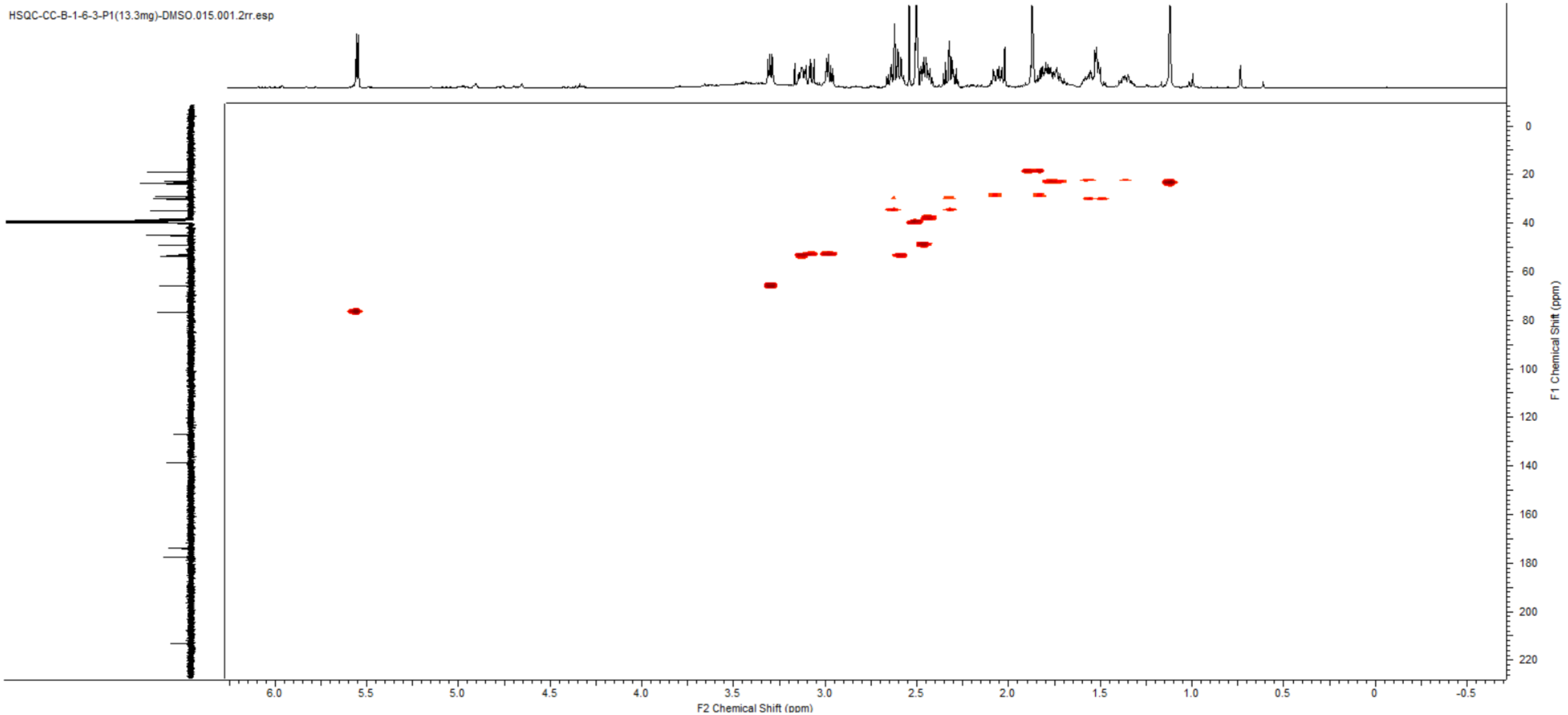

Figure S53. HSQC spectrum of **6** in DMSO-d_6 (500 MHz)

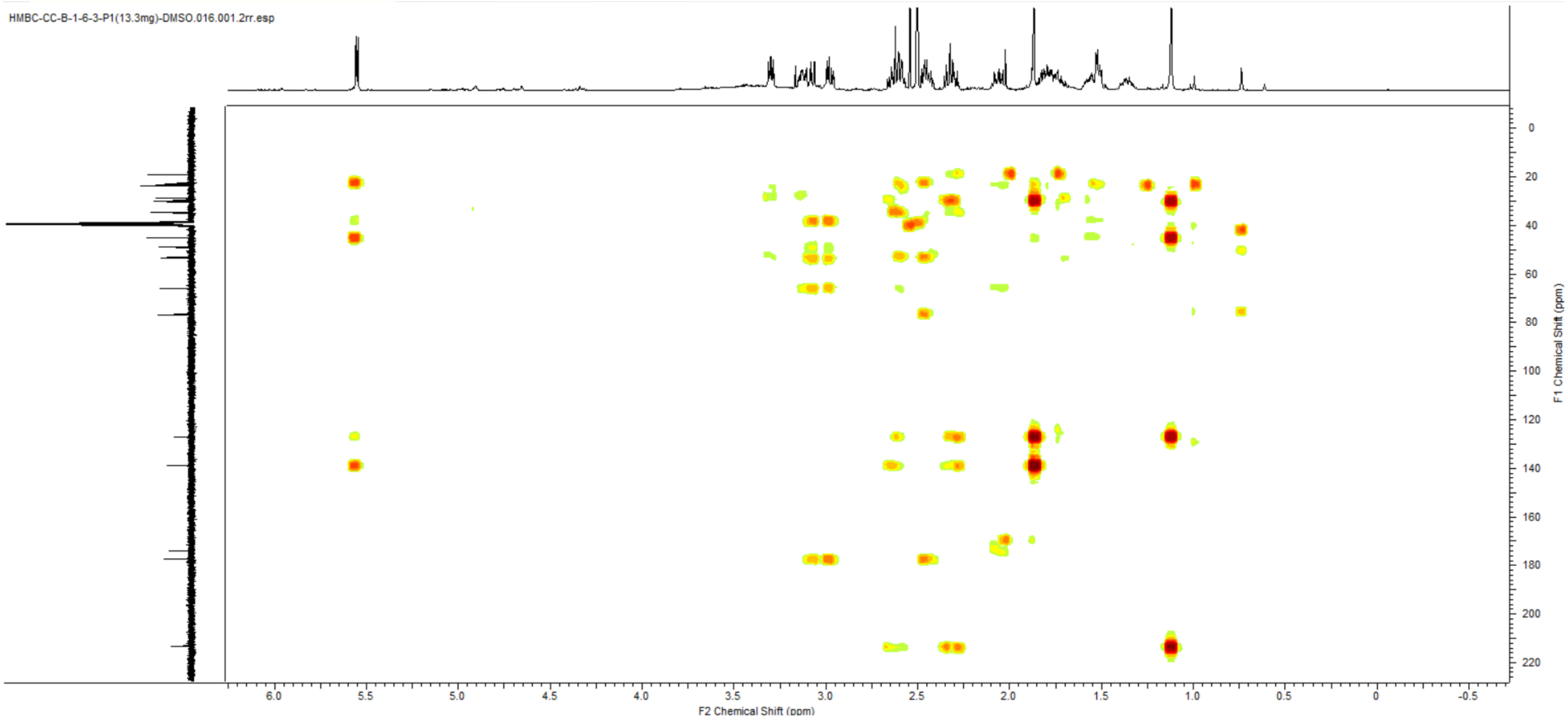

Figure S54. HMBC spectrum of **6** in DMSO-d_6 (500 MHz)

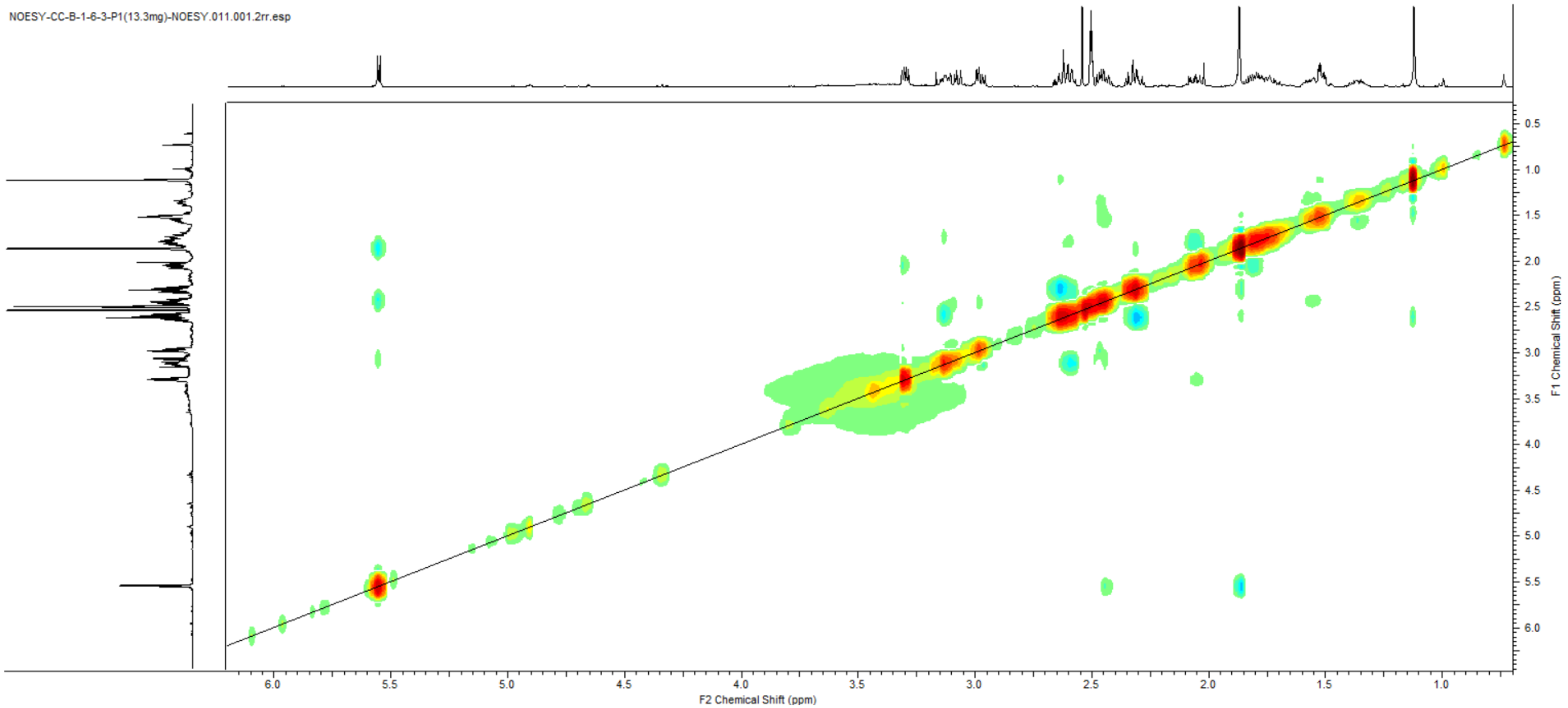

Figure S55. PS-NOESY spectrum of **6** in DMSO-d_6 (500 MHz)

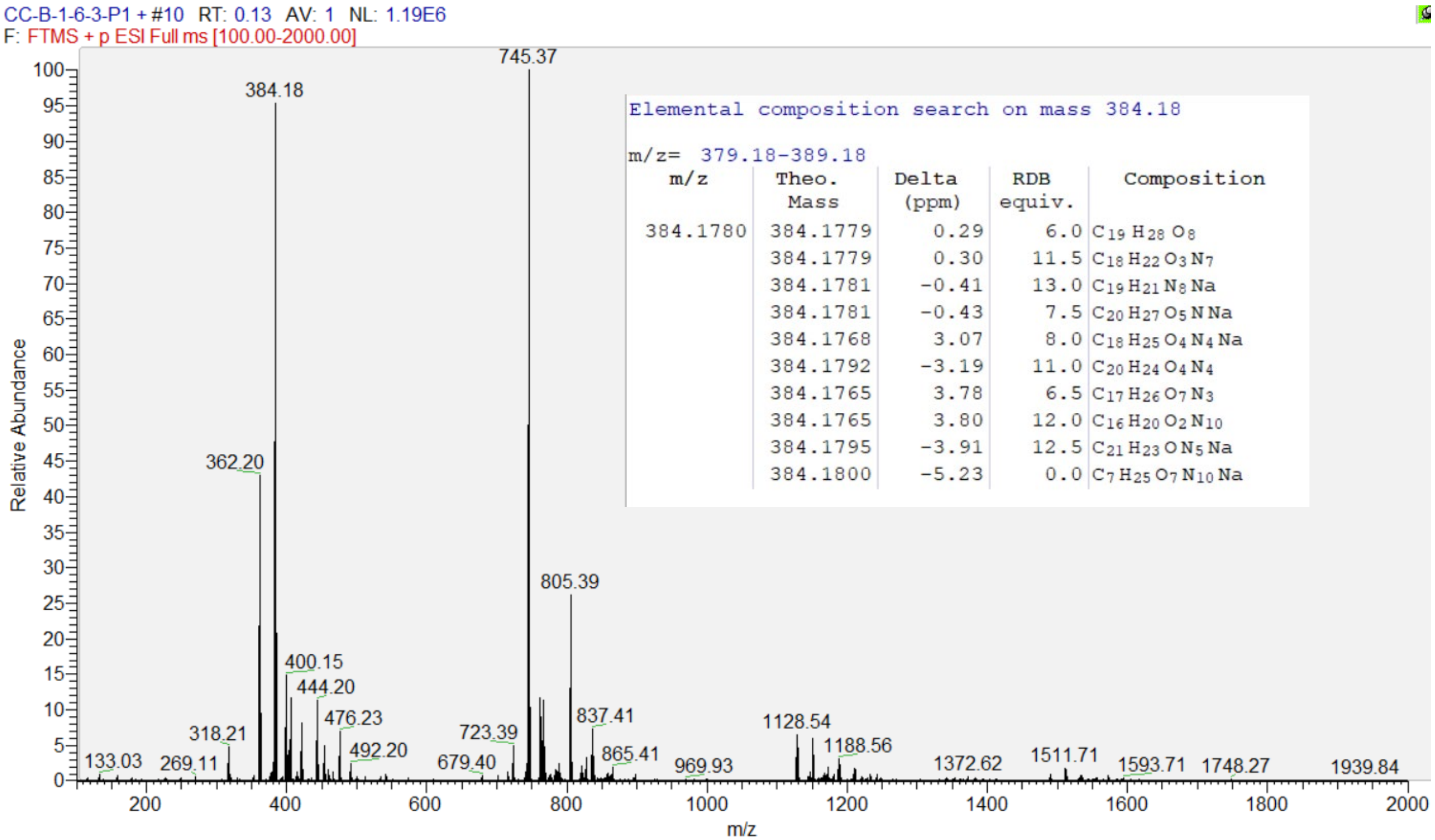

Elemental composition search on mass 384.18

m/z= 379.18-389.18

m/z	Theo. Mass	Delta (ppm)	RDB equiv.	Composition
384.1780	384.1779	0.29	6.0	$C_{19}H_{28}O_8$
	384.1779	0.30	11.5	$C_{18}H_{22}O_3N_7$
	384.1781	-0.41	13.0	$C_{19}H_{21}N_8Na$
	384.1781	-0.43	7.5	$C_{20}H_{27}O_5NNa$
	384.1768	3.07	8.0	$C_{18}H_{25}O_4N_4Na$
	384.1792	-3.19	11.0	$C_{20}H_{24}O_4N_4$
	384.1765	3.78	6.5	$C_{17}H_{26}O_7N_3$
	384.1765	3.80	12.0	$C_{16}H_{20}O_2N_{10}$
	384.1795	-3.91	12.5	$C_{21}H_{23}ON_5Na$
	384.1800	-5.23	0.0	$C_7H_{25}O_7N_{10}Na$

Figure S56. HR-ESI-MS spectrum of **6**

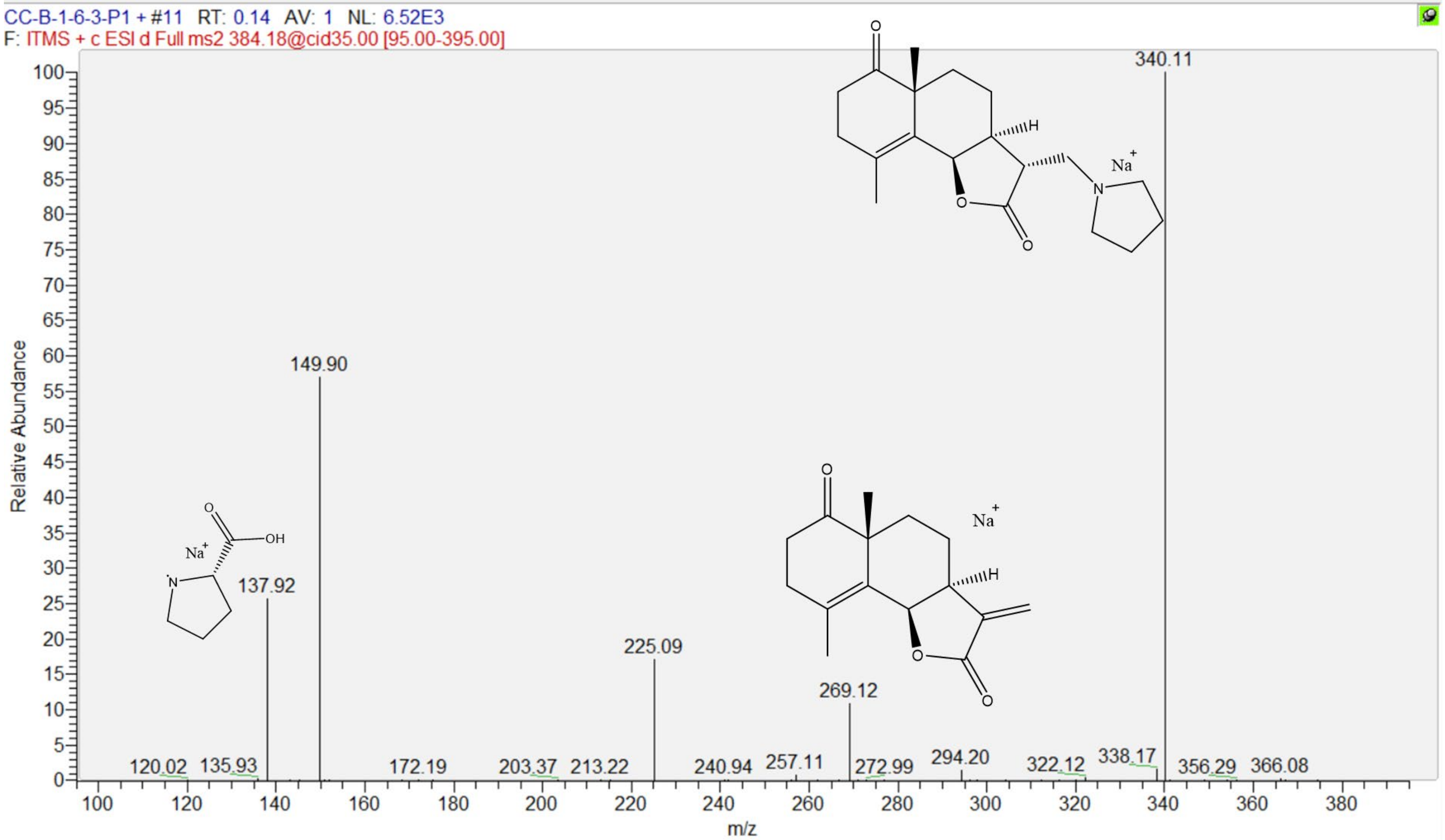

Figure S57. ESI-MS/MS spectrum of **6**

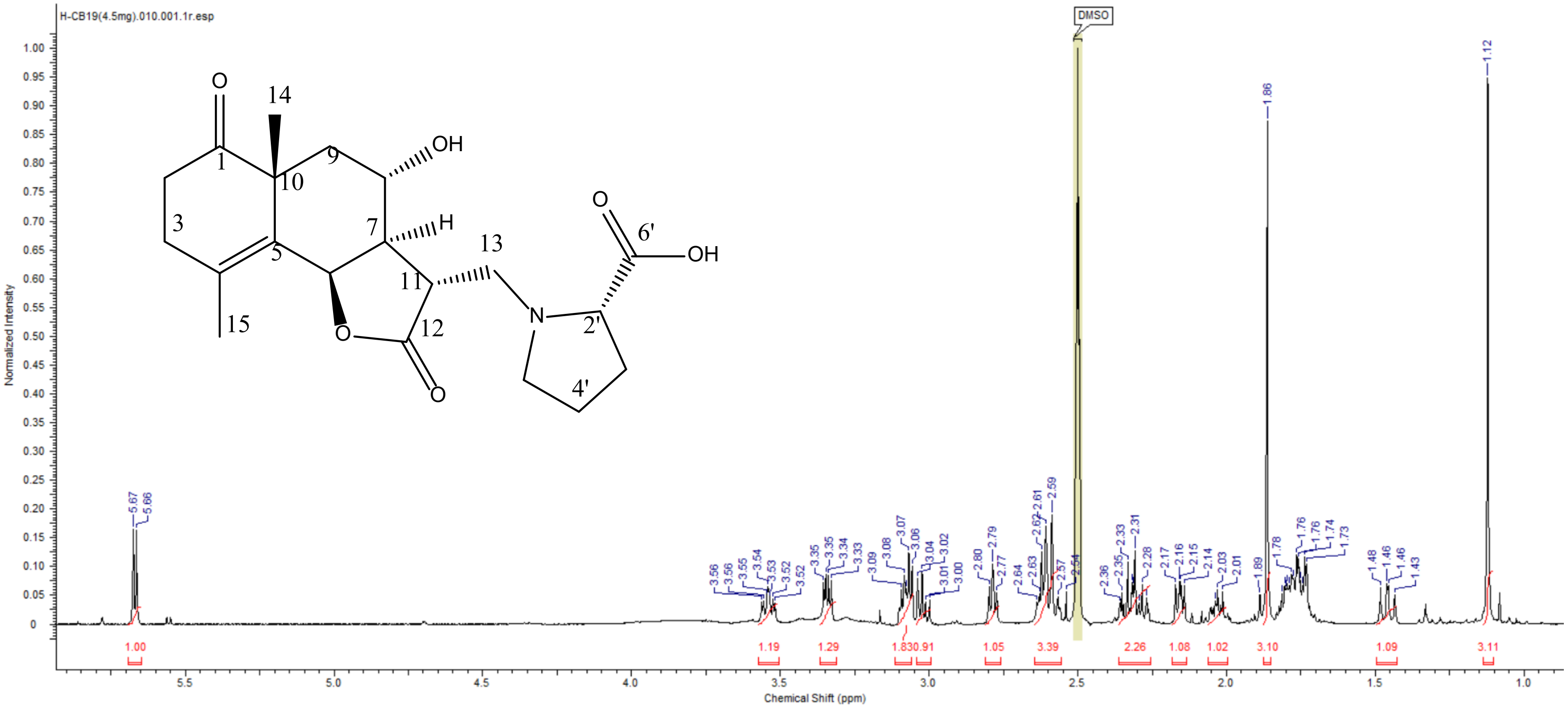

Figure S58. ^{1}H-NMR spectrum of **7** in DMSO-d_6 (500 MHz)

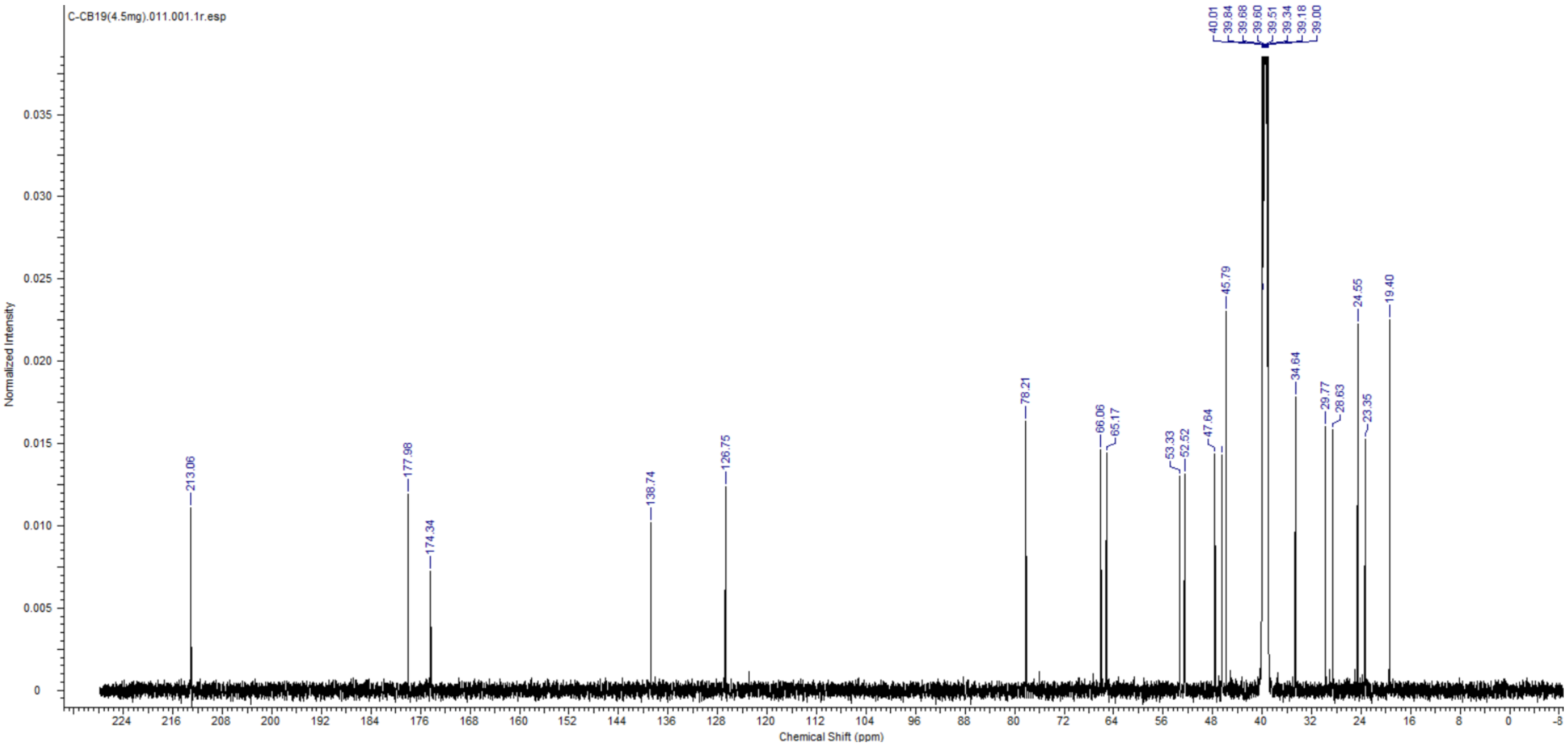

Figure S59. ^{13}C-NMR spectrum of **7** in DMSO-d_6 (125 MHz)

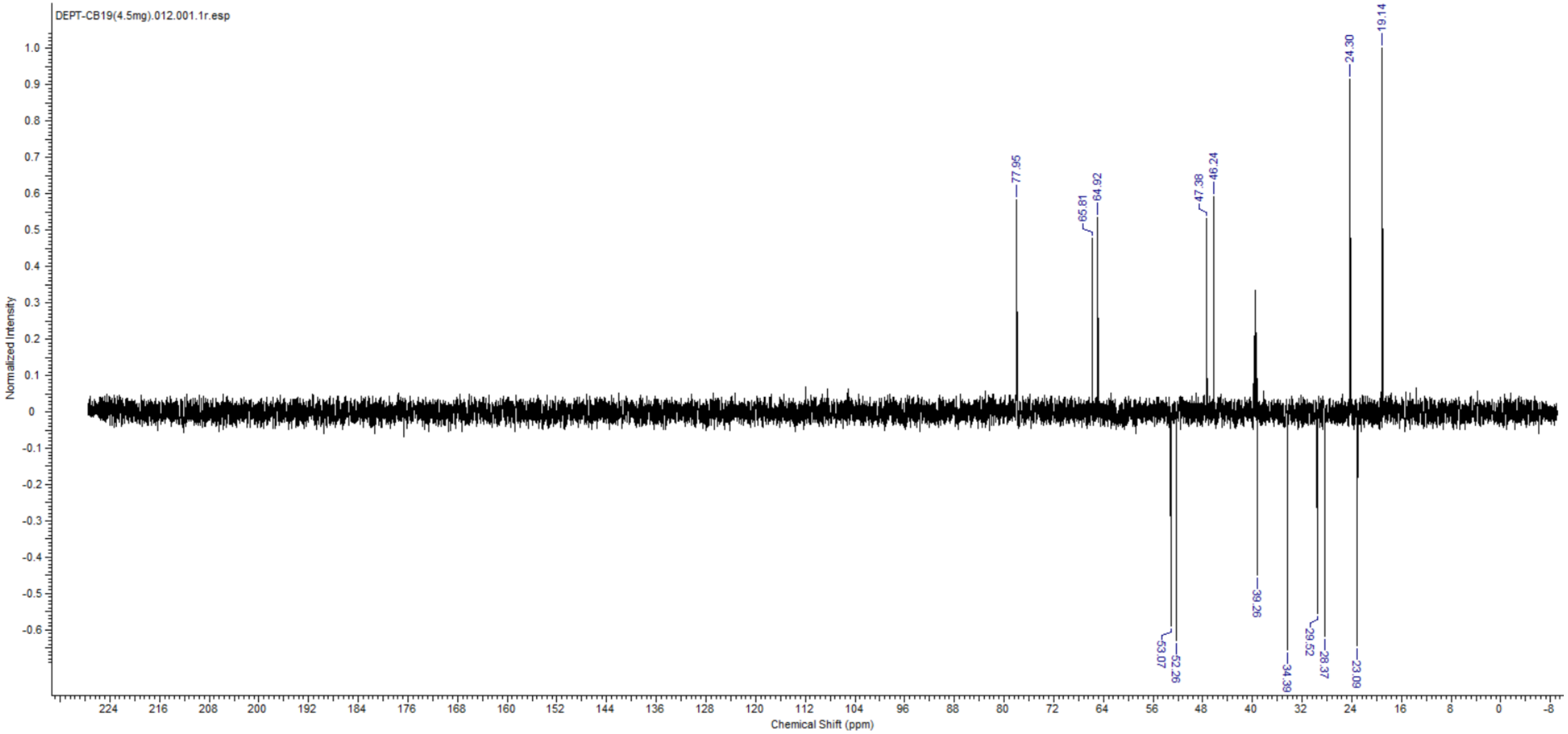

Figure S60. ^{13}C DEPT135 NMR spectrum of **7** in DMSO-d_6 (125 MHz)

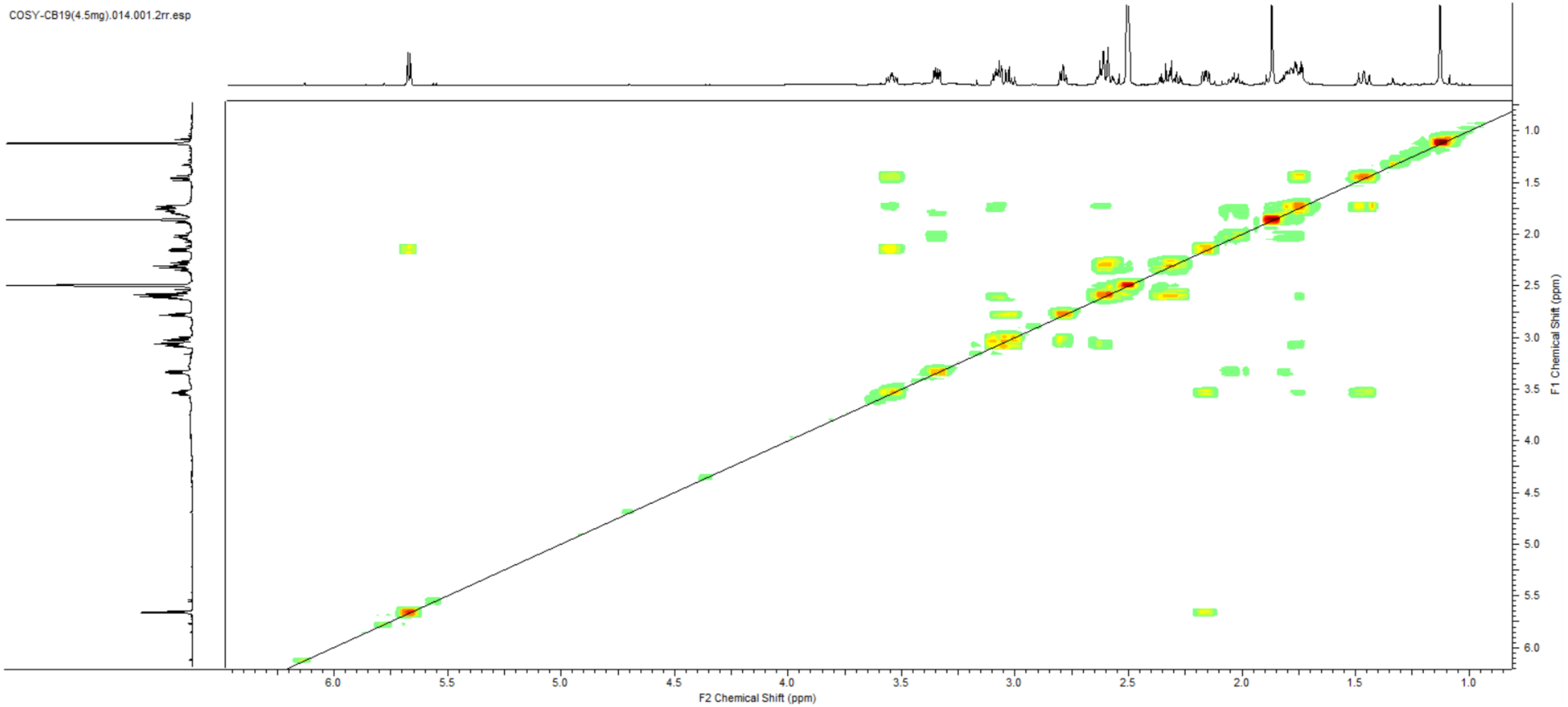

Figure S61. 1H-1H COSY spectrum of **7** in DMSO-d_6 (500 MHz)

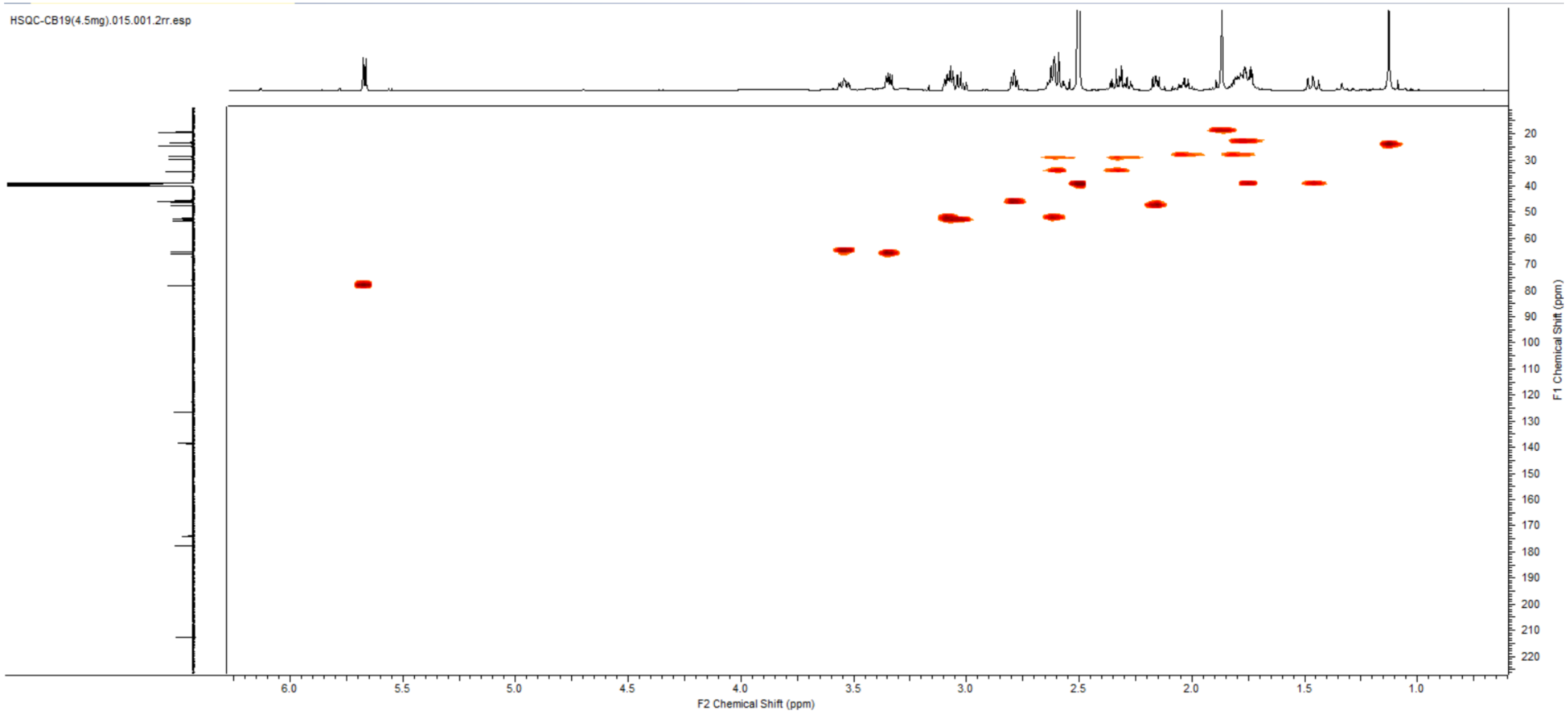

Figure S62. HSQC spectrum of **7** in DMSO-d_6 (500 MHz)

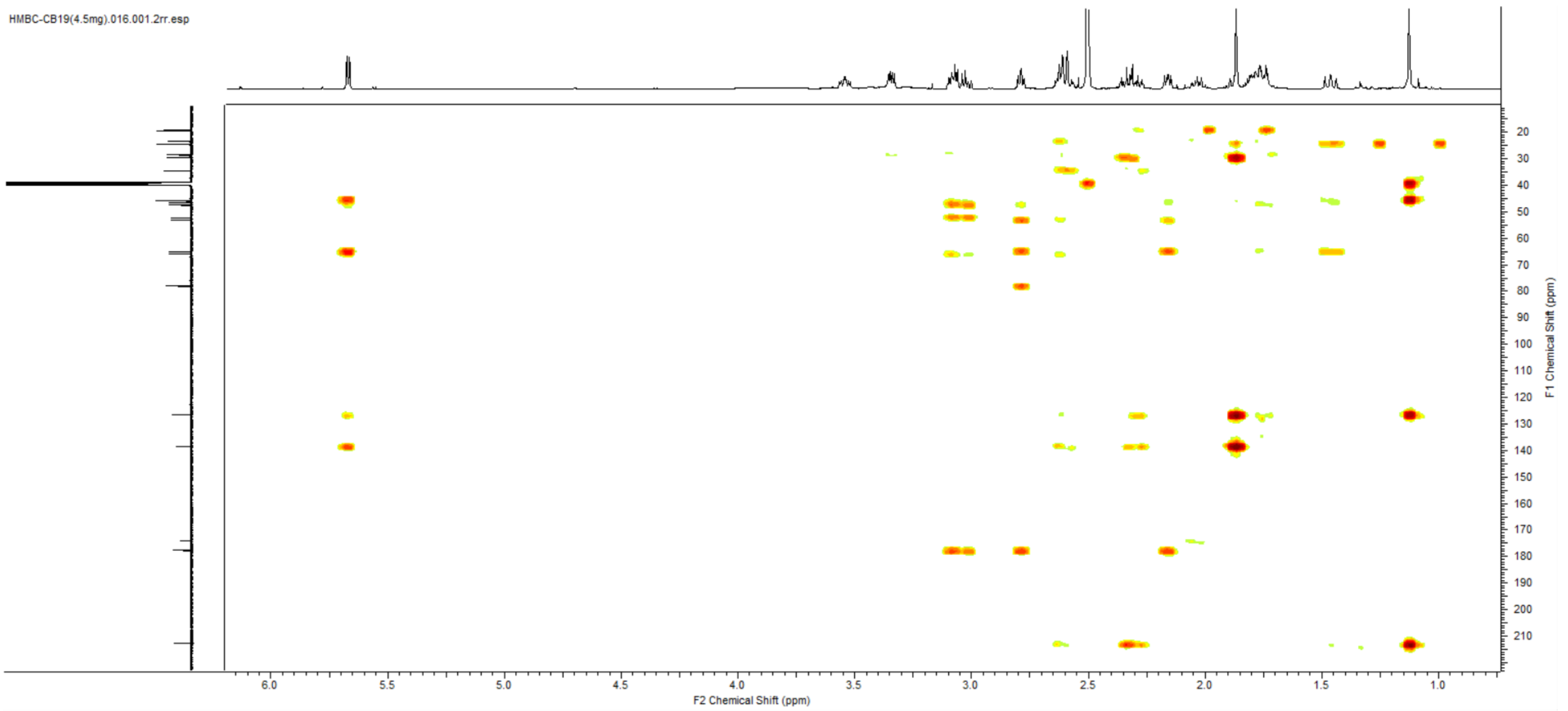

Figure S63. HMBC spectrum of **7** in DMSO-d_6 (500 MHz)

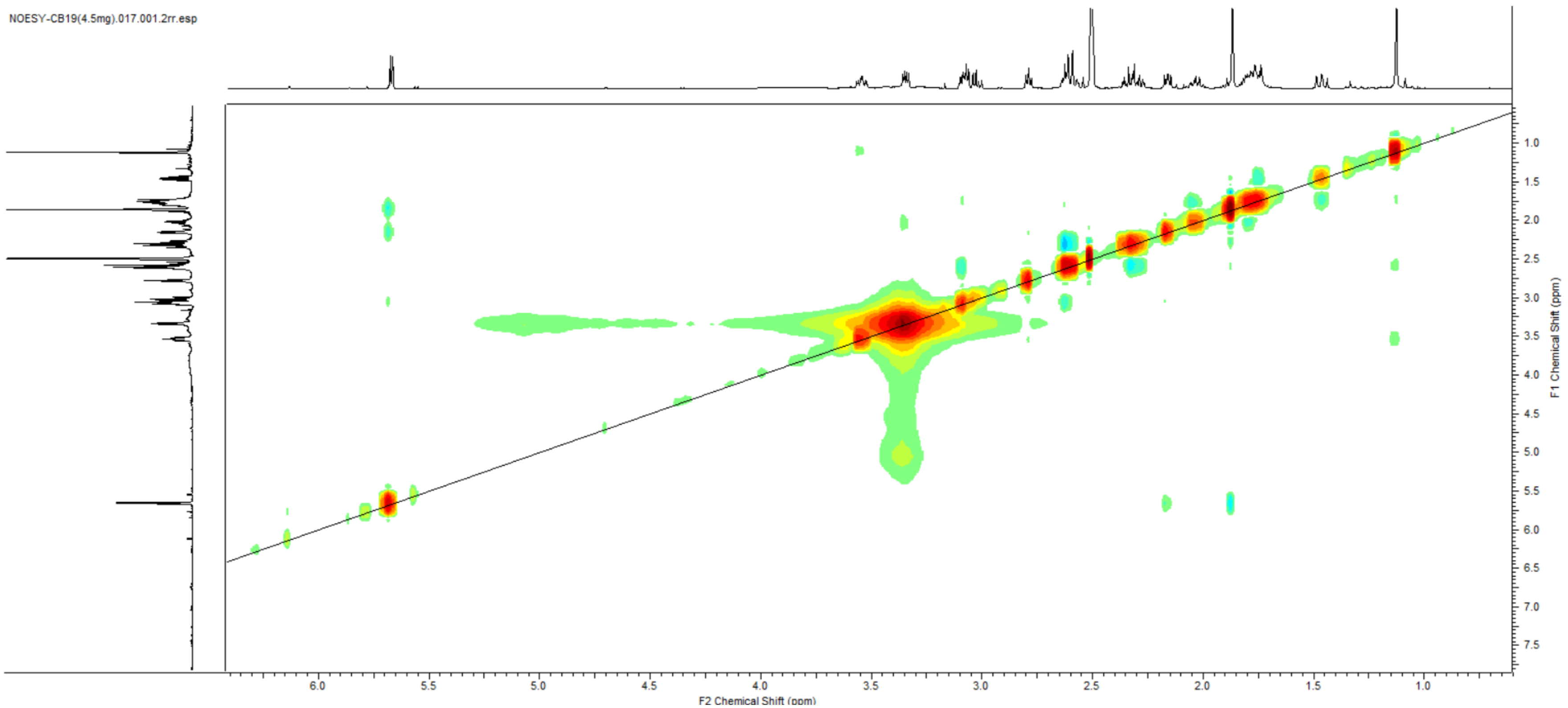

Figure S64. PS-NOESY spectrum of **7** in DMSO-d_6 (500 MHz)

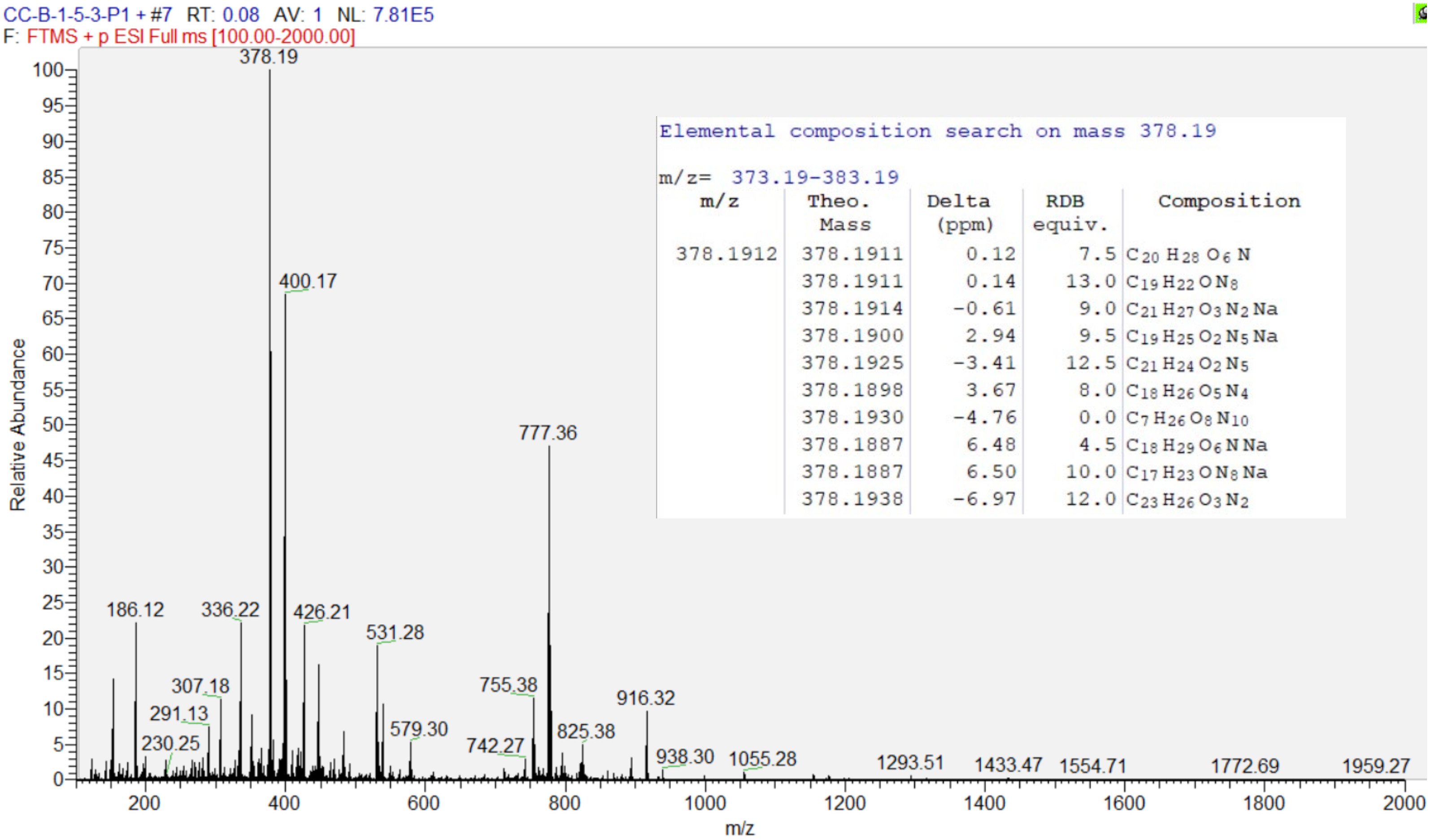

Figure S65. HR-ESI-MS spectrum of **7**

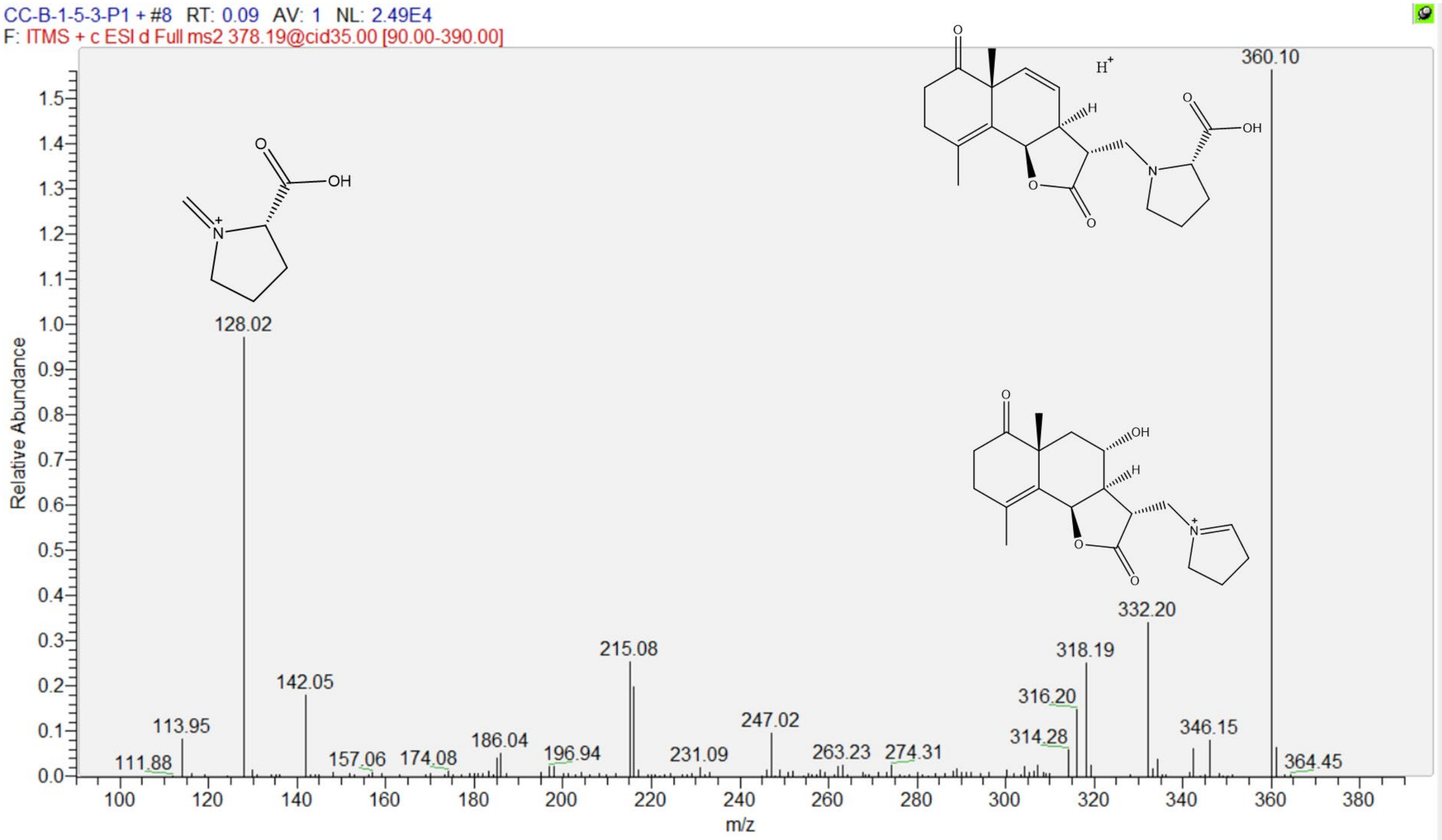

Figure S66. ESI-MS/MS spectrum of **7**

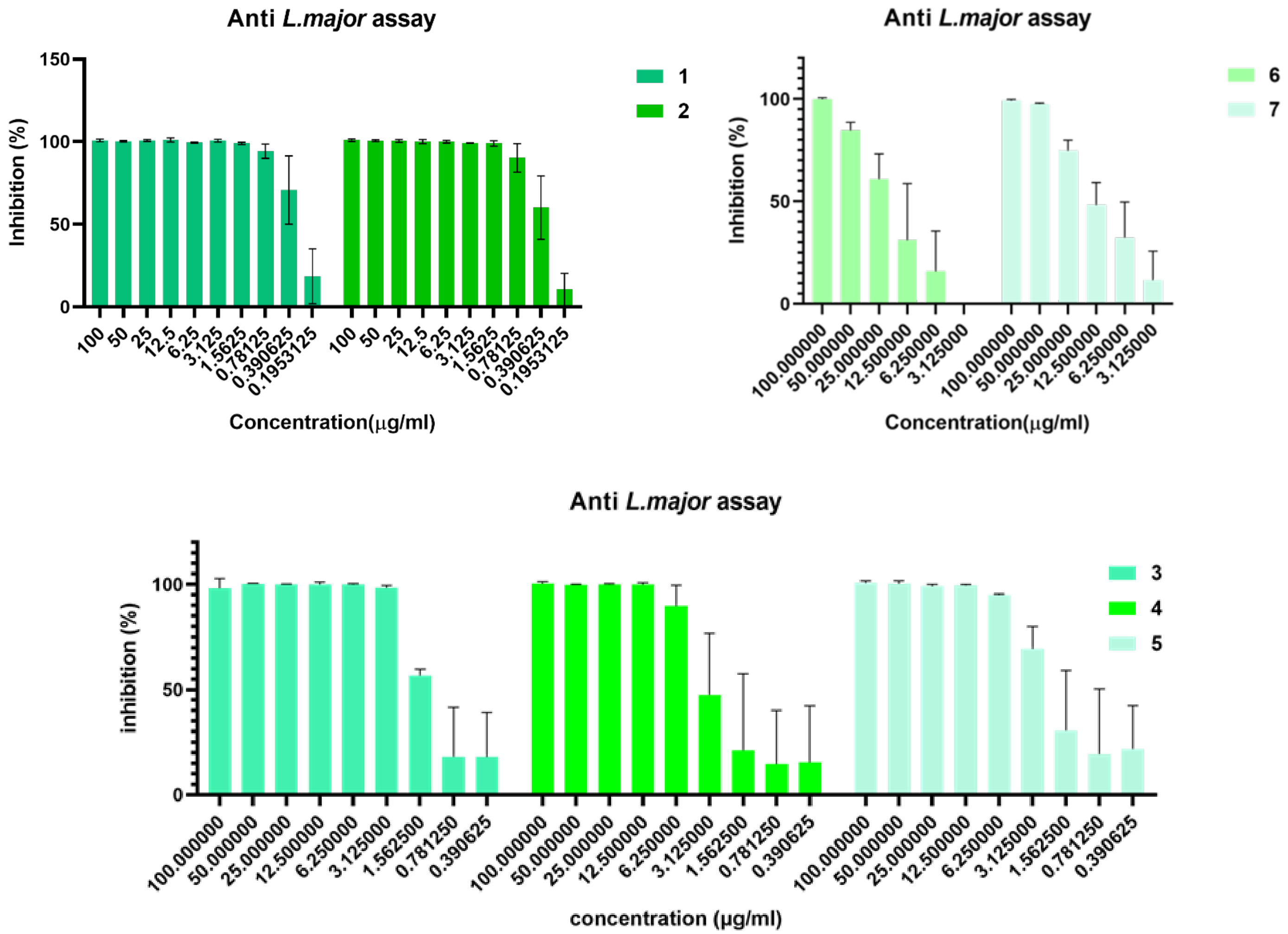

Figure S67. Anti-*L. major* data of **1-7**

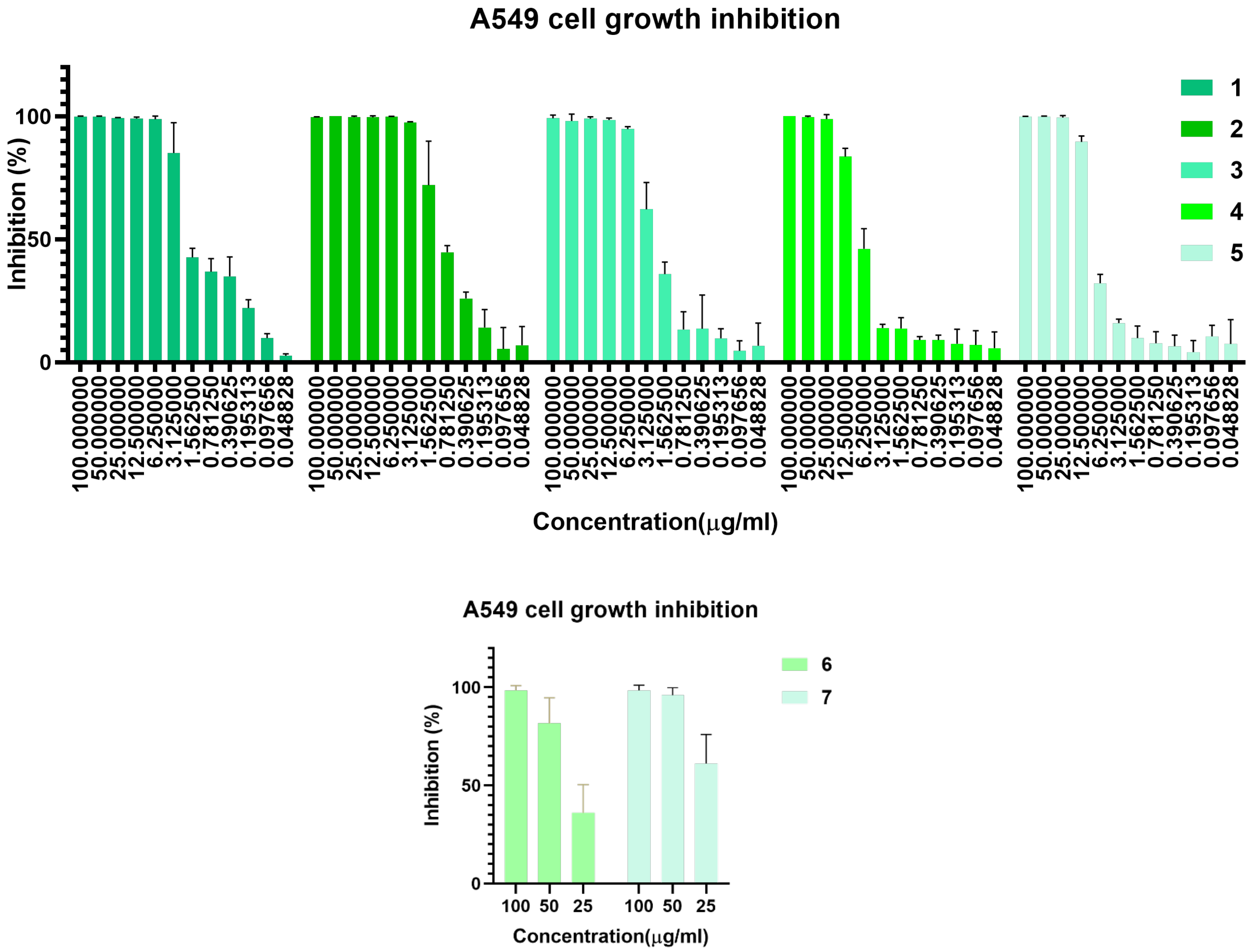

Figure S68. Anti-A549 data of **1**-**7**

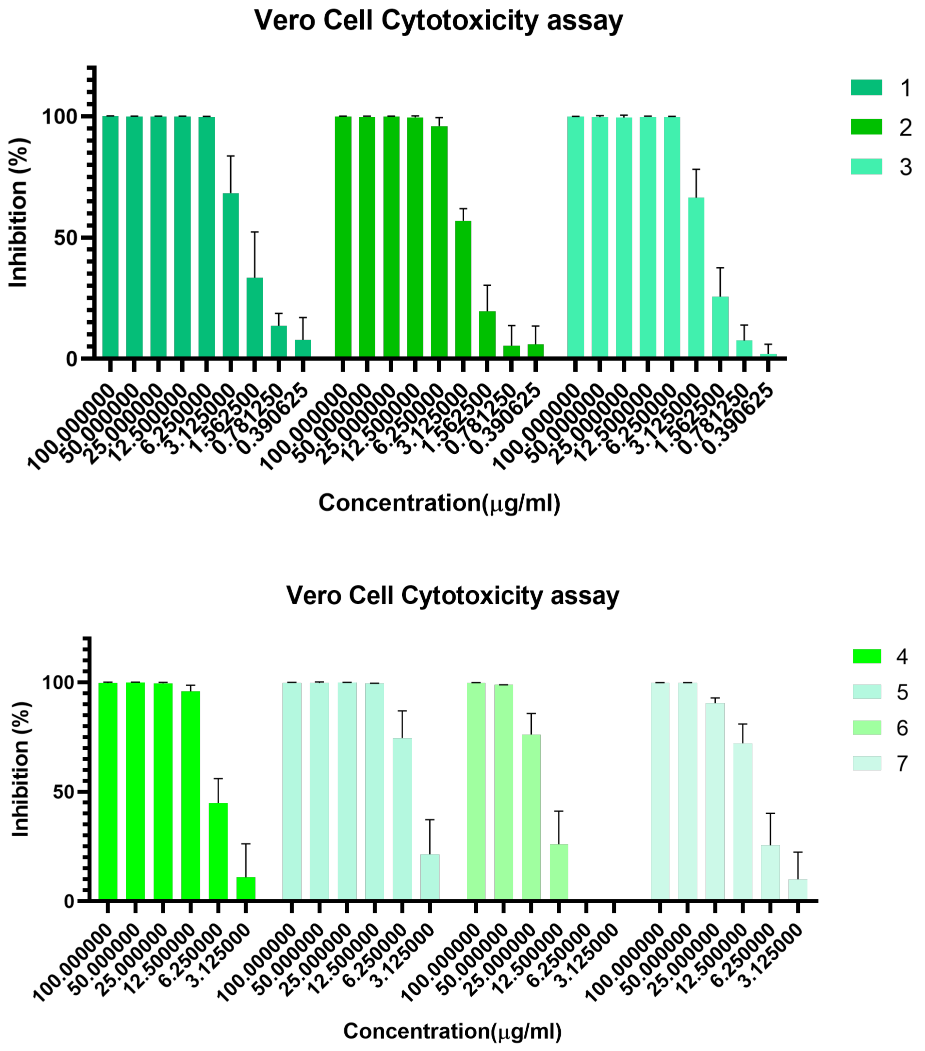

Figure S69. Vero cell cytotoxicity data of **1-7**
